# Analysis of five near-complete genome assemblies of the tomato pathogen *Cladosporium fulvum* uncovers additional accessory chromosomes and structural variations induced by transposable elements effecting the loss of avirulence genes

**DOI:** 10.1186/s12915-024-01818-z

**Published:** 2024-01-29

**Authors:** Alex Z. Zaccaron, Ioannis Stergiopoulos

**Affiliations:** Department of Plant Pathology, University of California Davis, Davis, CA 95616-8751 USA

**Keywords:** Chromosome translocation, Pangenome, Two-speed genome, Genome evolution, Gene loss, Transposons, Repeat-induced point mutations, Dispensable chromosome, Pseudogenization, Effectors

## Abstract

**Background:**

Fungal plant pathogens have dynamic genomes that allow them to rapidly adapt to adverse conditions and overcome host resistance. One way by which this dynamic genome plasticity is expressed is through effector gene loss, which enables plant pathogens to overcome recognition by cognate resistance genes in the host. However, the exact nature of these loses remains elusive in many fungi. This includes the tomato pathogen *Cladosporium fulvum*, which is the first fungal plant pathogen from which avirulence (*Avr*) genes were ever cloned and in which loss of *Avr* genes is often reported as a means of overcoming recognition by cognate tomato *Cf* resistance genes. A recent near-complete reference genome assembly of *C. fulvum* isolate Race 5 revealed a compartmentalized genome architecture and the presence of an accessory chromosome, thereby creating a basis for studying genome plasticity in fungal plant pathogens and its impact on avirulence genes.

**Results:**

Here, we obtained near-complete genome assemblies of four additional *C. fulvum* isolates. The genome assemblies had similar sizes (66.96 to 67.78 Mb), number of predicted genes (14,895 to 14,981), and estimated completeness (98.8 to 98.9%). Comparative analysis that included the genome of isolate Race 5 revealed high levels of synteny and colinearity, which extended to the density and distribution of repetitive elements and of repeat-induced point (RIP) mutations across homologous chromosomes. Nonetheless, structural variations, likely mediated by transposable elements and effecting the deletion of the avirulence genes *Avr4E*, *Avr5*, and *Avr9*, were also identified. The isolates further shared a core set of 13 chromosomes, but two accessory chromosomes were identified as well. Accessory chromosomes were significantly smaller in size, and one carried pseudogenized copies of two effector genes. Whole-genome alignments further revealed genomic islands of near-zero nucleotide diversity interspersed with islands of high nucleotide diversity that co-localized with repeat-rich regions. These regions were likely generated by RIP, which generally asymmetrically affected the genome of *C. fulvum*.

**Conclusions:**

Our results reveal new evolutionary aspects of the *C. fulvum* genome and provide new insights on the importance of genomic structural variations in overcoming host resistance in fungal plant pathogens.

**Supplementary Information:**

The online version contains supplementary material available at 10.1186/s12915-024-01818-z.

## Background

Fungal plant pathogens have a remarkable capacity to evolve rapidly in order to adapt to adverse conditions and overcome host resistance, which poses challenges to the establishment of sustainable strategies for crop protection. The rapid adaptation of fungal pathogens to unfavorable environments is predominantly orchestrated by their genome plasticity, including changes in their genome size, organization, and chromosome number [1]. Genome plasticity is in terms facilitated by the proliferation of transposable elements (TEs), which can comprise up to 90% of the genomic content in some fungal plant pathogens [2–5]. The presence or mobilization of TEs is often further associated with adaptive genomic changes, such as gene deletion [6], gene duplication [7], and horizontal gene transfer [8] that can accelerate genome evolution and create opportunities for overcoming stressful environments. TEs may also trigger single-nucleotide polymorphisms (SNPs) through repeat-induced point (RIP) mutations. RIP is a premeiotic mechanism present in fungal genomes that acts in defense against the deleterious effects of TE proliferation [9–11]. RIP induces transition nucleotide substitutions (C-to-T or the complement G-to-A) in duplicated genomic sequences, with a strong bias toward CpA-to-TpA (or the complement TpG-to-TpA) dinucleotides [10, 12]. In fungi, RIP can occur at every cycle of sexual reproduction, thus resulting in high rates of mutation that can be from the highest among nonviral organisms [13].

Although TEs can, in principle, proliferate almost randomly in a genome, they are often unevenly distributed within fungal genomes, thereby resulting in genomic regions with clustered TEs [14–17]. This architecture is likely instigated by purifying selection against the deleterious effects of TE insertion into more sensitive regions of the genome, such as gene-dense regions [18]. The uneven distribution of TEs compartmentalizes fungal genomes into a bipartite architecture composed of TE-rich, gene-sparse regions and TE-poor, gene-rich regions [19–21]. This bipartite genome architecture is referred to as the “two-speed genome” model of evolution [22, 23] and is often encountered in plant pathogens, as it enables them to overcome host immunity through the fast evolution of their effector and other pathogenicity-related genes [24, 25].

Another component of fungal genomes that contributes to their plasticity and compartmentalization is accessory chromosomes. Also known as “dispensable” or “B chromosomes,” accessory chromosomes are richer in TEs than core chromosomes and are present in only some individuals of a species [26]. Accessory chromosomes are also typically small (< 2 Mb), are not required for basic growth, and exhibit non-Mendelian segregation ratios [27]. In fungi, accessory chromosomes have been reported in many species [17, 28–32], including the wheat pathogen *Zymoseptoria tritici* which has eight accessory chromosomes, the largest number reported thus far for fungi [33, 34]. Even though fungal accessory chromosomes can carry virulence-associated genes [35] and genes involved in the biosynthesis of host-selective toxins [36], their function in most fungal species remains elusive [26], which makes their persistence within fungal populations intriguing.

*Cladosporium fulvum* (Dothideomycetes; Ascomycota; synonyms *Passalora fulva*, *Fulvia fulva*) is a fungal plant pathogen that causes tomato leaf mold [37]. While the disease is currently of notable concern only in certain regions of the world, *C. fulvum* has been used extensively as a model species for studying molecular plant-pathogen interactions [38, 39]. To date, at least 12 effector genes, namely *Avr2*, *Avr4*, *Avr4E*, *Avr5*, *Avr9*, *Ecp1*, *Ecp2*, *Ecp2-2*, *Ecp2-3*, *Ecp4*, *Ecp5*, and *Ecp6*, have been cloned from this pathogen and are shown to be avirulence determinants in tomato accessions with matching *Cf* resistance genes [39]. Of these, *Avr9* was the first fungal avirulence (*Avr*) effector gene to ever be cloned from fungal plant pathogens [40], but its intrinsic function still remains elusive. In tomato, Avr9 is recognized by the cognate Cf-9 resistance protein [41], but isolates of the fungus have emerged that can overcome Cf-9-mediated resistance through loss of *Avr9*, the only mechanism reported in isolates that overcome Cf-9 [42]. Although complete or partial deletion of avirulence effector genes is a common strategy among plant pathogens for overcoming recognition by cognate resistance proteins [42–46], the mechanisms that promote these deletions are often still unknown [43, 45].

The first reference genome for *C. fulvum* isolate Race 0WU was released in 2012 [47]. However, the assembly was highly fragmented because repetitive regions were not properly assembled. Since then, efforts were made to unravel the genome organization of this pathogen [48], and a new chromosome-scale reference genome for *C. fulvum* isolate Race 5 was recently obtained [17]. The new assembly revealed many features of the *C. fulvum* genome that were hidden by the former highly fragmented assembly, including the presence of 13 core and 1 accessory chromosome, and a “checkerboard” genome architecture composed of gene-dense and TE-poor regions interspersed with gene-sparse and TE-rich regions. It also showed that nearly 40% of the genome is affected by RIP mutations, making it one of the fungal species impacted the most by RIP, and laid the foundation to perform chromosome-scale comparative analyses [17].

In this study, we obtained near-complete genome assemblies for four additional isolates of *C. fulvum* that were collected during the 1970s, 1980s, or 1990s from the Netherlands or Poland and, together with the genome of isolate Race 5 that was collected in the Netherlands in 1979 [17], performed chromosome-level comparative analyses among these five genomes. Our findings provide novel insights on the impact of repetitive DNA, RIP, and SVs on effector genes and genome evolution of a fungal plant pathogen.

## Results

### Long-read sequencing of four *C. fulvum* isolates yielded near-complete genome assemblies

**Fig. 1:**
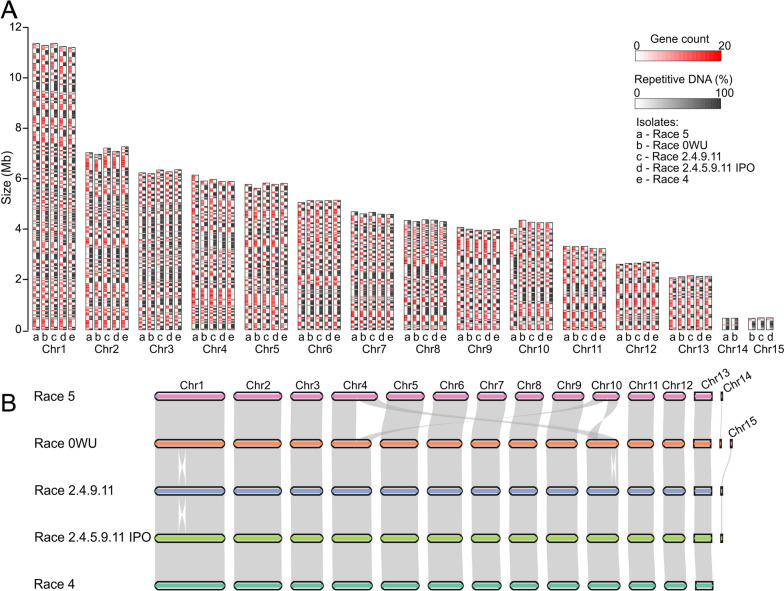
Chromosome-scale genome assemblies of five *Cladosporium fulvum* isolates. **A** Comparison of the size, gene content, and repetitive DNA content among matching chromosomes of *C. fulvum* isolates Race 5, Race 0WU, Race 2.4.9.11, Race 2.4.5.9.11 IPO, and Race 4. Matching chromosomes from different isolates are grouped and depicted as rectangles composed of two tracks representing the gene density (in red) and repetitive DNA content (in black), using a sliding window of 30 kb. **B** Pairwise synteny among the five *C. fulvum* genomes. Ribbons connect syntenic regions of the chromosomes. The figure shows a reciprocal translocation between Chr4 and Chr10 in isolate Race 5, an inversion in Chr10 in isolate Race 0WU, and another inversion in Chr1 in isolate Race 2.4.9.11

Whole-genome sequencing libraries for *C. fulvum* isolates Race 0WU (Netherlands, 1997) [47], Race 4 (Netherlands, 1971) [49], Race 2.4.5.9.11 IPO (Netherlands, 1980s) [49, 50], and Race 2.4.9.11 (Poland, 1980s) [50] were multiplexed into one single SMRT cell and sequenced with the PacBio HiFi technology [51]. The SMRT cell yielded a total of 1,978,275 HiFi high-quality reads with an average size of 10,472 bp (Additional file 1: Fig. S1). After demultiplexing, between 272,759 and 1,031,973 reads per isolate were obtained with an estimated genome coverage of 35 × to 167 × (Additional file 2: Table S1). The reads were next assembled with Canu [52] into representative genomes containing 15 to 18 contigs and ranging from 66.96 to 67.78 Mb in size (Table 1). These genome assemblies are similar in size to the 67.17 Mb genome assembly obtained previously for isolate Race 5 [17]. Using the 14 chromosomes of isolate Race 5 as reference, the genome assemblies of the other four isolates could be further translated into 13 to 15 chromosomes (Fig. 1A). Nearly all assembled chromosomes had the canonical telomeric repeat (TTAGGG)n at both ends, except of Chr13 of isolate Race 4, and Chr5 and Chr12 of isolate Race 2.4.9.11, which were missing telomeric repeats at one chromosome end. However, these three chromosomes had similar sizes compared to their homologous complete chromosomes in other isolates (Additional file 2: Table S2). The genomes of isolates Race 0WU and Race 4 had two unplaced small contigs shorter than 60 kb, whereas the assemblies of the other isolates had no unplaced contigs. Finally, closed circular contigs of 86.6 to 86.8 kb in size were assembled for all four isolates that represented their mitochondrial genomes (Table 1). Collectively, these results indicate that the four *C. fulvum* genomes obtained are nearly complete.

**Table 1:** Genome assembly statistics of five *Cladosporium fulvum* isolates

	**Race 5a**	**Race 0WU**	**Race 4**	**Race 2.4.5.9.11 IPO**	**Race 2.4.9.11**
Assembly size (bp)	67,169,167	67,489,499	66,963,592	67,158,745	67,784,504
GC (%)	48.9	48.9	49.0	48.9	48.9
Number of chromosomes	14	15	13	14	14
Number of unplaced contigs	0	2	2	0	0
Contig N50 (Mb)	5.77	5.62	5.77	5.78	5.82
Contig L50	5	5	5	5	5
mtDNA size (bp)	NA	86,642	86,835	86,771	86,731
Repetitive DNA (%)	50.25	50.15	50.00	49.92	50.47
- Short tandem repeats	0.45	0.46	0.45	0.46	0.46
- Dispersed repeats	49.80	49.69	49.55	49.46	50.01

^a^[17]

### The genomic landscape of repeats is conserved among the isolates of *C. fulvum*

A de novo annotation of repeats in the genomes of the four *C. fulvum* isolates showed that they shared a similar content in repetitive DNA, ranging from 49.9% (33.5 Mb) of the genomic content in isolate Race 2.4.5.9.11 IPO to 50.5% (34.2 Mb) in isolate Race 2.4.9.11 (Table 1). These values are in agreement with the 49.7% (33.4 Mb) of repetitive DNA content reported previously in the genome of isolate Race 5 [17]. The majority of the repeats in the genomes of the isolates were dispersed repeats, which accounted for 99.08% (33.2 Mb) to 99.1% (33.4 Mb) of the repetitive content in the four isolates, whereas short tandem repeats accounted for only 0.90% (0.30 Mb) to 0.92% (0.31 Mb) of the repetitive content. Further annotation of the TEs in the genomes of the four *C. fulvum* isolates produced similar results and showed again only small differences among them (Additional file 1: Fig. S2; Additional file 2: Table S3). As for isolate Race 5, the majority of TEs in the genomes of the four *C. fulvum* isolates were retrotransposons, which accounted for a minimum of 88.5% (30.2 Mb) of the repetitive content in isolate Race 2.4.9.11 to a maximum of 90.3% (30.2 Mb) of the repetitive content in isolate Race 0WU. In contrast, DNA transposons and unclassified repeats ranged from 3.5 to 3.6% and from 5.5 to 7.4% of the repetitive content in the four isolates, respectively. Among retrotransposons, the most common families were the LTR Ty3/mdg-4 family, ranging from 36.5 to 38.3% of the repetitive content in the four isolates, the LINE Tad1 family (29.2 to 31.0%), and the LTR Copia family (18.0 to 20.0%). When compared to the TE content of isolate Race 5, this isolate had less Copia retrotransposons (13.9% of the repetitive content) and more unclassified TEs (12.5% of the repetitive content) compared to the other four isolates (Additional file 1: Fig. S2; Additional file 2: Table S3). However, considering that the genome of isolate Race 5 was assembled using PacBio’s error-prone contiguous long reads (CLR), whereas the genomes of the other four isolates were assembled using PacBio’s HiFi reads, the small differences in TE content could be perhaps explained by the effect of the sequencing technology on the accuracy of assembling repetitive DNA.

### RIP density and distribution patterns are also conserved among the isolates of *C. fulvum*

It was previously shown that *C. fulvum* exhibits one of the highest rates of RIP among fungi, with 39.2% of the genome of isolate Race 5 affected by RIP mutations [17]. Genome-wide RIP analyses using a sliding-window approach bolstered these results by showing that between 40.7 and 41.0% of the genomic content of isolates Race 0WU, Race 4, Race 2.4.9.11, and Race 2.4.5.9.11 IPO were affected by RIP mutations. As expected, between 95.3 and 96.5% of the RIPed regions in the genomes of the four isolates co-localized with repetitive DNA, and no major differences in RIP density and distribution patterns were observed among homologous sets of chromosomes in the five isolates (Additional file 1: Fig. S3; Additional file 2: Table S4). Among the core chromosomes, RIP levels were highest in Chr3, ranging from 52.7 to 53.3% in the five isolates, followed by Chr12 (28.6–30.0%) and Chr13 (28.0–30.0%) (Additional file 2: Table S4). When present, the two accessory chromosomes displayed even higher levels of RIP, ranging between 56.7 and 58.3% in Chr15, and 70.4 to 70.7% in Chr14. Accessory chromosomes also showed higher abundance of RIP leakage toward non-repetitive regions, ranging from 2.82% in Chr15 to 3.69% in Chr14. In contrast, RIP leakage in all core chromosomes was estimated to be less than 0.05%. Genome-wide RIP analyses also revealed the presence of many large RIP-affected regions (LRARs) longer than 4 kb in size, with LRARs numbers ranging from 1492 in isolate Race 4 to 1536 in isolate Race 0WU. Moreover, the average size of LRARs ranged from 16766 bp in isolate Race 0WU to 16991 bp in isolate Race 2.4.9.11, and their average GC content was 42.5%. Finally, given that the isolates exhibited similar patterns of RIP across their chromosomes, they also displayed a similar bimodal distribution in GC content with major peaks at approximately 54% and a minor peak at approximately 42% (Additional file 1: Fig. S4). Collectively, the above results indicate that isolates of *C. fulvum* exhibit limited intraspecific diversity in terms of their genomic landscape of repeats and of RIP patterns, possibly because the fungus reproduces mainly asexually in nature [53].

### A pangenome analysis of the five *C. fulvum* genomes indicates a stable gene content with a low number of accessory genes

The genomes of isolates Race 0WU, Race 4, Race 2.4.9.11, and Race 2.4.5.9.11 IPO were annotated using a combination of ab initio gene predictions and available gene models from *C. fulvum* isolates Race 5 [17] and Race 0WU [47]. We also refined the gene annotation of *C. fulvum* Race 5 and removed 69 transposon-like gene models while adding 372 new gene models, which increased the number of genes in this isolate from 14,690 to 14,993 (Additional file 2: Table S5).

The total number of predicted genes was similar among the five isolates, ranging from 14,895 genes in isolate Race 4 to 14,993 genes in isolate Race 5. A BUSCO-based assessment of the quality and completeness of the gene annotations [54] in the five *C. fulvum* genomes showed that they were 98.8 to 98.9% complete, and that less than 1% of the genes were missing in any of the isolates (Table 2). Further functional annotations showed that the five isolates shared a similar number of protein-coding genes in different functional categories (Additional file 2: Table S6), including categories with relevance to fungal plant pathogens such as CAZymes (519 to 525 genes) (Additional file 1: Fig. S5; Additional file 2: Table S7), proteases (357 to 362 genes) (Additional file 1: Fig. S6; Additional file 2: Table S8), cytochrome P450s (133 to 134 genes) (Additional file 1: Fig. S7A; Additional file 2: Table S9), putative transporters (2277 to 2293 genes) (Additional file 1: Fig. S7B; Additional file 2: Table S10), key enzymes for the biosynthesis of secondary metabolites (SMs) (41 to 42 genes) (Additional file 1: Fig. S7C; Additional file 2: Table S11), secreted proteins (SPs) (1404 to 1425 genes) (Additional file 2: Table S12), and candidate effectors (427 to 440 genes) (Additional file 2: Table S13). Included among the candidate effectors are the previously characterized *Avr2, Avr4*, *Avr4E*, *Avr5*, *Avr9*, *Ecp1*, *Ecp2*, *Ecp2-2*, *Ecp2-3*, *Ecp4*, *E*c*p5*, and *Ecp6* effector genes as well as the additional 67 candidate effectors previously described as extracellular proteins [55]. Similar results were obtained when the protein-coding genes from the five *C. fulvum* isolates were assigned functions based on annotations against the major categories and subcategories of gene ontology (GO, 8047 to 8079 genes) (Additional file 1: Fig. S8A; Additional file 2: Table S14) and the eukaryotic orthologous groups (KOG, 8867 to 8079 genes) (Additional file 1: Fig. S8B; Additional file 2: Table S14).

**Table 2:** Gene prediction statistics for five *Cladosporium fulvum* isolates. BUSCO completeness was estimated based on the Dothideomycetes dataset (*n* = 3786 genes)

	**Race 5**^**a**^	**Race 0WU**	**Race 4**	**Race 2.4.5.9.11 IPO**	**Race 2.4.9.11**
Number of genes	14,993	14,981	14,895	14,944	14,943
Average gene length (bp)	1359	1354	1355	1356	1356
Average exon length (bp)	594	593	594	594	594
Average intron length (bp)	79	79	78	79	79
Average protein length (aa)	422	421	421	421	421
BUSCO completeness					
- Complete	98.9	98.9	98.8	98.8	98.8
- Complete single	98.8	98.7	98.6	98.6	98.5
- Complete duplicated	0.1	0.2	0.2	0.2	0.3
- Complete fragmented	0.3	0.3	0.3	0.3	0.3
- Missing	0.8	0.8	0.9	0.9	0.9

^a^[17]

**Fig. 2:**
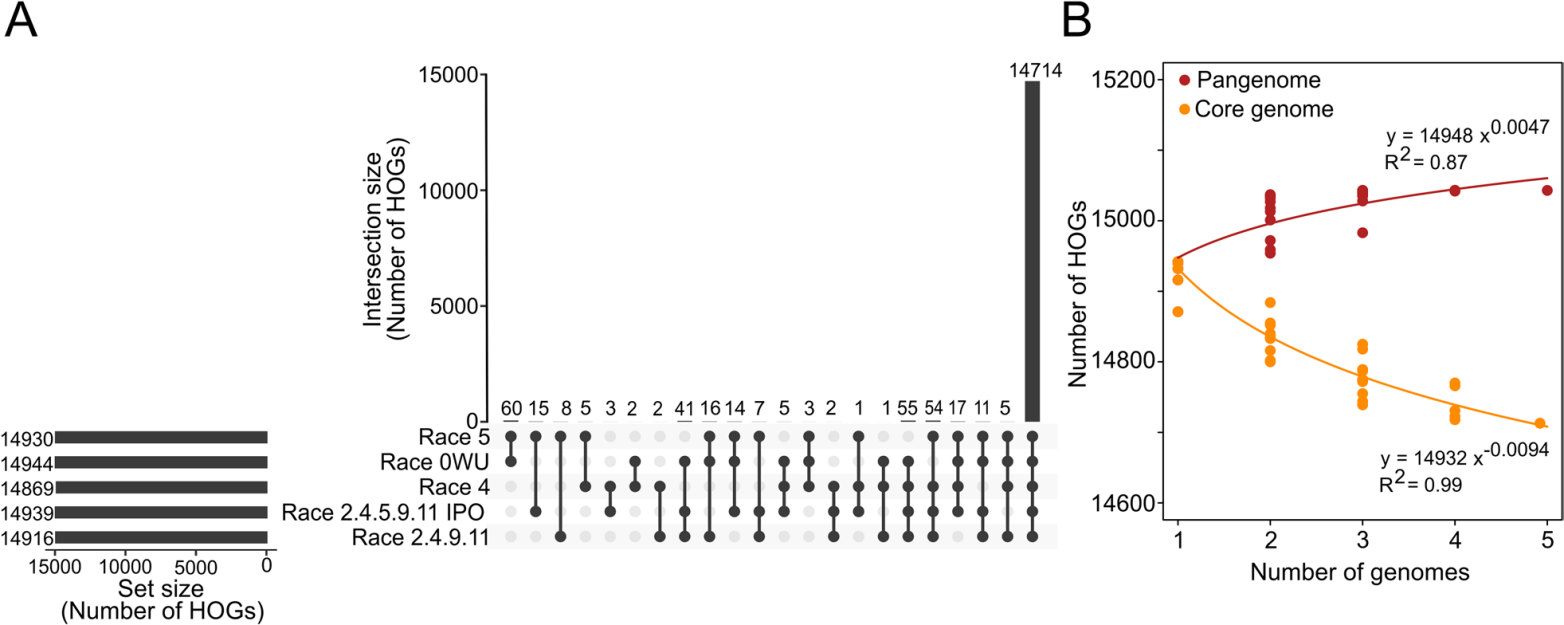
*Cladosporium fulvum* has a low number of accessory genes. **A** Upset plot showing the number of hierarchical orthogroups (HOGs) containing genes from one or more isolates. The figure shows that almost all HOGs are shared by all isolates. **B** Scatterplot showing the estimated sizes of pan- and core genome of *C. fulvum*. The five genomes were sampled in all possible combinations of size *x*, with 1 ≤  ×  ≤ 5. Points represent the number of all HOGs (pangenome) and HOGs containing genes from all sampled genomes (core genome). The curves were fitted by linear regressions of the log-transformed median values of the pan- and core genome. The figure shows that the pangenome size grows slowly as more genomes are included, suggesting that the five sequenced genomes already capture most of the gene space in *C. fulvum*

To further construct a gene-based pangenome for *C. fulvum*, the 74,756 genes that were predicted among the five isolates were organized into hierarchical orthogroups (HOGs) with OrthoFinder. A total of 15,041 HOGs were obtained, which included 99.8 to 99.9% of all predicted genes from each isolate. Nearly all (*n* = 14,962; 99.4%) of these HOGs contained at most one gene per isolate, corresponding to one-to-one orthologs. Surprisingly, all five isolates shared 14,714 HOGs, corresponding to 98.3 to 98.8% of all their genes (Fig. 2A). This indicated that less than 2% of the *C. fulvum* genes were accessory genes. From the 326 HOGs containing accessory genes, 57 contained genes assigned to different functional categories. These include HOGs containing genes encoding CAZymes (*n* = 8), proteases (*n* = 4), cytochrome P450s (*n* = 2), transporters (*n* = 25), key enzymes for biosynthesis of SMs (*n* = 1), secreted proteins (*n* = 22), and candidate effectors (*n* = 12) (Additional file 2: Table S15). However, no significant functional gene category was enriched among the accessory HOGs (hypergeometric test *p*-value < 0.05).

To investigate the extent to which the sizes of the pan- and core genomes of *C. fulvum* changed as a function of the number of genomes analyzed, the five genomes were sampled into subsets of sizes between one and five, and the number of core and accessory HOGs was used as a proxy for the size of pan- and core genome. The size of the pangenome stabilized at 15,043 genes, and although the size of the core genome continued to decrease after including all five isolates, it trended toward stabilizing rapidly (Fig. 2B). These results indicated that *C. fulvum* has a stable gene complement, and that the inclusion of more genomes will not increase considerably the number of novel genes.

### The five genomes of *C. fulvum* exhibit chromosome-scale conservation of synteny and collinearity with few large-scale chromosomal structural variations

A synteny analysis among the five *C. fulvum* genomes indicated that homologous chromosomes shared one-to-one macrosynteny and a large degree of microsynteny and collinearity as well, as both the order and orientation of the genes on them were fairly conserved in the sequenced isolates. Indeed, based on the order of the genes on chromosomes, the number of synteny blocks between any pair of genomes ranged from 15 to 19 and contained between 98.8 to 99.8% of the all the genes in the genomes (Additional file 1: Fig. S9A; Additional file 2: Table S16). Moreover, whole-genome alignments that consider synteny breaks that do not affect the gene order revealed a total of 373 to 1437 synteny blocks that covered between 92.7 and 98.5% of the genomes (Additional file 1: Fig. S9B and Fig. S10; Additional file 2: Table S16).

Overall, only three large-scale chromosomal structural variations (SVs) were observed among the genomes of the five isolates. These SVs corresponded to a reciprocal translocation between chromosomes Chr4 and Chr10 of isolate Race 5 (Fig. 1B) and two large inversions in Chr1 of isolate Race 2.4.9.11 and in Chr10 of isolate Race 0WU, respectively. Mapping of the PacBio reads to the junctions of the reciprocal translocation in isolate Race 5 and the two large inversions in isolates Race 0WU and Race 2.4.9.11 supported their presence and indicated that they were not caused by misassemblies (Additional file 1: Fig. S11). Further analysis of the SVs indicated that the synteny breaks of the reciprocal translocation between Chr4 and Chr10 of isolate Race 5 were localized in repeat-rich regions (Additional file 1: Fig. S12A), thus raising the possibility that the exchange of the chromosome arms was facilitated by the repeats. In addition, although no genes were disrupted by the synteny break points in Chr4, there were two genes flanking the break points. The genes encoded a hypothetical secreted protein of unknown function (CLAFUR5_04694) and the candidate effector Ecp46 (CLAFUR5_12163), indicating that the chromosome arms exchange disrupted the intergenic region of a putative virulence-associated gene. Generally, reciprocal translocations are rarely reported in fungi, but a case has been described in the pine tree pathogen *Dothistroma septosporum*, a close relative of *C. fulvum* [56], in which the translocation occurred between chromosomes Chr5 and Chr13 that are homologous to the *C. fulvum* Chr3 and Chr12, respectively (Additional file 1: Fig. S13). When considering the other two large-scale SVs, the largest of the two was 1.2 Mb long present in Chr1 of isolate Race 2.4.9.11 (Fig. 1B). The synteny break points of this inversion were also in repeat-rich regions and did not disrupt any protein coding sequences (Additional file 1: Fig. S12B). In contrast, synteny breaks of the 654 kb inversion in Chr10 of isolate Race 0WU were in repeat-poor regions and physically close (< 500 bp) to the nearest predicted genes (Additional file 1: Fig. S12C). Interestingly, both synteny breaks of this second largest inversion colocalized with a segment of 7.2 kb that was duplicated in Race 0WU but not in the other four genomes analyzed (Additional file 1: Fig. S12B). The two copies of this duplicated segment were identical and contained three predicted genes, one encoding a hypothetical protein (CLAFUR0_10547), and two encoding two predicted secreted chloroperoxidases (CLAFUR0_10548 and CLAFUR0_10549). Collectively, these results indicate that large-scale SVs in *C. fulvum* often colocalize with repetitive or duplicated DNA, which could either promote or be caused by these large SVs. They further show that large chromosomal rearrangements do not play a significant role in genome evolution of *C. fulvum* but may occasionally affect its infectivity by impacting virulence-associated genes such as effector-encoding genes.

### Loss of the avirulence genes *Avr4E, Avr5,* and *Avr9* is due to SVs induced by transposable elements

**Fig. 3:**
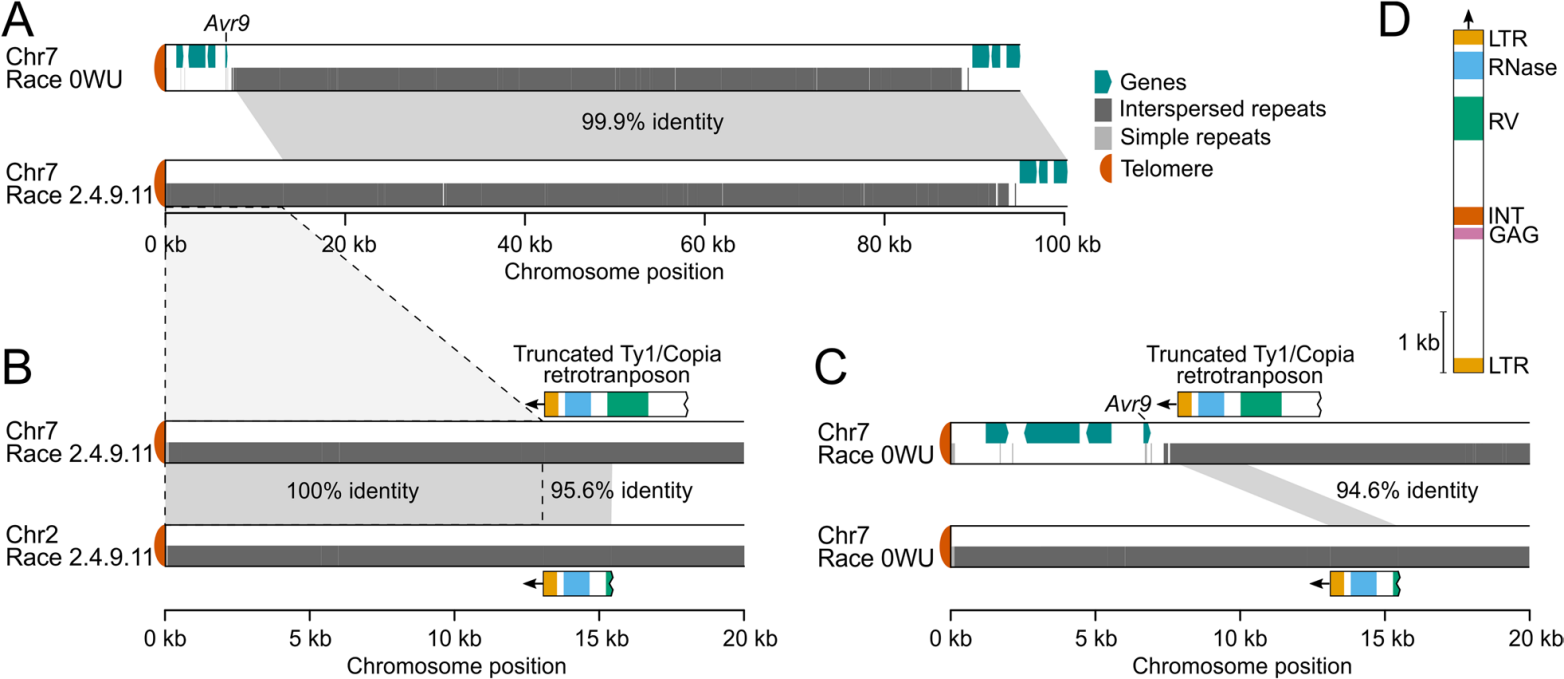
A nonreciprocal translocation between Chr7 and Chr2 causes the deletion of the *Avr9* locus. **A** Alignment of the first 100 kb of Chr7 from isolates Race 0WU which has *Avr9* and Race 2.4.9.11 in which *Avr9* is lost. The 15 kb region that harbors *Avr9* in isolate Race 0WU is absent in isolate Race 2.4.9.11. **B** Alignment of the first 20 kb of Chr7 and Chr2 of isolate Race 2.4.9.11. The left-hand side tip of Chr7 of isolate Race 2.4.9.11 is identical to the sequence of the left-hand side tip of Chr2, and both sequences are flanked by truncated copies of a Ty1/Copia retrotransposon. **C** Alignment of the first 20 kb of Chr7 and Chr2 of isolate Race 0WU. Truncated copies of the same Ty1/Copia retrotransposon are present in the first 15 kb of Chr7 and Chr2 of isolate Race 0WU. **D** Representation of the intact Ty1/Copia retrotransposon shown in **B** and **C**. LTR, long terminal repeat; GAG, group-specific antigen domain; INT, integrase domain; RV, reverse transcriptase domain; RNase, ribonuclease H domain

Effector gene deletion [55, 57] is often reported in *C. fulvum* as a mean to overcome resistance mediated by their cognate resistance genes in tomato, but the mechanisms mediating these deletions remain elusive. We had previously hypothesized that effector gene loss could be a consequence of SVs and the effectors’ physical location in the *C. fulvum* genome [42]. *Avr9*, in particular, whose loss is commonly reported in race 9 strains of the fungus that overcome the Cf-9-mediated resistance in tomato, is situated in a repeat-rich region of the genome that is present at 6.6 kb from the telomeric repeat at the left end of Chr7. This makes *Avr9* putatively prone to deletions [17]. To investigate the mechanism that promotes loss of *Avr9*, Chr7 of isolate Race 2.4.9.11 which lacks *Avr9* [42] was aligned to Chr7 of isolate Race 0WU which has *Avr9*. The alignment revealed that the first 7.8 kb of Chr7 chromosome in isolate Race 0WU is replaced in isolate Race 2.4.9.11 by a 13.1 kb fragment that is largely composed of interspersed repeats and contains no predicted genes (Fig. 3A). Homology searches revealed that the 13.1 kb fragment was nearly identical to the first 13.1 kb of Chr2 of the same isolate (Fig. 3B), suggesting that in isolate Race 2.4.9.11, the first 7.8 kb of Chr7 carrying *Avr9*, was replaced by the first 13.1 kb of Chr2. Interestingly, both copies of the 13.1 kb fragment in Chr2 and Chr7 of isolate Race 2.4.9.11 were flanked on one side by truncated copies of a Ty1/Copia retrotransposon (Fig. 3B). The consensus of this Ty1/Copia retrotransposon was a 5.6 kb sequence flanked by direct repeats of 240 bp long and contained typical domains found in LTR retrotransposons (Fig. 3D). These truncated copies of a Ty1/Copia retrotransposon are also present in other isolates, including Race 0WU (Fig. 3C). Mapping of the HiFi reads from isolate Race 2.4.9.11 to the genome of isolate Race 0WU confirmed the absence of the *Avr9* locus and that its deletion colocalized with the truncated Ty1/Copia copy (Additional file 1: Fig. S14). Collectively, these results support the hypothesis that deletion of *Avr9* in isolate Race 2.4.9.11 was the result of a nonreciprocal translocation between Chr7 and Chr2, promoted by the presence of truncated copies of a Ty1/Copia retrotransposon.

As for *Avr9*, loss of *Avr4E* and *Avr5* is also commonly reported in race 4E and race 5 isolates of the fungus that overcome the cognate *Cf-4E* and *Cf-5* resistance genes, respectively in tomato. To investigate the mechanisms promoting the deletion of *Avr4E* and *Avr5*, the genomes of Race 0WU which has both genes, Race 2.4.5.9.11 IPO which lacks both genes, and/or Race 2.4.9.11 which lacks *Avr4E*, were aligned. *Avr4E* was located within a 8270 bp segment of Chr7 in isolate Race 0WU that was absent in isolates Race 2.4.5.9.11 IPO and Race 2.4.9.11 (Additional file 1: Fig. S15). This segment was flanked by two near-identical copies of a putative DNA transposon Tc1/mariner that was similar (47.1% nucleotide identity) to the Tc1/mariner Molly from the wheat fungal pathogen *Stagonospora nodorum* (AJ488502). A similar organization of repetitive DNA flanking the deletion of the *Avr5* locus in Chr1 was observed in isolate Race 2.4.5.9.11 IPO (Additional file 1: Fig. S16). In this isolate, we noticed the deletion of a long 91,338 bp fragment containing *Avr5* and part of its up- and downstream intergenic regions. The deleted fragment was flanked by two similar (85.9% identity) copies of a putative non-LTR LINE/Tad1 retrotransposon located on the same DNA strand (Additional file 1: Fig. S16).

Collectively, the above results demonstrate that the deletion of *Avr4E*, *Avr5*, and *Avr9* in the genome of race 4E, race 5, and race 9 isolates of *C. fulvum*, respectively, is due to SVs mediated by the presence of neighboring copies of transposable elements, which possibly serve as templates for nonallelic homologous recombination.

### Most SVs in the genome of *C. fulvum* colocalize with TE-rich regions and do not affect genes

**Fig. 4:**
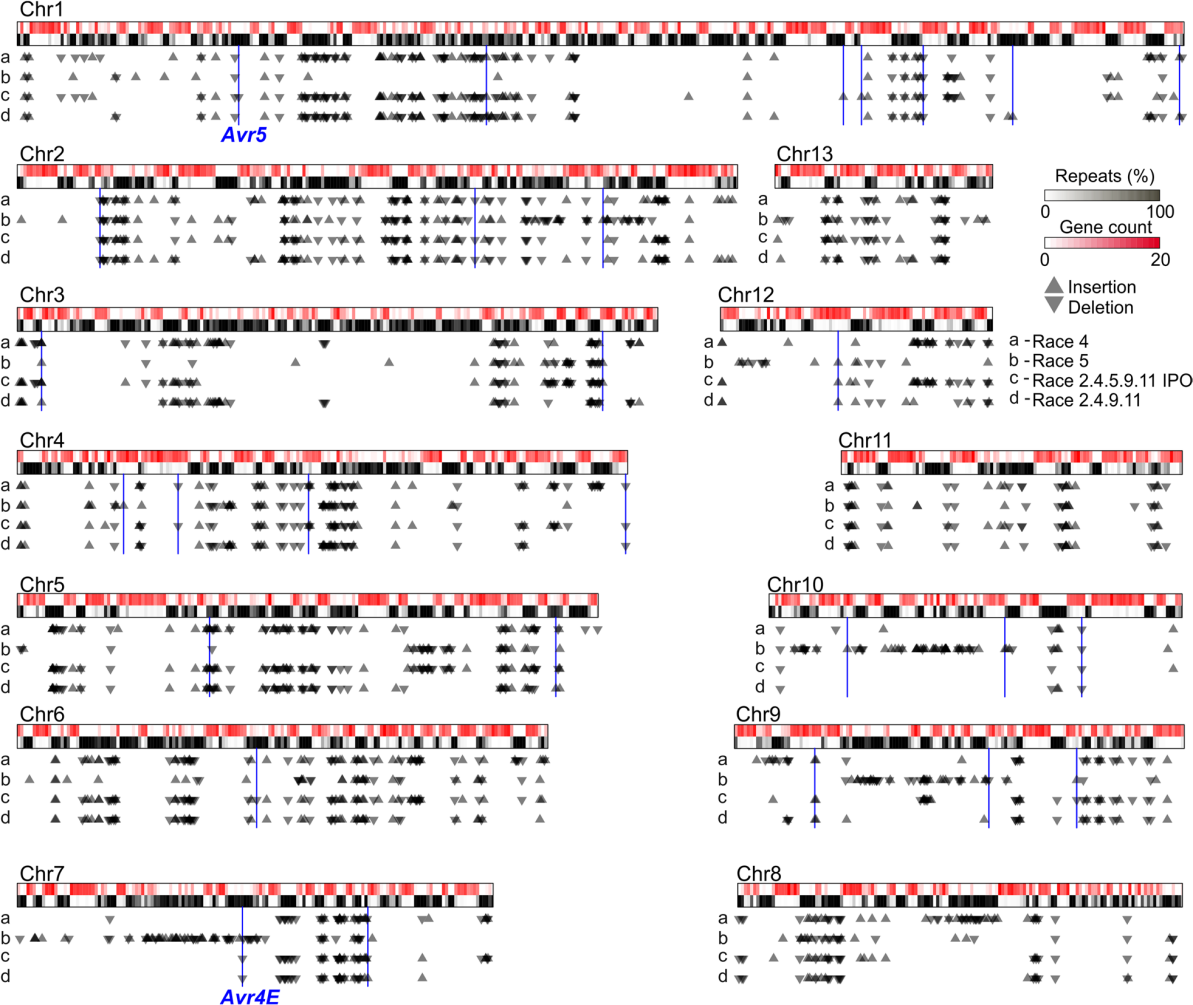
SVs in the genome of *Cladosporium fulvum* are mostly located in repeat-rich regions. The figure shows diagrams of the 13 chromosomes of *C. fulvum* isolate Race 0WU as rectangles with two tracks, representing gene content (top track) and repetitive DNA content (bottom track). Location of SVs (i.e., INDELs longer than 200 bp) is shown as upward (insertion) or downward (deletions) triangles for the four isolates compared to isolate Race 0WU. SVs that affect predicted genes are highlighted with vertical blue lines, and as shown by the figure, overall SVs tend to not affect protein-coding genes. The SVs that resulted in the deletion of *Avr4E* and *Avr5* are labeled

The identification of SVs that affected avirulence genes indicated that some SVs can serve *C. fulvum* to overcome host resistance. To search for other genes affected by SVs, we performed pairwise whole genome alignments using isolate Race 0WU as reference. The number of SVs identified varied from 718 in the genome of isolate Race 5 to 843 in the genome of isolate Race 4 (Additional file 2: Table S17). From the identified SVs, between 697 and 822 (97% to 98%) were long insertions and deletions, indicating that most SVs in *C. fulvum* corresponded to INDELs. Colocalized INDELs were merged, resulting in a total of 662 insertions and 564 deletions (Fig. 4), which varied in size from 205 to 108,643 bp and averaged 6050 bp in length. To investigate the extent to which these large INDELs colocalized with TEs, their coordinates were compared with masked regions of the genome of isolate Race 0WU. The analysis showed that 593 (89.6%) insertions had their insertion sites located within predicted TEs, and 502 (89.0%) deletions had both their start and end coordinates located within predicted TEs. Moreover, 1184 (96.5%) of the INDELs had more than 95% of their sequences composed of predicted TEs (Additional file 1: Fig. S17). Collectively, these results indicate that the vast majority of SVs colocalize with TE-rich regions in the genome of *C. fulvum*.

Although INDELs were largely associated with TE-rich regions of the genome, their impact on predicted genes was minimal. From the 1226 INDELs identified, only 31 (2.5%) overlapped with gene coding regions (Fig. 4), affecting a total of 46 genes (Additional file 2: Table S18). Of these, 13 genes were located within deletions, and, as expected, *Avr5* and *Avr4E* were among them, along with genes predicted to encode an alkaline phosphatase, a serine/threonine-protein kinase, a transcription factor, and a hypothetical secreted protein (Additional file 2: Table S18). Of the rest 33 genes affected by SVs, 15 were predicted to have been duplicated due to the insertion of duplicated segments, with the largest of these duplicated segments containing a group of nine genes in isolate Race 2.4.9.11 (Additional file 1: Fig. S18B). Finally, nine of the genes affected by SVs were disrupted by an insertion in their coding sequence, including a gene that encoded a candidate effector in isolate Race 0WU (CLAFUR0_01596). Notably, the identified insertion in CLAFUR0_01596 is a tandem duplication in isolate Race 2.4.5.9.11 IPO that duplicated a fragment that contained the CLAFUR0_01596 ortholog in this isolate (CLAFUW4_01596) together with a neighboring gene encoding a putative laccase (CLAFUW4_01597) (Additional file 1: Fig. S18A).

Collectively, these results indicate that most of the SVs present in *C. fulvum* are long INDELs largely composed of TEs, which correspond to presence/absence of TE-rich regions or TE insertion site polymorphisms. Furthermore, a small number of the identified SVs affect predicted genes, thus corroborating with our previous observation that *C. fulvum* has a stable gene complement.

### *Cladosporium fulvum* has at least two accessory chromosomes, one of which carries pseudogenized copies of candidate effector genes

A total of 15 chromosomes were assembled from the five genomes of *C. fulvum*, 13 of which (Chr1-Chr13) were core chromosomes common to all isolates and two represented accessory chromosomes that were selectively present in two (Chr4) and three isolates (Chr15), respectively (Fig. 1A). Both Chr14 and Chr15 were further differentiated from the core chromosomes by their small size and high repetitive DNA content (Additional file 2: Table S2), which are typical features of accessory chromosomes [26, 58]. Pairwise alignments of the two accessory chromosomes showed that, when present, they were highly syntenic among isolates, with aligned segments sharing more than 99.9% of nucleotide identity and a conserved complement of 28 (Chr14) or 40 to 41 (Chr15) genes (Additional file 1: Fig. S19). However, of the 69 genes present collectively in Chr14 and Chr15 of isolate Race 0WU, 67 encoded hypothetical proteins, 1 (CLAFUR0_14817) encoded a protein with a conserved kinesin motor domain (PF00225), and 1 (CLAFUR0_14809) encoded a secreted protein. Moreover, 52 (75.4%) of the genes had no homolog in the NCBI nr database based on BLASTp searches (*e*-value < 1E-5) (Additional file 2: Table S19). To investigate whether any of the predicted genes in Chr14 and Chr15 were expressed during host infection, public RNA-seq data of *C. fulvum* Race 0WU-*Solanum lycopersicum* cv. Heinz interaction (NCBI accessions SRR1171035, SRR1171040, SRR1171043) [47] was used to quantify gene expression. From the 69 genes in Chr14 and Chr15, 30 had almost no detectable levels of expression (*TPM* < 3) at any time point (Additional file 2: Table S19). In contrast, six genes in Chr14 and four genes in Chr15, all of which encoded hypothetical proteins and had no BLASTp hits in the NCBI nr database, had considerable levels of expression (*TPM* > 50). These results indicate that most genes in accessory chromosomes are transcriptionally inactive during host infection.

**Fig. 5:**
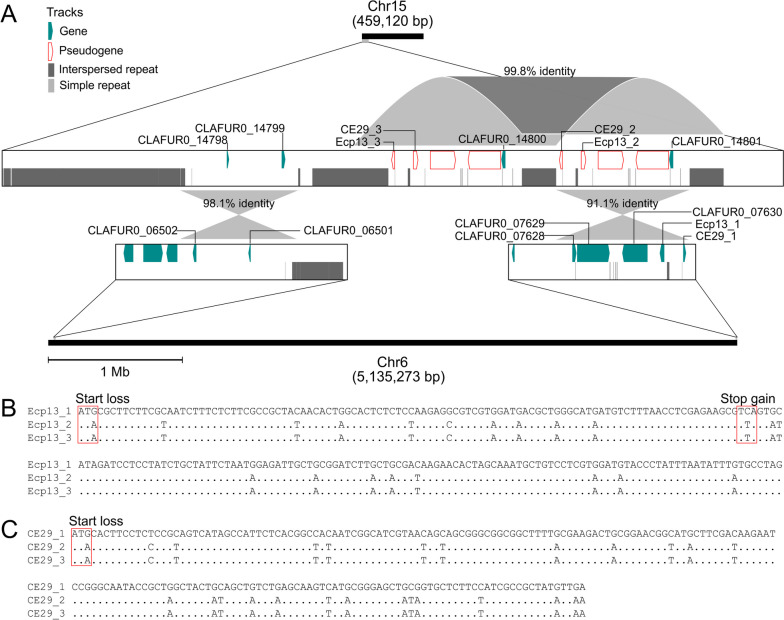
Duplicated segments between a core and a dispensable chromosome of *Cladosporium fulvum* isolate Race 0WU. **A** Intra- and interchromosomal duplications within the first 50 kb of Chr15. A tandem duplication of 12.9 kb fragment is shown. This duplication harbors pseudogenized copies of the candidate effector genes *Ecp13* and *CE29*, for which the functional copies are located 12 kb from the right telomere of Chr6. Underscores followed by numbers were used to distinguish copies of *Ecp13* and *CE29*. The figure also shows another 7.5 kb fragment having one copy in Chr15 and another copy at 40 kb from the left telomere of Chr6. **B** and **C** show the alignments of the coding sequences of *Ecp13* and *CE29* with their pseudogenized copies. Conserved nucleotides are represented by dots. Codons that harbor predicted loss-of-function substitutions are indicated

We have previously demonstrated the presence of gene flow between the core and accessory chromosomes of *C. fulvum*, with a case of a gene (CLAFUR5_14645) in isolate Race 5 that had two identical copies, i.e., one in the core Chr1 and one in the accessory Chr14 [17]. To investigate whether additional genes were shared by core and accessory chromosomes, Chr14 and Chr15 of isolate Race 0WU were hard masked and queried with BLASTn against the 13 core chromosomes (*e*-value < 1E-10). Of the 98,513 bp that were unmasked in Chr14, only 8090 bp (8.2%) had BLAST hits (Additional file 1: Fig. S20). Included was a 934 bp fragment that contained a gene encoding a hypothetical protein with two identical copies in Chr14 (CLAFUR0_14855) and Chr1 (CLAFUR0_00411), respectively, and which was homologous to the CLAFUR5_14645 gene previously reported as duplicated in isolate Race 5 [17]. In a similar way, of the 178,773 bp that were unmasked in Chr15, 45,829 bp (25.6%) had BLAST hits in core chromosomes (Additional file 1: Fig. S20). Included were two fragments of 7.5 kb and 8.6 kb in size, respectively, which were shared by Chr6 and Chr15 (Fig. 5A). These fragments exhibited a peculiar arrangement, as in Chr15 they were situated nearly next to each other, whereas in Chr6 they were present at the opposite ends of this chromosome i.e., at 42 kb from the left-end telomere and at 300 bp from the right-end telomere. Moreover, the 8.6 kb fragment was further tandemly duplicated once in Chr15 (Fig. 5A). Further inspection of the two fragments shared between Chr6 and Chr15 showed that the 7.5 kb long fragment contained two genes of unknown function in Chr6, while the 8.6 kb fragment harbored five genes, of which two encoded hypothetical proteins (CLAFUR0_07628 and CLAFUR0_07629), one encoded a predicted prolyl 4-hydroxylase (CLAFUR0_07630), and two encoded the candidate effectors Ecp13 (CLAFUR0_07631) and CE29 (CLAFUR0_07632), respectively. However, the copies of four of these genes on the 8.6 kb fragment were pseudogenized in Chr15, including the two genes encoding the candidate effectors Ecp13 and CE29. Pseudogenization was caused by the accumulation of C <—> T/G <—> A nucleotide substitutions in their coding sequences, possibly as a result of RIP (Fig. 5B, C). Collectively, these observations suggest the presence of gene flow from a core to an accessory chromosome that involved candidate effectors, followed by pseudogenization of these genes by RIP mutations.

### Repetitive regions are asymmetrically affected by RIP in the genome of *C. fulvum*

**Fig. 6:**
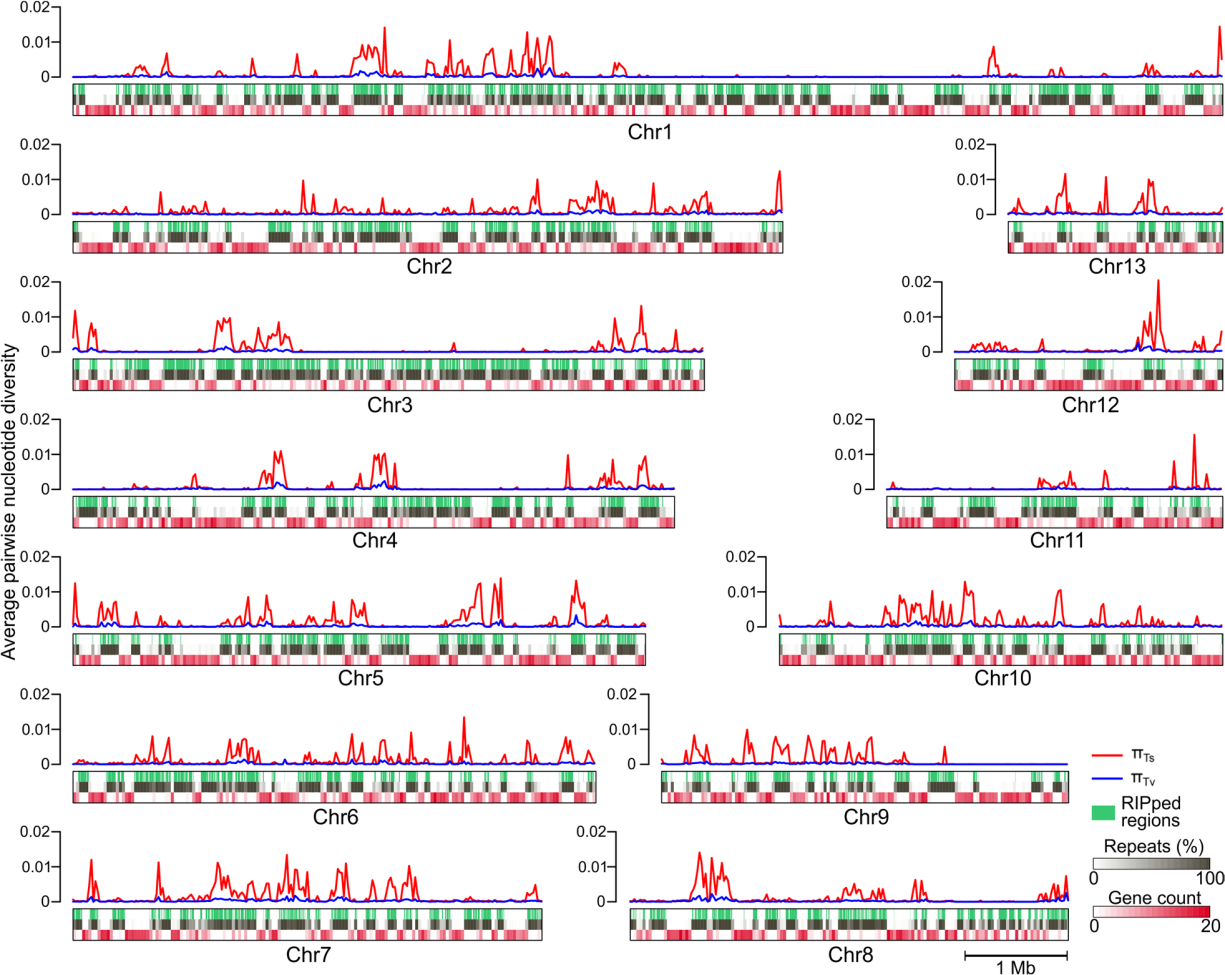
Repetitive regions in the genome of *Cladosporium fulvum* are asymmetrically affected by RIP mutations. Shown are diagrams of the 13 core chromosomes of *C. fulvum* isolate Race 0WU as rectangles with three tracks. From top to bottom, tracks indicate regions affected by RIP (RIPed) (green lines), repetitive DNA content (black lines), and gene content (red lines). The lines on top of the tracks represent average nucleotide diversity values calculated using either transitions (π_Ts_) (red lines) or transversions (π_Tv_) (blue lines) among the complete genomes of five isolates. The figure shows genomic regions of high variability due to transitions in RIPed regions, as well as islands of RIPed regions with almost no variability. Nucleotide diversity was calculated within 20 kb windows

To better understand the genetic and genomic diversity in *C. fulvum*, we performed a whole-genome single-nucleotide polymorphism (SNP) analysis by aligning the genomes of isolates Race 5, Race 4, Race 2.4.9.11, and Race 2.4.5.9.11 IPO on the genome of isolate Race 0WU. A total of 192,279 SNPs were identified, most of which (*n* = 183,160; 95.2%) were in intergenic regions. A total of 8794 SNPs were identified within the 14,714 genes conserved in all five isolates. A phylogenetic tree based on these 8794 SNPs indicated considerable genetic diversity among the five isolates analyzed (Additional file 1: Fig. S21). Interestingly, 90% of the SNPs (*n* = 173,651) could be organized into 2000 clusters of 17 to 93,951 bp in size that accounted for 19% of the genomic content of the 13 core chromosomes (12,659,908 bp). A sliding window analysis along the chromosomes revealed genomic regions with low and high nucleotide diversity, suggesting the presence of SNP hotspots (Fig. 6). Such contrasting patterns of nucleotide diversity were essentially due to nucleotide transitions, as the average nucleotide diversity of transitions per site (π_Ts_) ranged from 0 to 0.2, and the average nucleotide diversity of transversions per site (π_Tv_) ranged from 0 to 0.003. Further mapping of the SNPs on the *C. fulvum* chromosomes revealed that the SNP hotspots co-localized with repetitive regions of the genome that were RIPed (Fig. 6), suggesting that they were formed by RIP mutations. This was further supported by the observation that transition nucleotide substitutions in the SNP hotspots exhibited the typical dinucleotide bias of RIP mutations (i.e., CpA ⇿ TpA) (Additional file 1: Fig. S22). However, while RIP has been reported to induce only transitions, the SNP hotspots across the *C. fulvum* chromosomes also exhibited elevated π_Tv_ (Fig. 6; Additional file 1: Fig. S23), suggesting that RIP can also induce transversion substitutions or that, next to RIP, another mechanism is promoting SNP hotspots in *C. fulvum*. Alternatively, it might also be that RIPed regions are under relaxed selection, which allows for the faster accumulation of random mutations.

Although SNP hotspots co-localized with RIPed regions, conversely, several long chromosome segments were present that contained RIPed regions with low nucleotide diversity (Fig. 6). This suggested that repetitive regions of the *C. fulvum* genome were asymmetrically affected by recent RIP mutations. Further analysis showed no evident correlation between the estimated age of transposon families and their nucleotide diversity (Additional file 1: Fig. S24A), as the estimated diversity of TE families that overlapped with regions of π_Ts_ < 0.005 were not significantly different from the estimated diversity of TE families that overlapped with regions of π_Ts_ > 0.005 (Additional file 1: Fig. S24B). Moreover, there was no evident differences in GC content between TE copies within regions of π_Ts_ > 0.005 as compared to TE copies within regions of π_Ts_ < 0.005 (Additional file 1: Fig. S25).

Taken together, the above observations support the existence of genomic islands that are less likely to accumulate RIP mutations compared to other regions of the genome, and that the occurrence of transition substitutions caused by RIP is associated with higher occurrence of transversion substitutions.

## Discussion

The availability of high-quality genome assemblies can significantly advance our understanding of genome plasticity in fungi and its key role in overcoming host resistance in plant pathogens [59, 60]. In this study, we generated high-quality chromosome-level genome assemblies and gene annotations for four isolates of the tomato pathogen *C. fulvum*, thereby increasing the number of *C. fulvum* isolates with near-complete genome assemblies from one [17] to five and allowing the in depth study of genomic SVs in this pathogen. Our whole-genome alignments indicated high levels of synteny among the five *C. fulvum* genomes but uncovered a few large-scale chromosomal SVs as well, including a balanced reciprocal translocation between Chr4 and Chr10 in isolate Race 5. Such large interchromosomal translocations are often reported in asexual fungal species [56, 61–63] and rarely only in sexually reproducing ones [64] since they could result in improper chromosome matching and nondisjunction during meiosis [65]. Although the functional impact of interchromosomal translocations in fungal genomes remains mostly elusive, they nonetheless have been associated with acquisition of novel gene clusters for SM biosynthesis [62] and adaptation to new hosts by the deletion and recovery of effector-encoding genes [6]. We found no evidence that the reciprocal translocation in isolate Race 5 physically disrupted any protein-coding genes, indicating no gain or loss of fitness by sequence diversification. However, it remains unknown whether this reciprocal translocation impacted the expression of genes that were translocated from one chromosome to another.

Fungal plant pathogens typically tolerate many accessory genes that exhibit presence/absence variation among isolates. For instance, in the cereal pathogens *Claviceps purpurea*, *Z. tritici*, and *Pyrenophora tritici-repentis*, 38%, 45%, and 57% of the genes, respectively, are allegedly accessory [66–68]. These genes contribute to the pathogens’ genome plasticity and are believed to be important for adaptation to novel hosts and adverse environmental conditions. In *C. fulvum*, however, less than 2% of the genes were found to be accessory, indicating a highly stable gene complement among isolates of the fungus. This is likely due to the rare recombination events in *C. fulvum*, as the pathogen reproduces almost exclusively asexually [47, 53]. However, lack of sexual reproduction might not solely explain the low number of accessory genes. This is evidenced in the asexual fungal pathogen *Verticillium dahliae*, which is abundant in genomic rearrangements and lineage-specific genes [14]. Alternatively, it is plausible that the five isolates analyzed in this study may underestimate the population diversity of *C. fulvum* since they all originate from Europe, and that a more extensive sampling that includes isolates from different continents may reveal higher number of accessory genes. An amplified fragment length polymorphism (AFLP)-based multilocus analysis of 67 isolates of *C. fulvum* collected worldwide had shown, for example, that European isolates were significantly genetically differentiated from isolates that were collected in the Americas or Japan [53]. The same study, however, which included four of the isolates in this study (i.e., Race 5, Race 0WU, Race 4, and Race 2.4.5.9.11 IPO), had also shown that the sequenced isolates represent different haplotypes of the fungus and they are phylogenetically distinct [53]. Therefore, it is unlikely that the low number of accessory genes is an artifact of sampling or caused in its entirety by the lack of genetic diversity among the five isolates. Finally, the low number of accessory genes in *C. fulvum* contrasts the assumption that many pathogenicity-related genes in this species, such as carbohydrate-degrading enzymes and genes for SM biosynthesis, are not expressed during infection or are pseudogenized [47]. Assuming that these genes are inactive and no longer contribute to fitness, it is intriguing why they persist among the core genes of the genome.

Despite the low number of accessory genes, our study revealed that *C. fulvum* has an additional accessory chromosome, next to the one reported previously [17]. Interestingly, the two accessory chromosomes were both present only in isolate Race 0WU, suggesting that they are regularly gained or lost in isolates of the fungus. The true origin of fungal accessory chromosomes remains largely elusive, but it is widely accepted that they spawn from core chromosomes following major structural changes such as inversions, translocations, and fissions [58, 69]. In support of this assumption, it has been shown that accessory chromosomes can accumulate gene fragments from core chromosomes. Such fragments can be associated with diverse functions that enable accessory chromosomes to acquire novel functions and thereby promote their persistence in a population [70, 71]. In *C. fulvum*, the duplication of a gene of unknown function between a core and an accessory chromosome has been reported, supporting the existence of gene flow between core and accessory chromosomes [17]. Our current results provided further support for this idea and revealed that the accessory Chr15 of *C. fulvum* carries segments of DNA from subtelomeric regions of the core Chr6, including a fragment with pseudogenized copies of the candidate effectors *Ecp13* and *CE29*. One possibility is that the copies of these two candidate effectors were active when migrated to Chr15, thereby increasing the overall fitness of the pathogen. However, because they were spawn by gene duplications, they were eventually pseudogenized by accumulating RIP mutations. A similar scenario was reported for the candidate effector *Ecp11*, which has three tandem copies in *C. fulvum*, one of which is pseudogenized likely by RIP mutations [17]. Overall, our findings support the hypothesis that accessory chromosomes of *C. fulvum* could be a reservoir of genes that rapidly accumulate mutations induced by the presence of TEs [17].

Repetitive DNA and TEs in fungal genomes are targeted by RIP mutations, which typically materialize between the plasmogamy and karyogamy stages of sexual reproduction [72]. However, although sexual reproduction is thought to be rare in *C. fulvum*, nearly all predicted TEs in its genome exhibit evidence of RIP. Our whole-genome alignments also showed the presence within repetitive regions of islands with high nucleotide diversity, mostly caused by transition substitutions with dinucleotide bias, typical of RIP mutations. Large genomic islands with low or near-zero nucleotide diversity were also present within repetitive regions, but their size makes it unlikely that they were fashioned by typical processes that reduce genetic variation, such as selective sweeps. One possibility is that these highly conserved regions have accumulated considerably less RIP mutations compared to regions of high nucleotide diversity, suggesting that the genome of *C. fulvum* is asymmetrically affected by RIP mutations. This might be the case since the RIP machinery does not mutate all repetitive DNA evenly. For instance, short repeats of less than 400 nucleotides frequently escape RIP mutations [73]. Also, tandem duplications are much more likely to be affected by RIP compared to interspersed duplications [11], while divergent copies of less than 80% nucleotide identity are typically not affected by RIP [74]. Although an attractive hypothesis, we found no evidence that TEs in highly conserved regions of the genome of *C. fulvum* escape RIP due to their short size or high divergence among copies, and thus, the origin of the alternating patterns of high and low nucleotide diversity within repetitive regions remains elusive.

Many fungal pathogens are known for their compartmentalized genome architecture with gene-sparse, TE-rich compartments and gene-dense TE-poor regions. As RIP spillage from the TEs often leads to higher mutation rates in neighboring genes [75], the placement of genes in TE-rich compartments is thought to facilitate their faster evolution [14, 23–25]. Even so, TEs may still accommodate genome evolution by inducing gene loss. This is particularly important for fungal plant pathogens as virulence-associated genes such as effector-encoding genes are often enriched in TE-rich regions [19, 21]. Indeed, TEs have been associated with the loss of the *Avr-Pita* effector in the rice blast fungus *Magnaporthe oryzae* [6], the *Ave1* effector in *Verticillium dahliae* [14], and the candidate effector *Zt_8_609* in *Z. tritici* [76]. Loss of these genes provided an advantage to the pathogens in terms of evading effector-triggered immunity mediated by cognate resistance genes in the host. Similarly, we could show that TEs instigated the loss of the *Av4E*, *Avr5*, and *Avr9* effectors in *C. fulvum* to overcome their matching resistance gene in tomato [47, 77]. The precise mechanism by which TEs induce gene loss is often elusive but it has been connected to nonhomologous recombination [78]. For instance, upon random double-strand breaks induced by ionizing radiation in *Saccharomyces cerevisiae*, chromosome rearrangements, including a nonreciprocal translocation, emerged by homologous recombination between nonallelic Ty1 retrotransposons [79]. The authors of this study suggested that the observed chromosomal aberrations could have occurred during DNA repair via the break-induced replication (BIR) pathway. BIR has been associated with restoration of collapsed replication forks by repairing double-strand DNA breaks through invasion into a homologous template [80, 81]. Nonreciprocal translocations can occur via BIR when resection at a double-strand break exposes TEs that allow recombination with other homologous TEs located at ectopic positions [79, 82]. We found that loss of the *Avr9* locus in *C. fulvum* isolate Race 2.4.9.11 is due to a nonreciprocal translocation between Chr7 and Chr2, possibly mediated by BIR while using the Ty1/Copia copies as substrate for strand invasion. The location of *Avr9* and of the Ty1/Copia copies in close proximity to the telomeres was likely the key contributing factor to this nonreciprocal translocation and the deletion of *Avr9* in isolates under selection pressure by the tomato *Cf-9* resistance gene. Similarly, we revealed that the borders of the deleted segments carrying *Avr4E* and *Avr5* colocalized with homologous copies of TEs that likely served as template for nonallelic homologous recombination, thus resulting in the deletion of the effectors *Avr4E* and *Avr5*. These findings highlight the importance of TEs and of the genome organization for the evolution of fungal pathogens.

## Conclusions

By obtaining four additional near-complete genome assemblies of the tomato pathogen *C. fulvum* and comparing five of them in total, in this study, we provided new insights on the role of repetitive DNA, RIP, and SVs in the evolution of this fungal plant pathogen. Notably, the presence of a Ty1/Copia retroelement likely served as a substrate for a nonreciprocal translocation that resulted in the deletion of the effector gene *Avr9*. Moreover, although nearly all TEs in the genome of *C. fulvum* had footprints of RIP mutations, recent RIP mutations that were variable among isolates appeared to have given rise to genomic islands of high nucleotide variability that increased allelic diversity in nearby genes. Our study also provides evidence of effector gene flow between core and accessory chromosomes that support the hypothesis that accessory chromosomes can gain new functions by acquiring sequences from core chromosomes. Finally, the genomes presented herein are of high value for future comparative genomic analyses and functional studies.

## Methods

### Nucleic acid extraction and sequencing

High-molecular weight (HMW) genomic DNA from *C. fulvum* isolates Race 0WU, Race 4, Race 2.4.5.9.11 IPO, and Race 2.4.9.11 was obtained essentially following the protocol of Jones et al. (2019) [83]. PacBio libraries were multiplexed and sequenced using the HiFi protocol on a Sequel II instrument and one SMRT Cell 8 M. Libraries were prepared and sequenced at the DNA Technologies & Expression Analysis Core Lab of the UC Davis Genome Center.

### Genome assembly

Quality of the sequenced PacBio HiFi reads of *C. fulvum* isolates Race 0WU, Race 4, Race 2.4.5.9.11 IPO, and Race 2.4.9.11 was assessed with FastQC v0.12.1 [84]. Reads were then assembled with Canu v2.2 [52] using parameter *-pacbio-hifi* and *genomeSize = 70 m*. Assembled contigs were identified as chromosomes and properly oriented by pairwise alignments performed with NUCmer from the MUMmer package v4 [85] using the 14 chromosomes of *C. fulvum* Race 5 as reference [17]. Contigs representing the mitochondrial genomes were identified by querying the mitochondrial genes of the fungal pathogen *Erysiphe necator* [86] with BLASTn (*e*-value < 1E-10).

### Repetitive DNA annotation

Repetitive DNA was annotated de novo for each genome. Specifically, repeat libraries of interspersed repeats were obtained with RepeatModeler v2.0.2 [87] using the parameter *-LTRStruct*. Short tandem repeats were identified with the Tandem Repeats Finder v4.09.1 [88]. The interspersed repeat libraries were used by RepeatMasker v4.1.2 in sensitive mode (parameter *-s*) to mask the genomes. Alignments produced by RepeatMasker were used by the script *parseRM.pl* [89] with parameters *–land 50,1*, *–parse*, and *–nrem* to estimate content of repetitive DNA from different classes and families. The script *parseRM.pl* was also used to estimate average divergence of repeat families, which were then used to estimate repeat divergence based on a 20-kb sliding window as described in Zaccaron et al. (2023) [90]. Genomic regions predicted to be affected by repeat-induced point (RIP) mutations were identified with RIPper [91] with default parameters. Specifically, the genomes were analyzed using a 1kb sliding windows with step size of 500 bp. Windows with composite index (TpA/ApT)–((CpA + TpG)/(ApC + GpT)) > 0.01, product index TpA/ApT > 1.1, and substrate index (CpA + TpG)/(ApC + GpT) < 0.75 were considered RIPed. RIPed windows were queried with BLASTn (*e*-value < 1e-20, identity > 50%, query coverage > 20%) against the genome assemblies, and those with a single hit were considered as evidence of RIP leakage toward single-copy regions.

### Gene prediction

Predicted gene models of *C. fulvum* isolates Race 5 [17] and Race 0WU [47] were mapped to the genomes of isolates Race 0WU, Race 4, Race 2.4.5.9.11 IPO, and Race 2.4.9.11 with liftoff v1.6.3 [92]. A round of ab initio predictions was performed with Augustus v3.3.3 [93] trained to predict the genes of *C. fulvum* Race 5 [17]. Mapped gene models with more than 50% overlap with interspersed repeats, detected with the script *coverage* from BEDtools v2.30.0 [94], were removed. The remaining mapped gene models were analyzed interactively using the script *overlap* from BEDtools in the following approach. First, because the gene annotation of isolate Race 5 [17] is overall better compared to the annotation of isolate Race 0WU [47], all mapped gene models from Race 5 were retained. Next, mapped gene models from Race 0WU that did not overlap with mapped gene models from Race 5 were added. Similarly, gene models predicted by Augustus that did not overlap with mapped gene models from Race 5 and Race 0WU were added.

To predict additional genes that could be important for pathogenicity, public RNA-seq data of isolate Race 0WU growing in vitro (SRR1171044 [95]), and from infections of tomato (cv. Heinz) at 4 dpi (SRR1171035 [96]), 8 dpi (SRR1171040 [97]), and 12 dpi (SRR1171043 [98]), were obtained from NCBI [47]. Reads were mapped to the genome of Race 0WU with STAR v2.7.10a [99] with a mapping rate of 94.5%, 0.7%, 3.3%, and 16.8%, respectively. The mapped reads were merged with BAMtools v1.9, and 14,401 transcripts were reconstructed with Stringtie v2.2.1 [100]. The nucleotide sequences of the assembled transcripts were obtained with gffread v0.12.7 [101, 102], and 99699 open reading frames (ORFs) were predicted with ORFfinder v0.4.3 [103, 104] with minimum ORF size of 180 bp and starting with ATG only. These ORFs were mapped back to the reference genome of Race 0WU with GMAP v2021.08.25 [105] to obtain a gff file with their coordinates. BEDtools was used to identify ORFs overlapping with interspersed repeats and already predicted genes in isolate Race 0WU. From the 99699 ORFs, 80842 were removed as they overlapped with repeats or existing gene models. From the remaining 18859 ORFs, 114 had a signal peptide predicted with SignalP6 [106] and further confirmed with DeepLoc v2 [107]. These 114 ORFs were added as new gene models in the annotation of isolate Race 0WU. Finally, the gene models of isolate Race 0WU were mapped to the genomes of isolates Race 5, Race 4, Race 2.4.9.11, and Race 2.4.5.9.11 IPO with liftoff, and mapped genes that did not overlap with existent genes were added. Gene completeness was estimated with BUSCO v5.4.4 [54] in protein mode using the Dothideomycetes_db10 2020–08-05 as reference.

### Functional annotation of genes

Genes encoding candidate effectors were predicted as described in [17]. Briefly, secreted proteins were identified with Signalp5 [108] and were further classified as effectors with EffectorP v2 [109]. Specifically, proteins that were shorter than 250 aa, and had at least 2% of cysteine residues, no transmembrane domains according to DeepTMHMM [110] in the mature protein, and no GPI anchors according to PredGPI [111], were considered as candidate effectors. GO terms were assigned to genes with the PANNZER2 web server [112], using a positive predictive value of at least 0.4. Genes were assigned to KOG categories using eggNOG-mapper v2.1.9 [113]. Genes encoding CAZymes were predicted with the dbCAN2 meta server [114] using HMMdb v11 and the default threshold values for HMMER (*e*-value < 1e-15, coverage > 0.35), DIAMOND (*e*-value < 1e-102), and HMMER (*e*-value < 1e-15, coverage > 0.35). CAZymes from families previously described to contain PCWDEs [115] were considered as PCWDEs. Genes encoding proteases were predicted by querying the proteins with BLASTp (*e*-value < 1E-10) against the MEROPS database v12 [116]. Genes encoding transporters were identified by querying the proteins with BLASTp (*e*-value < 1E-10) against the transporter classification database v2021-06–20 [117]. Genes encoding cytochrome P450s were identified by querying the predicted proteins with the script hmmsearch from HMMER v3.3.2 (*e*-value < 1E-3) using the HMM model for cytochrome P450 (PF00067) obtained from the PFAM website [118]. Cytochrome P450s were classified based on BLASTp searches (*e*-value < 1E-10; identity > 40%; query coverage > 40%) against the Dr. Nelson’s database of curated fungal cytochrome P450s [119]. Genes encoding key enzymes for secondary metabolism were identified with antiSMASH v7 [120].

### Identification and visualization of SVs

To detect large-scale SVs, synteny plots of assembled chromosomes were generated based on pairwise gene homology searches implemented in the MCscan pipeline [121] within the JCVI utilities libraries [122]. Confirmation of the chromosomal variations was obtained by mapping the PacBio reads to the genomes using minimap2 v2.24 [123] with parameters *-ax map-pb* and visualizing the borders of the SVs in IGV v2.16.2 [124]. Dot plots based on pairwise whole-genome alignments were generated with NUCmer from the MUMmer package v4 [85]. To detect small-scale SVs, pairwise whole-genome alignments were generated with minimap2 v2.24 [123] with parameters *-a -x asm5 –cs -r2k*. The alignments were then parsed by SVIM-asm v1.0.3 [125] with parameters *haploid –min_sv_size 50 –max_sv_size 100,000*. Insertions and deletions were extracted and then merged with SURVIVOR v1.0.7 [126] with parameters adjusted to use maximum distance between breaking points of 100 bp, to take the type and orientation of SVs into account, and minimum SV size of 200 bp. Repetitive DNA content of INDELs was estimated by extracting the INDEL sequences from the output of SURVIVOR and masking them with RepeatMasker using the repetitive DNA library of *C. fulvum* isolate 0WU. Genes overlapping with SVs were identified using the script *overlap* of BEDtools v2.30 [94]. Plots showing the impact of SVs on genes were generated by extracting homologous regions between two or more genomes, then aligning them using NUCmer [85] while keeping only the best match of each aligned block, and using R v4.3.1 to plot the aligned blocks, genes, and repetitive DNA. To detect duplications between core and accessory chromosomes, Chr14 and Chr15 from isolate Race 0WU were hard masked using the output of RepeatMasker and the *maskfasta* script from BEDtools v2.30 [94]. The hard-masked sequences were then queried with BLASTn (*e-value* = 1E-10) against the core chromosomes of isolates Race 5, Race 4, Race 2.4.9.11, and Race 2.4.5.9.11 IPO. The script *intersect* from BEDtools was used to detect genes from core chromosomes that overlapped with BLASTn hits. Genes that overlapped with BLASTn hits were considered duplicated between core and accessory chromosomes.

### Gene-based pangenome

Predicted genes were organized into hierarchical orthogroups with OrthoFinder v2.5.3 [127]. Number of shared HOGs were counted and visualized with an UpSet plot [128] using the R package UpSetR v1.4.0 [129]. HOGs containing genes from all five isolates analyzed were considered as the core pangenome. Genes from HOGs not shared by all isolates were considered as accessory genes. The core and pangenome curves were obtained using the linear model function *lm* within R to obtain linear least squares fit of the log_*e*_-transformed sizes of core and pangenome sizes in response to the log_*e*_-transformed sizes of the number of genome combinations.

### Gene expression

RNA-seq reads from an isolate Race 0WU-*Solanum lycopersicum* cv. Heinz interaction at 4 dpi (SRR1171035 [96]), 8 dpi (SRR1171040 [97]), and 12 dpi (SRR1171043 [47, 98], and from isolate Race 0WU grown in potato-dextrose broth (SRR1171044 [47, 95], were mapped to the genome assembly of isolate Race 0WU as described above. Number of paired-end reads mapped to the genes was counted with featureCounts from the subread package v2.0.1 [130]. Transcripts per million (TPM) values were estimated with a custom R script [17, 131].

### Nucleotide diversity across chromosomes

The nucleotide diversity across the chromosomes was calculated based on pairwise whole-genome alignments using isolate Race 0WU as reference. Specifically, the genomes of isolates Race 4, Race 5, Race 2.4.9.11, and Race 2.4.5.9.11 IPO were aligned with the genome of isolate Race 0WU using NUCmer and parameters *–maxmatch*, *-c 100*, *-b 500*, and *-l 50*. Alignments were filtered with *delta-filter* with parameters *-m*, *-i 90*, and *-l 100* and then converted to tabular format with *show-coords* with parameters *-THrd*. The filtered alignments were used by SyRI v1.6.3 [132] to identify polymorphisms. SNPs were extracted with the script *vcfasm* that comes with SyRI and then merged into a single VCF file using BCFtools v1.16 [133]. The VCF file was further converted to a genotype matrix using a custom Unix command. The genotype matrix was split into transitions and transversions using the custom R script *split_tstv.R* [134]. The custom R script *calculate_window_pi.R* [134] was then used to calculate the average nucleotide diversity per site of transitions and transversions using a 20 kb sliding window. Dinucleotide bias in regions of high nucleotide diversity was observed by extracting the nucleotides flanking the point mutation and obtaining a sequence logos using WebLogo [135]. A phylogenetic tree of the isolates was obtained by selecting SNPs within 14713 genes present in all 5 isolates using the script *intersect* from BEDtools v2.30.0 [94]. SNPs were converted to a *fasta* file using the script *phylo* from vcfkit v0.2.9 [136], and a tree was generated with RAxML v8.2.12 [137] with parameters *-m ASC_GTRGAMMA* and *–asc-corr* = *lewis*. Pairwise number of segregating sites was obtained with the script *snp-dists* v0.8.2 [138].

### Supplementary Information

**Additional file 1:** **Fig. S1.** Quality of the sequenced PacBio HiFi reads of five *Cladosporium fulvum* isolates. **Fig. S2.** The genomes of five *Cladosporium fulvum* isolates have similar complements of predicted transposable elements (TEs). **Fig. S3.** The chromosomes of five *Cladosporium fulvum* isolates are heavily affected by Repeat-Induced Point (RIP) mutations. **Fig. S4.** Bimodal GC content distribution of five *Cladosporium fulvum* genomes. **Fig. S5.** Number of genes encoding carbohydrate-active enzymes (CAZymes) in five *Cladosporium fulvum* genomes. **Fig. S6.** Number of genes encoding proteases in five *Cladosporium fulvum* genomes. **Fig. S7.** Number of genes encoding cytochrome P450s, transporters, and key enzymes for secondary metabolite biosynthesis (SM) in five *Cladosporium fulvum* genomes. **Fig. S8.** Number of genes in five *Cladosporium fulvum* genomes assigned to different Gene Ontology (GO) terms and EuKaryotic Ortholog Group (KOG) categories. **Fig. S9.** Overall number of pairwise synteny blocks in pairwise alignments of five *Cladosporium fulvum* genomes. **Fig. S10.** Alignment dot plots showing pairwise syntenic regions among *Cladosporium fulvum* genomes. **Fig. S11.** Confirmation of large-scale structural variations in the *Cladosporium fulvum* genomes. **Fig. S12.** Three large-scale chromosomal structural variations were identified among the five isolates of *Cladosporium fulvum*. **Fig. S13.** Comparison of reciprocal translocation events in *Cladosporium fulvum* and the pine tree pathogen *Dothistroma septosporum*. **Fig. S14.** PacBio HiFi reads mapped to the *Avr9 *locus of *Cladosporium fulvum* support a non-reciprocal translocation. **Fig. S15.** The deletion of *Avr4E* in *Cladosporium fulvum* likely requires neighboring copies of a Tc1/mariner DNA transposon. **Fig. S16.** The deletion of *Avr5* in *Cladosporium fulvum* likely requires neighboring copies of a LINE/Tad1 non-LTR retrotransposon. **Fig. S17.** Most long INDELs in the genome of *Cladosporium fulvum* are composed of repetitive DNA. Scatter plot showing 1226 INDELs as points. **Fig. S18.** Cases of tandem gene duplications in the genome of *Cladosporium fulvum*. **Fig. S19.** Matching dispensable chromosomes present in different isolates of *Cladosporium fulvum* exhibit high nucleotide identity. **Fig. S20.** The left end of the dispensable Chr15 of *Cladosporium fulvum* is composed of segments from core chromosomes. **Fig. S21.** Phylogeny of the sequenced *Cladosporium fulvum* isolates. **Fig. S22.** Dinucleotide bias in regions of high nucleotide diversity in the genome of *Cladosporium fulvum*. **Fig. S23.** Positive correlation between nucleotide diversity of transitions and of transversions in the genome of *Cladosporium fulvum*. **Fig. S24.** No differences of repeat family divergence in regions of high and low nucleotide diversity in the *Cladosporium fulvum* genome. **Fig. S25.** No differences in GC content of transposable elements copies within regions of low and high diversity of transitions in the genome of *Cladosporium fulvum*.

**Additional file 2:** **Table S1.** *Cladosporium fulvum* isolates for which near-complete genome assemblies were obtained using PacBio HiFi sequencing technology. **Table S2.** Comparison of the assembled chromosomes of five *Cladosporium fulvum* isolates. **Table S3.** Summary of estimated abundance of transposable elements in the genomes of five *Cladosporium fulvum* isolates. **Table S4.** Summary of regions of the five *Cladosporium fulvum* genomes affected by Repeat-Induced Point (RIP) mutations. **Table S5.** Update of the gene annotations of *Cladosporium fulvum* isolate Race 5. **Table S6.** Summary of genes from different functional categories and select sub-categories in five *Cladosporium fulvum* genomes. **Table S7.** Genes encoding carbohydrate-active enzymes (CAZymes) in five *Cladosporium fulvum* genomes. **Table S8.** Genes encoding proteases in five *Cladosporium fulvum* genomes. **Table S9.** Genes encoding cytochromes P450 in five *Cladosporium fulvum* genomes. **Table S10.** Genes encoding ABC and MFS transporters in five *Cladosporium fulvum* genomes. **Table S11.** Genes encoding key secondary metabolite enzymes in five *Cladosporium fulvum* genomes. **Table S12.** Genes encoding secreted proteins in *Cladosporium fulvum* genomes. **Table S13.** Genes encoding candidate effector proteins in five *Cladosporium fulvum* genomes. **Table S14.** Number of genes in five *Cladosporium fulvum* genomes assigned to different Gene Ontology (GO) terms and EuKaryotic Ortholog Group (KOG) categories. **Table S15.** Accessory genes from five *Cladosporium fulvum* genomes. **Table S16.** Summary of pairwise synteny blocks in pairwise alignments of five *Cladosporium fulvum* genomes. **Table S17.** Number and type of structural variations (SVs) identified in four *Cladosporium fulvum* genomes. **Table S18.** Structural variations (INDELs) affecting genes in the genome of *Cladosporium fulvum*. **Table S19.** Homologs and expression of predicted genes from the dispensable chromosomes Chr14 and Chr15 of *Cladosporium fulvum*.

## Data Availability

The genome assemblies of *C. fulvum* isolate Race 2.4.5.9.11 IPO and isolate Race 2.4.9.11, which have no unplaced contigs, have been deposited at NCBI under accessions CP121173-CP121187 [139] and CP120815-CP120829 [140], respectively. The genome assemblies of *C. fulvum* isolate Race 0WU and isolate Race 4, which have unplaced contigs, have been deposited at NCBI under accessions JARNMG010000000 [141] and JARJJH010000000 [142], respectively. The SRA accessions for the PacBio HiFi reads for isolate Race 0WU, isolate Race 4, isolate Race 2.4.5.9.11 IPO, and isolate Race 2.4.9.11 are SRR24302839 [143], SRR23862434 [144], SRR24303573 [145], and SRR24303582 [146], respectively. Scripts and code snippets used to generate the results are available at https://github.com/alexzaccaron/2023_cfulv_pangen/ [134]. Supplementary files that include *vcf* files of the structural variations and SNPs, repetitive DNA libraries and annotation, hierarchical orthogroups, expression values of all *C. fulvum* isolate Race 0WU genes during interaction with *Solanum lycopersicum* cv. Heinz, and RIP indices values across the genomes are available at Zenodo (https://zenodo.org/doi/10.5281/zenodo.10019509) [147].

## Abbreviations

AFLP: Amplified fragment length polymorphism
Avr: Avirulence
BIR: Break-induced replication
Chr: Chromosome
HOG: Hierarchical orthogroup
INDEL: Insertion/deletion
RIP: Repeat-induced point
SNP: Single-nucleotide polymorphism
SV: Structural variation
TE: Transposable element
TPM: Transcripts per million
