# Supplementary figures and images for: Analysis of five near-complete genome assemblies of the tomato pathogen Cladosporium fulvum uncovers additional accessory chromosomes and structural variations induced by transposable elements effecting the loss of avirulence genes
Source: BMC Biol. 2024 Jan 29;22:25. doi: 10.1186/s12915-024-01818-z (PMC10823647; doi:10.1186/s12915-024-01818-z)

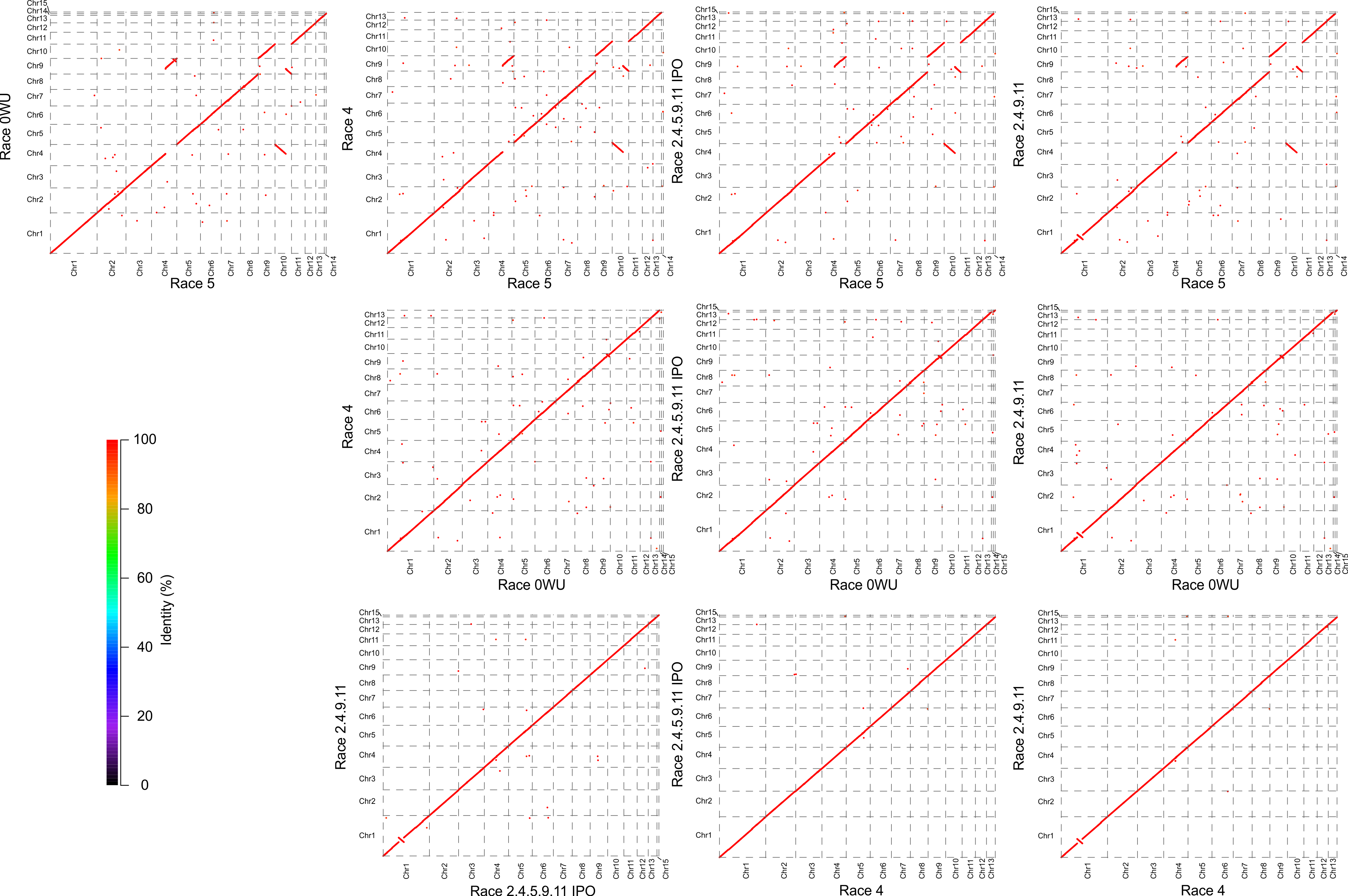

Supplement: Supplementary file 1 — Additional file 1: Fig. S1. Quality of the sequenced PacBio HiFi reads of five Cladosporium fulvum isolates. Fig. S2. The genomes of five Cladosporium fulvum isolates have similar complements of predicted transposable elements (TEs). Fig. S3. The chromosomes of five Cladosporium fulvum isolates are heavily affected by Repeat-Induced Point (RIP) mutations. Fig. S4. Bimodal GC content distribution of five Cladosporium fulvum genomes. Fig. S5. Number of genes encoding carbohydrate-active enzymes (CAZymes) in five Cladosporium fulvum genomes. Fig. S6. Number of genes encoding proteases in five Cladosporium fulvum genomes. Fig. S7. Number of genes encoding cytochrome P450s, transporters, and key enzymes for secondary metabolite biosynthesis (SM) in five Cladosporium fulvum genomes. Fig. S8. Number of genes in five Cladosporium fulvum genomes assigned to different Gene Ontology (GO) terms and EuKaryotic Ortholog Group (KOG) categories. Fig. S9. Overall number of pairwise synteny blocks in pairwise alignments of five Cladosporium fulvum genomes. Fig. S10. Alignment dot plots showing pairwise syntenic regions among Cladosporium fulvum genomes. Fig. S11. Confirmation of large-scale structural variations in the Cladosporium fulvum genomes. Fig. S12. Three large-scale chromosomal structural variations were identified among the five isolates of Cladosporium fulvum. Fig. S13. Comparison of reciprocal translocation events in Cladosporium fulvum and the pine tree pathogen Dothistroma septosporum. Fig. S14. PacBio HiFi reads mapped to the Avr9 locus of Cladosporium fulvum support a non-reciprocal translocation. Fig. S15. The deletion of Avr4E in Cladosporium fulvum likely requires neighboring copies of a Tc1/mariner DNA transposon. Fig. S16. The deletion of Avr5 in Cladosporium fulvum likely requires neighboring copies of a LINE/Tad1 non-LTR retrotransposon. Fig. S17. Most long INDELs in the genome of Cladosporium fulvum are composed of repetitive DNA. Scatter plot showing 1226 IND [file 12915_2024_1818_MOESM1_ESM.zip › Fig_S10B_600 dpi.tif]

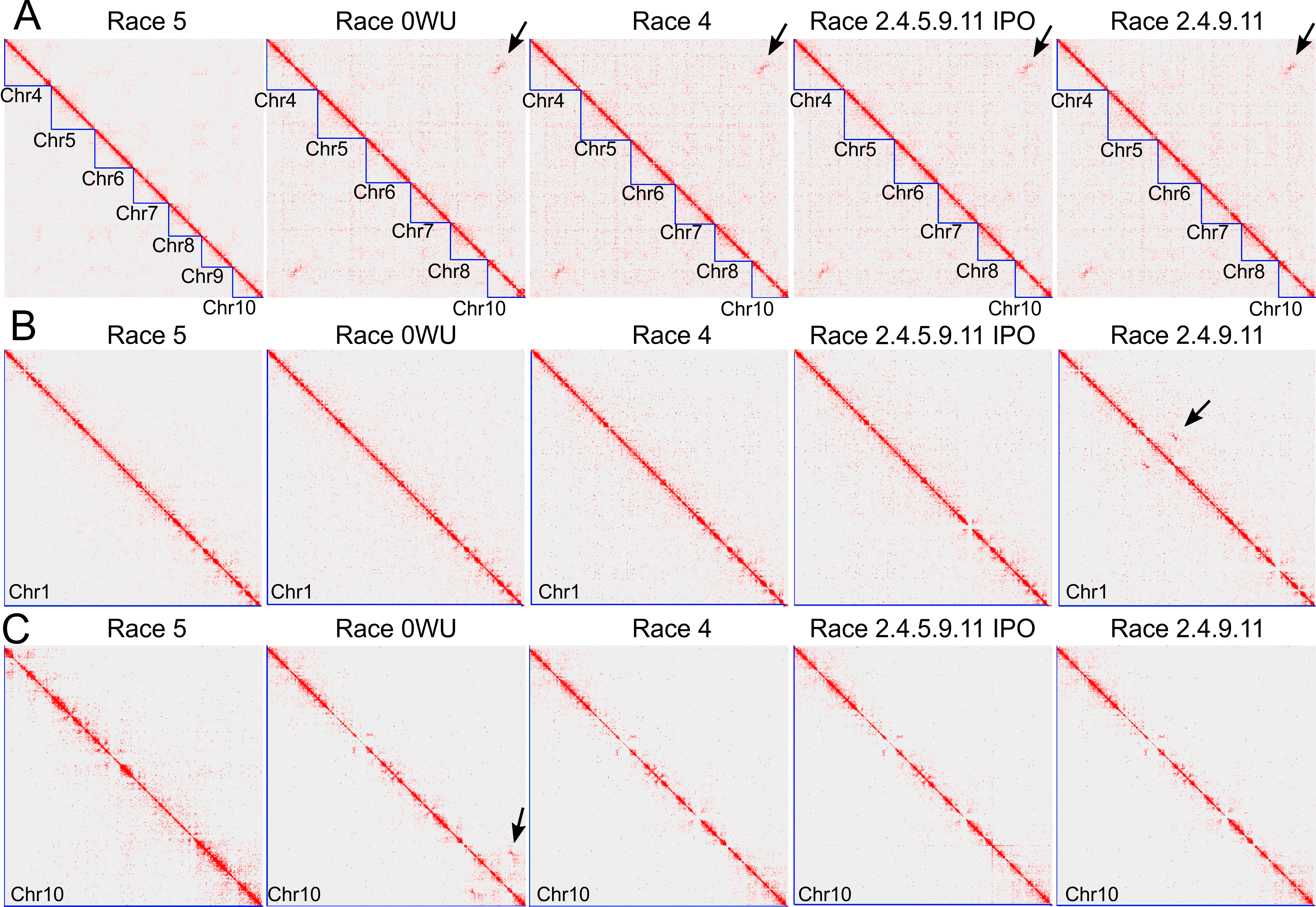

Supplement: Supplementary file 1 — Additional file 1: Fig. S1. Quality of the sequenced PacBio HiFi reads of five Cladosporium fulvum isolates. Fig. S2. The genomes of five Cladosporium fulvum isolates have similar complements of predicted transposable elements (TEs). Fig. S3. The chromosomes of five Cladosporium fulvum isolates are heavily affected by Repeat-Induced Point (RIP) mutations. Fig. S4. Bimodal GC content distribution of five Cladosporium fulvum genomes. Fig. S5. Number of genes encoding carbohydrate-active enzymes (CAZymes) in five Cladosporium fulvum genomes. Fig. S6. Number of genes encoding proteases in five Cladosporium fulvum genomes. Fig. S7. Number of genes encoding cytochrome P450s, transporters, and key enzymes for secondary metabolite biosynthesis (SM) in five Cladosporium fulvum genomes. Fig. S8. Number of genes in five Cladosporium fulvum genomes assigned to different Gene Ontology (GO) terms and EuKaryotic Ortholog Group (KOG) categories. Fig. S9. Overall number of pairwise synteny blocks in pairwise alignments of five Cladosporium fulvum genomes. Fig. S10. Alignment dot plots showing pairwise syntenic regions among Cladosporium fulvum genomes. Fig. S11. Confirmation of large-scale structural variations in the Cladosporium fulvum genomes. Fig. S12. Three large-scale chromosomal structural variations were identified among the five isolates of Cladosporium fulvum. Fig. S13. Comparison of reciprocal translocation events in Cladosporium fulvum and the pine tree pathogen Dothistroma septosporum. Fig. S14. PacBio HiFi reads mapped to the Avr9 locus of Cladosporium fulvum support a non-reciprocal translocation. Fig. S15. The deletion of Avr4E in Cladosporium fulvum likely requires neighboring copies of a Tc1/mariner DNA transposon. Fig. S16. The deletion of Avr5 in Cladosporium fulvum likely requires neighboring copies of a LINE/Tad1 non-LTR retrotransposon. Fig. S17. Most long INDELs in the genome of Cladosporium fulvum are composed of repetitive DNA. Scatter plot showing 1226 IND [file 12915_2024_1818_MOESM1_ESM.zip › Fig_S11B_600 dpi.tif]

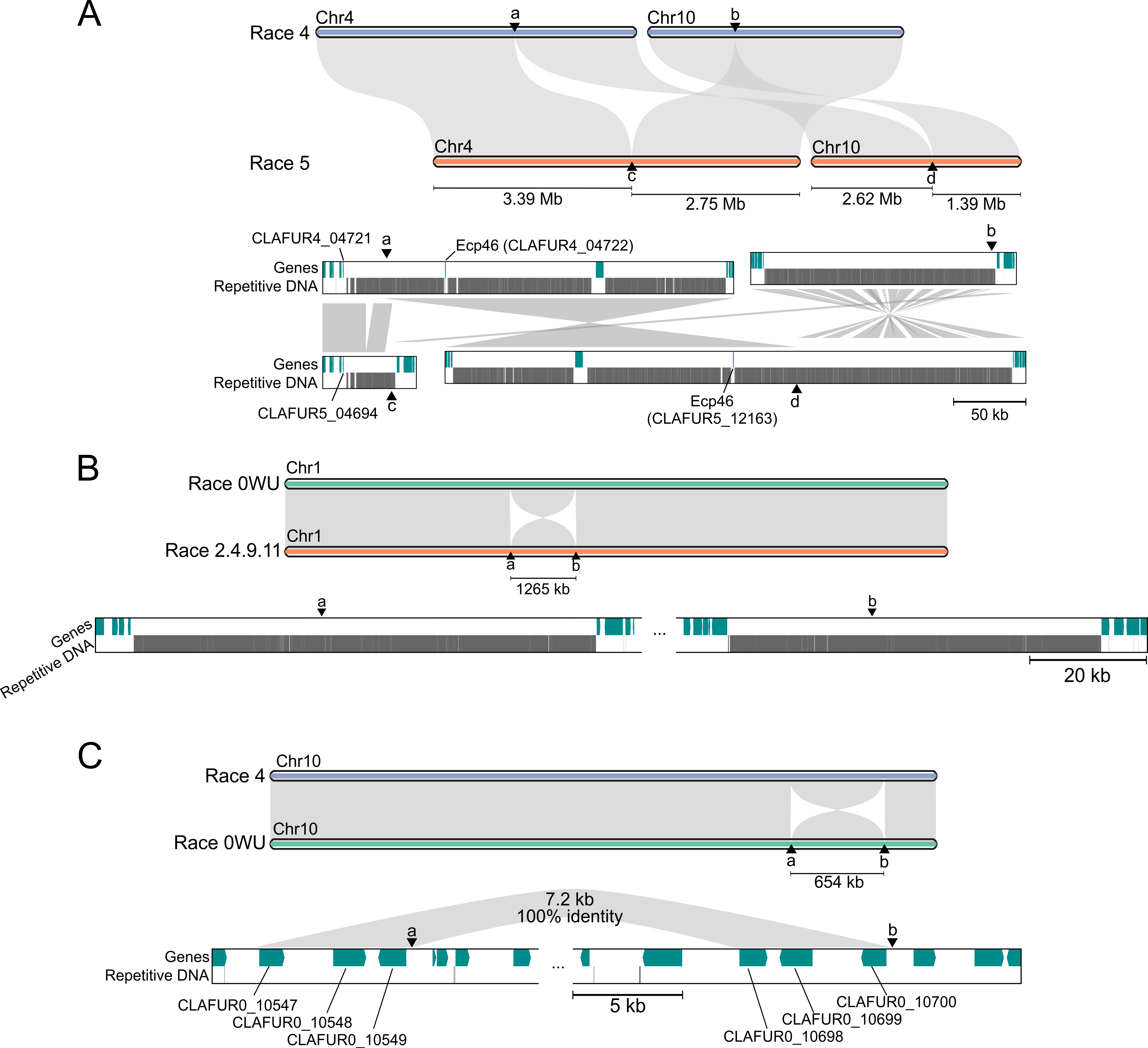

Supplement: Supplementary file 1 — Additional file 1: Fig. S1. Quality of the sequenced PacBio HiFi reads of five Cladosporium fulvum isolates. Fig. S2. The genomes of five Cladosporium fulvum isolates have similar complements of predicted transposable elements (TEs). Fig. S3. The chromosomes of five Cladosporium fulvum isolates are heavily affected by Repeat-Induced Point (RIP) mutations. Fig. S4. Bimodal GC content distribution of five Cladosporium fulvum genomes. Fig. S5. Number of genes encoding carbohydrate-active enzymes (CAZymes) in five Cladosporium fulvum genomes. Fig. S6. Number of genes encoding proteases in five Cladosporium fulvum genomes. Fig. S7. Number of genes encoding cytochrome P450s, transporters, and key enzymes for secondary metabolite biosynthesis (SM) in five Cladosporium fulvum genomes. Fig. S8. Number of genes in five Cladosporium fulvum genomes assigned to different Gene Ontology (GO) terms and EuKaryotic Ortholog Group (KOG) categories. Fig. S9. Overall number of pairwise synteny blocks in pairwise alignments of five Cladosporium fulvum genomes. Fig. S10. Alignment dot plots showing pairwise syntenic regions among Cladosporium fulvum genomes. Fig. S11. Confirmation of large-scale structural variations in the Cladosporium fulvum genomes. Fig. S12. Three large-scale chromosomal structural variations were identified among the five isolates of Cladosporium fulvum. Fig. S13. Comparison of reciprocal translocation events in Cladosporium fulvum and the pine tree pathogen Dothistroma septosporum. Fig. S14. PacBio HiFi reads mapped to the Avr9 locus of Cladosporium fulvum support a non-reciprocal translocation. Fig. S15. The deletion of Avr4E in Cladosporium fulvum likely requires neighboring copies of a Tc1/mariner DNA transposon. Fig. S16. The deletion of Avr5 in Cladosporium fulvum likely requires neighboring copies of a LINE/Tad1 non-LTR retrotransposon. Fig. S17. Most long INDELs in the genome of Cladosporium fulvum are composed of repetitive DNA. Scatter plot showing 1226 IND [file 12915_2024_1818_MOESM1_ESM.zip › Fig_S12B_600 dpi.tif]

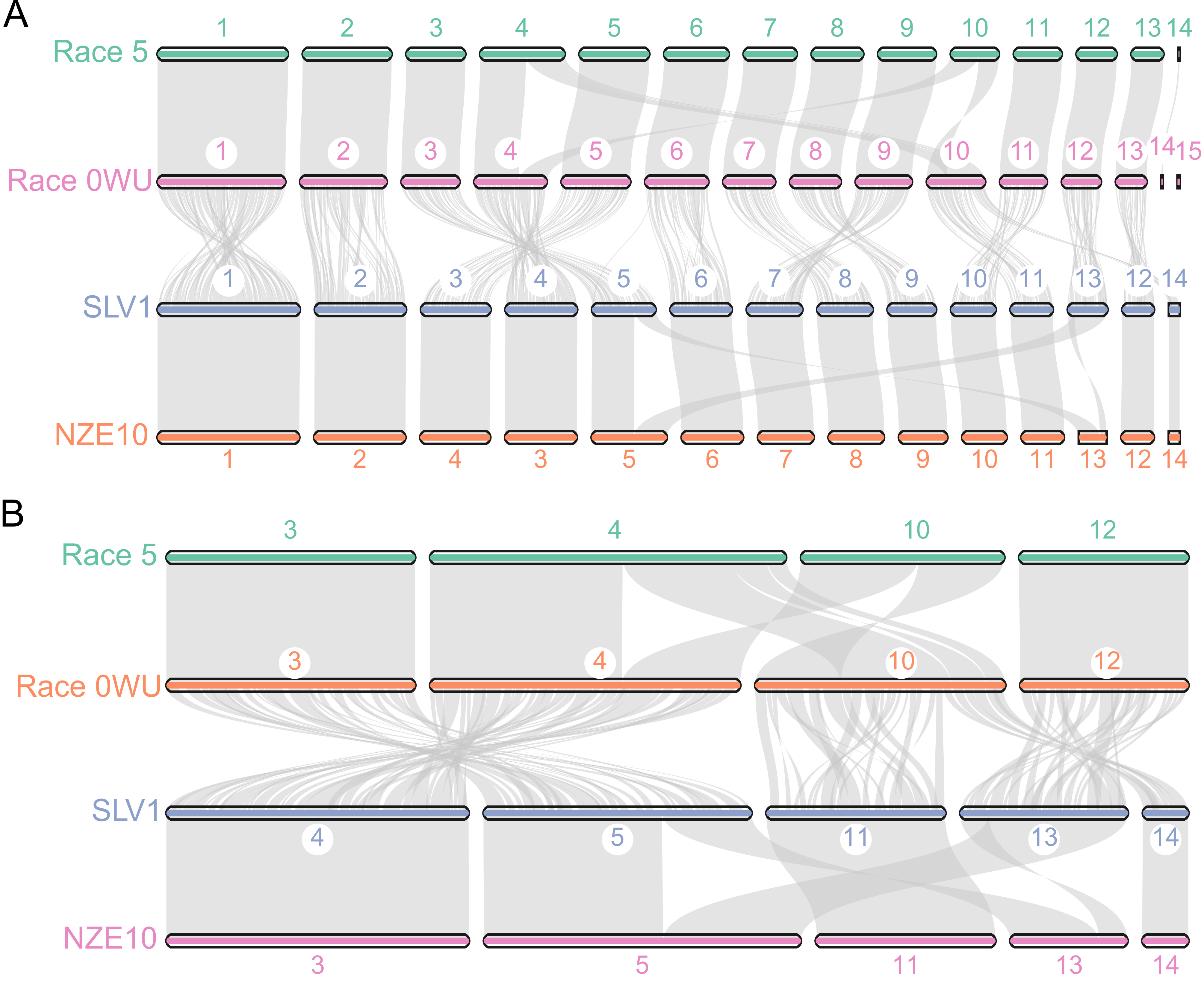

Supplement: Supplementary file 1 — Additional file 1: Fig. S1. Quality of the sequenced PacBio HiFi reads of five Cladosporium fulvum isolates. Fig. S2. The genomes of five Cladosporium fulvum isolates have similar complements of predicted transposable elements (TEs). Fig. S3. The chromosomes of five Cladosporium fulvum isolates are heavily affected by Repeat-Induced Point (RIP) mutations. Fig. S4. Bimodal GC content distribution of five Cladosporium fulvum genomes. Fig. S5. Number of genes encoding carbohydrate-active enzymes (CAZymes) in five Cladosporium fulvum genomes. Fig. S6. Number of genes encoding proteases in five Cladosporium fulvum genomes. Fig. S7. Number of genes encoding cytochrome P450s, transporters, and key enzymes for secondary metabolite biosynthesis (SM) in five Cladosporium fulvum genomes. Fig. S8. Number of genes in five Cladosporium fulvum genomes assigned to different Gene Ontology (GO) terms and EuKaryotic Ortholog Group (KOG) categories. Fig. S9. Overall number of pairwise synteny blocks in pairwise alignments of five Cladosporium fulvum genomes. Fig. S10. Alignment dot plots showing pairwise syntenic regions among Cladosporium fulvum genomes. Fig. S11. Confirmation of large-scale structural variations in the Cladosporium fulvum genomes. Fig. S12. Three large-scale chromosomal structural variations were identified among the five isolates of Cladosporium fulvum. Fig. S13. Comparison of reciprocal translocation events in Cladosporium fulvum and the pine tree pathogen Dothistroma septosporum. Fig. S14. PacBio HiFi reads mapped to the Avr9 locus of Cladosporium fulvum support a non-reciprocal translocation. Fig. S15. The deletion of Avr4E in Cladosporium fulvum likely requires neighboring copies of a Tc1/mariner DNA transposon. Fig. S16. The deletion of Avr5 in Cladosporium fulvum likely requires neighboring copies of a LINE/Tad1 non-LTR retrotransposon. Fig. S17. Most long INDELs in the genome of Cladosporium fulvum are composed of repetitive DNA. Scatter plot showing 1226 IND [file 12915_2024_1818_MOESM1_ESM.zip › Fig_S13B_600 dpi.tif]

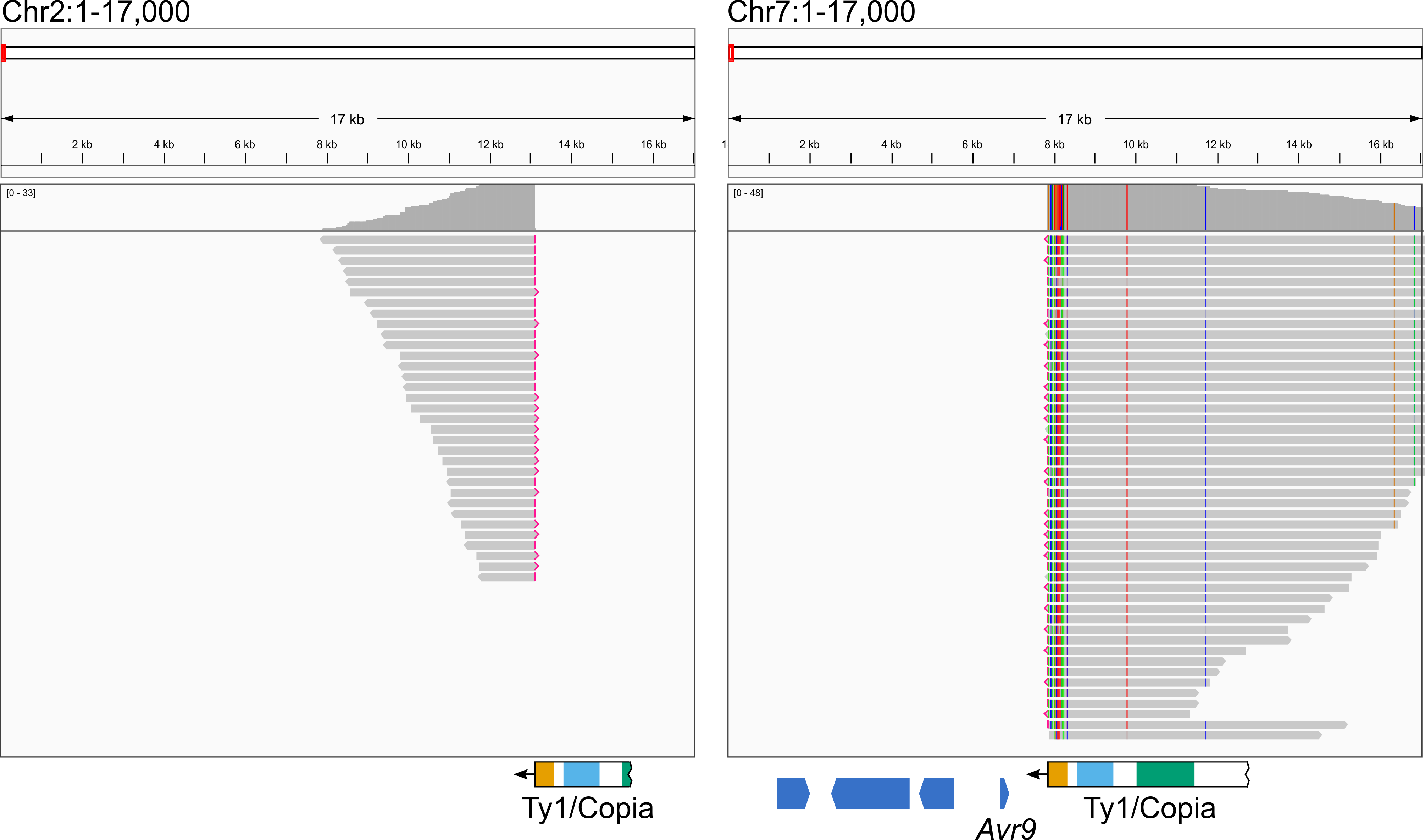

Supplement: Supplementary file 1 — Additional file 1: Fig. S1. Quality of the sequenced PacBio HiFi reads of five Cladosporium fulvum isolates. Fig. S2. The genomes of five Cladosporium fulvum isolates have similar complements of predicted transposable elements (TEs). Fig. S3. The chromosomes of five Cladosporium fulvum isolates are heavily affected by Repeat-Induced Point (RIP) mutations. Fig. S4. Bimodal GC content distribution of five Cladosporium fulvum genomes. Fig. S5. Number of genes encoding carbohydrate-active enzymes (CAZymes) in five Cladosporium fulvum genomes. Fig. S6. Number of genes encoding proteases in five Cladosporium fulvum genomes. Fig. S7. Number of genes encoding cytochrome P450s, transporters, and key enzymes for secondary metabolite biosynthesis (SM) in five Cladosporium fulvum genomes. Fig. S8. Number of genes in five Cladosporium fulvum genomes assigned to different Gene Ontology (GO) terms and EuKaryotic Ortholog Group (KOG) categories. Fig. S9. Overall number of pairwise synteny blocks in pairwise alignments of five Cladosporium fulvum genomes. Fig. S10. Alignment dot plots showing pairwise syntenic regions among Cladosporium fulvum genomes. Fig. S11. Confirmation of large-scale structural variations in the Cladosporium fulvum genomes. Fig. S12. Three large-scale chromosomal structural variations were identified among the five isolates of Cladosporium fulvum. Fig. S13. Comparison of reciprocal translocation events in Cladosporium fulvum and the pine tree pathogen Dothistroma septosporum. Fig. S14. PacBio HiFi reads mapped to the Avr9 locus of Cladosporium fulvum support a non-reciprocal translocation. Fig. S15. The deletion of Avr4E in Cladosporium fulvum likely requires neighboring copies of a Tc1/mariner DNA transposon. Fig. S16. The deletion of Avr5 in Cladosporium fulvum likely requires neighboring copies of a LINE/Tad1 non-LTR retrotransposon. Fig. S17. Most long INDELs in the genome of Cladosporium fulvum are composed of repetitive DNA. Scatter plot showing 1226 IND [file 12915_2024_1818_MOESM1_ESM.zip › Fig_S14B_600 dpi.tif]

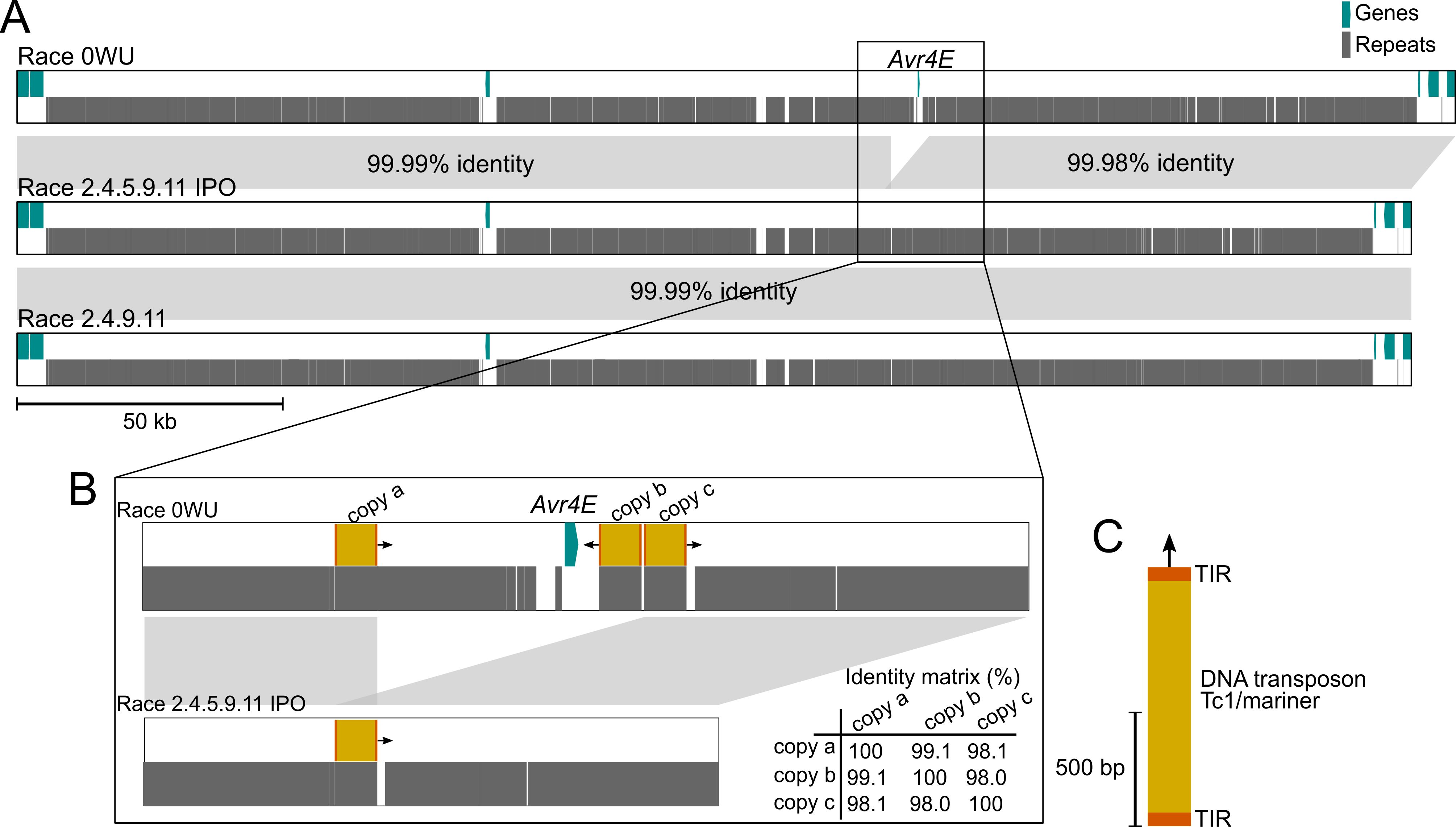

Supplement: Supplementary file 1 — Additional file 1: Fig. S1. Quality of the sequenced PacBio HiFi reads of five Cladosporium fulvum isolates. Fig. S2. The genomes of five Cladosporium fulvum isolates have similar complements of predicted transposable elements (TEs). Fig. S3. The chromosomes of five Cladosporium fulvum isolates are heavily affected by Repeat-Induced Point (RIP) mutations. Fig. S4. Bimodal GC content distribution of five Cladosporium fulvum genomes. Fig. S5. Number of genes encoding carbohydrate-active enzymes (CAZymes) in five Cladosporium fulvum genomes. Fig. S6. Number of genes encoding proteases in five Cladosporium fulvum genomes. Fig. S7. Number of genes encoding cytochrome P450s, transporters, and key enzymes for secondary metabolite biosynthesis (SM) in five Cladosporium fulvum genomes. Fig. S8. Number of genes in five Cladosporium fulvum genomes assigned to different Gene Ontology (GO) terms and EuKaryotic Ortholog Group (KOG) categories. Fig. S9. Overall number of pairwise synteny blocks in pairwise alignments of five Cladosporium fulvum genomes. Fig. S10. Alignment dot plots showing pairwise syntenic regions among Cladosporium fulvum genomes. Fig. S11. Confirmation of large-scale structural variations in the Cladosporium fulvum genomes. Fig. S12. Three large-scale chromosomal structural variations were identified among the five isolates of Cladosporium fulvum. Fig. S13. Comparison of reciprocal translocation events in Cladosporium fulvum and the pine tree pathogen Dothistroma septosporum. Fig. S14. PacBio HiFi reads mapped to the Avr9 locus of Cladosporium fulvum support a non-reciprocal translocation. Fig. S15. The deletion of Avr4E in Cladosporium fulvum likely requires neighboring copies of a Tc1/mariner DNA transposon. Fig. S16. The deletion of Avr5 in Cladosporium fulvum likely requires neighboring copies of a LINE/Tad1 non-LTR retrotransposon. Fig. S17. Most long INDELs in the genome of Cladosporium fulvum are composed of repetitive DNA. Scatter plot showing 1226 IND [file 12915_2024_1818_MOESM1_ESM.zip › Fig_S15B_600 dpi.tif]

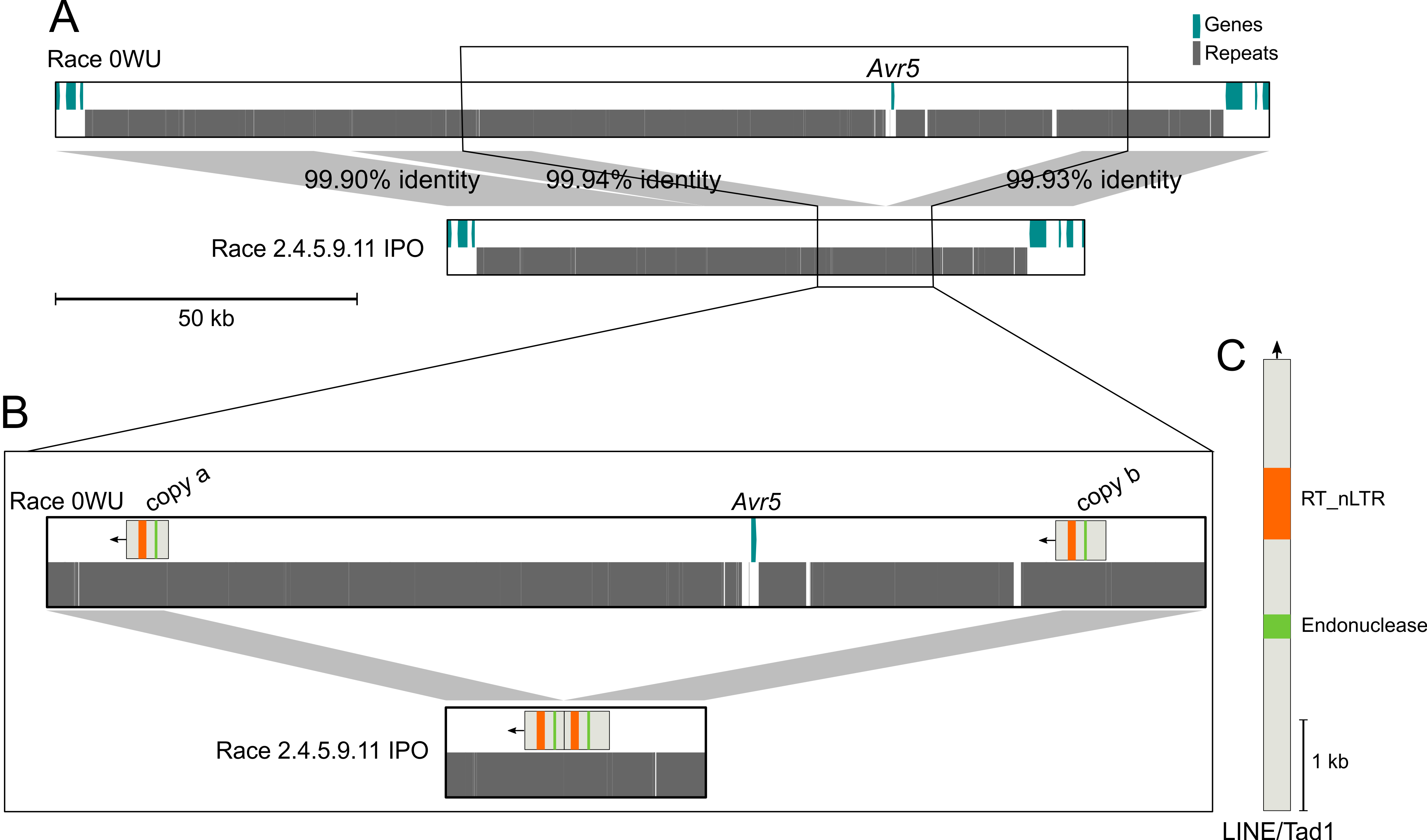

Supplement: Supplementary file 1 — Additional file 1: Fig. S1. Quality of the sequenced PacBio HiFi reads of five Cladosporium fulvum isolates. Fig. S2. The genomes of five Cladosporium fulvum isolates have similar complements of predicted transposable elements (TEs). Fig. S3. The chromosomes of five Cladosporium fulvum isolates are heavily affected by Repeat-Induced Point (RIP) mutations. Fig. S4. Bimodal GC content distribution of five Cladosporium fulvum genomes. Fig. S5. Number of genes encoding carbohydrate-active enzymes (CAZymes) in five Cladosporium fulvum genomes. Fig. S6. Number of genes encoding proteases in five Cladosporium fulvum genomes. Fig. S7. Number of genes encoding cytochrome P450s, transporters, and key enzymes for secondary metabolite biosynthesis (SM) in five Cladosporium fulvum genomes. Fig. S8. Number of genes in five Cladosporium fulvum genomes assigned to different Gene Ontology (GO) terms and EuKaryotic Ortholog Group (KOG) categories. Fig. S9. Overall number of pairwise synteny blocks in pairwise alignments of five Cladosporium fulvum genomes. Fig. S10. Alignment dot plots showing pairwise syntenic regions among Cladosporium fulvum genomes. Fig. S11. Confirmation of large-scale structural variations in the Cladosporium fulvum genomes. Fig. S12. Three large-scale chromosomal structural variations were identified among the five isolates of Cladosporium fulvum. Fig. S13. Comparison of reciprocal translocation events in Cladosporium fulvum and the pine tree pathogen Dothistroma septosporum. Fig. S14. PacBio HiFi reads mapped to the Avr9 locus of Cladosporium fulvum support a non-reciprocal translocation. Fig. S15. The deletion of Avr4E in Cladosporium fulvum likely requires neighboring copies of a Tc1/mariner DNA transposon. Fig. S16. The deletion of Avr5 in Cladosporium fulvum likely requires neighboring copies of a LINE/Tad1 non-LTR retrotransposon. Fig. S17. Most long INDELs in the genome of Cladosporium fulvum are composed of repetitive DNA. Scatter plot showing 1226 IND [file 12915_2024_1818_MOESM1_ESM.zip › Fig_S16B_600 dpi.tif]

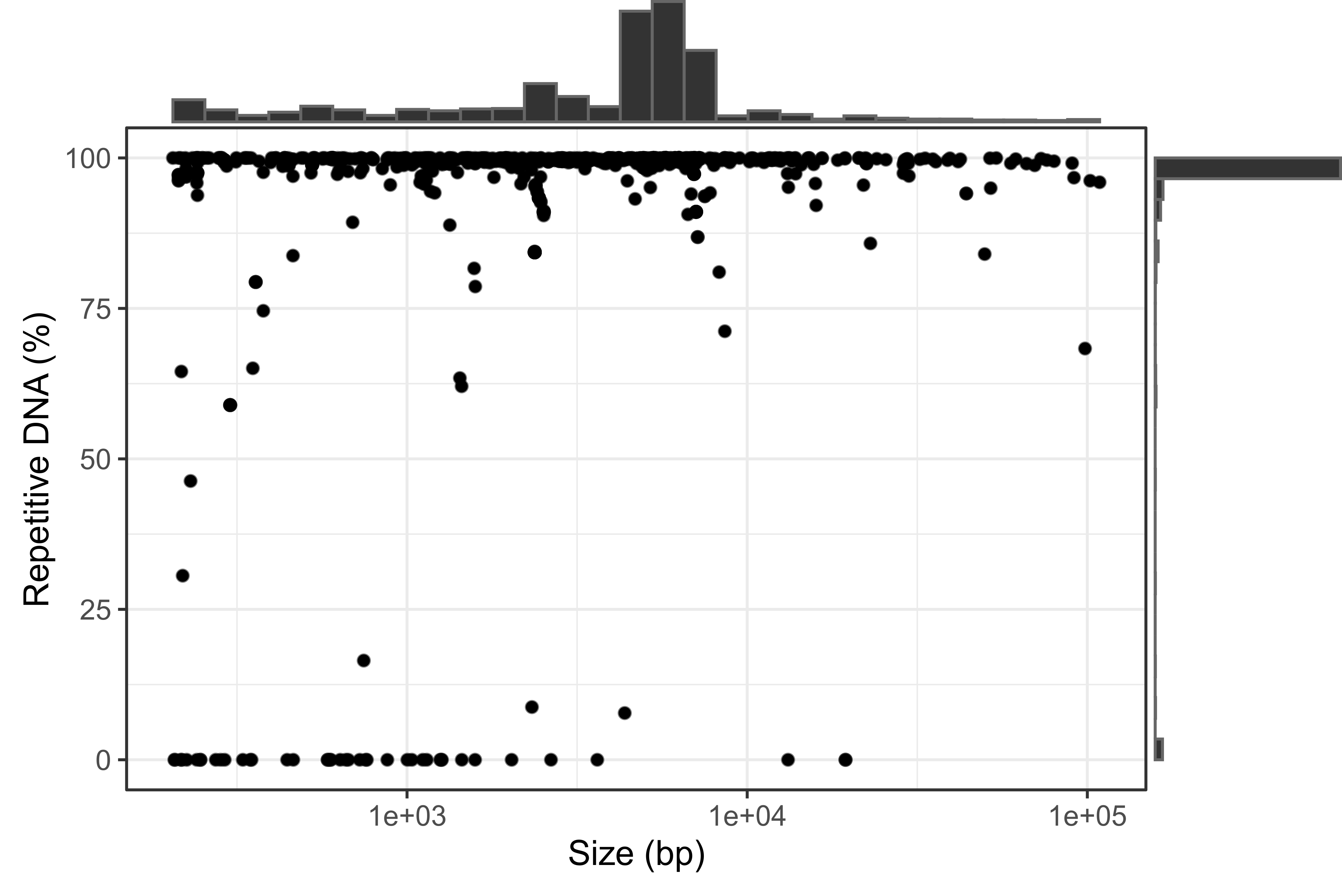

Supplement: Supplementary file 1 — Additional file 1: Fig. S1. Quality of the sequenced PacBio HiFi reads of five Cladosporium fulvum isolates. Fig. S2. The genomes of five Cladosporium fulvum isolates have similar complements of predicted transposable elements (TEs). Fig. S3. The chromosomes of five Cladosporium fulvum isolates are heavily affected by Repeat-Induced Point (RIP) mutations. Fig. S4. Bimodal GC content distribution of five Cladosporium fulvum genomes. Fig. S5. Number of genes encoding carbohydrate-active enzymes (CAZymes) in five Cladosporium fulvum genomes. Fig. S6. Number of genes encoding proteases in five Cladosporium fulvum genomes. Fig. S7. Number of genes encoding cytochrome P450s, transporters, and key enzymes for secondary metabolite biosynthesis (SM) in five Cladosporium fulvum genomes. Fig. S8. Number of genes in five Cladosporium fulvum genomes assigned to different Gene Ontology (GO) terms and EuKaryotic Ortholog Group (KOG) categories. Fig. S9. Overall number of pairwise synteny blocks in pairwise alignments of five Cladosporium fulvum genomes. Fig. S10. Alignment dot plots showing pairwise syntenic regions among Cladosporium fulvum genomes. Fig. S11. Confirmation of large-scale structural variations in the Cladosporium fulvum genomes. Fig. S12. Three large-scale chromosomal structural variations were identified among the five isolates of Cladosporium fulvum. Fig. S13. Comparison of reciprocal translocation events in Cladosporium fulvum and the pine tree pathogen Dothistroma septosporum. Fig. S14. PacBio HiFi reads mapped to the Avr9 locus of Cladosporium fulvum support a non-reciprocal translocation. Fig. S15. The deletion of Avr4E in Cladosporium fulvum likely requires neighboring copies of a Tc1/mariner DNA transposon. Fig. S16. The deletion of Avr5 in Cladosporium fulvum likely requires neighboring copies of a LINE/Tad1 non-LTR retrotransposon. Fig. S17. Most long INDELs in the genome of Cladosporium fulvum are composed of repetitive DNA. Scatter plot showing 1226 IND [file 12915_2024_1818_MOESM1_ESM.zip › Fig_S17B_600 dpi.tif]

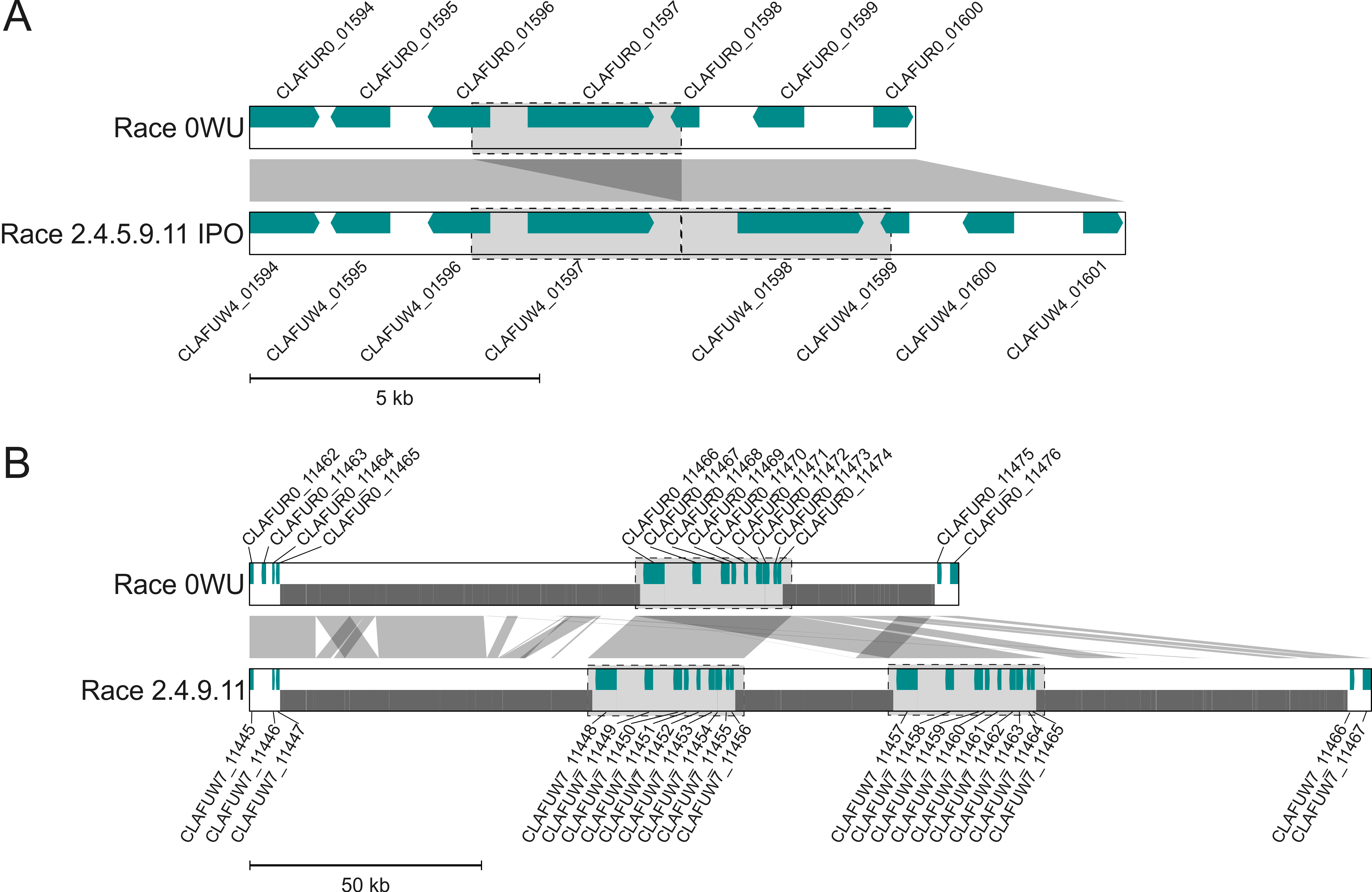

Supplement: Supplementary file 1 — Additional file 1: Fig. S1. Quality of the sequenced PacBio HiFi reads of five Cladosporium fulvum isolates. Fig. S2. The genomes of five Cladosporium fulvum isolates have similar complements of predicted transposable elements (TEs). Fig. S3. The chromosomes of five Cladosporium fulvum isolates are heavily affected by Repeat-Induced Point (RIP) mutations. Fig. S4. Bimodal GC content distribution of five Cladosporium fulvum genomes. Fig. S5. Number of genes encoding carbohydrate-active enzymes (CAZymes) in five Cladosporium fulvum genomes. Fig. S6. Number of genes encoding proteases in five Cladosporium fulvum genomes. Fig. S7. Number of genes encoding cytochrome P450s, transporters, and key enzymes for secondary metabolite biosynthesis (SM) in five Cladosporium fulvum genomes. Fig. S8. Number of genes in five Cladosporium fulvum genomes assigned to different Gene Ontology (GO) terms and EuKaryotic Ortholog Group (KOG) categories. Fig. S9. Overall number of pairwise synteny blocks in pairwise alignments of five Cladosporium fulvum genomes. Fig. S10. Alignment dot plots showing pairwise syntenic regions among Cladosporium fulvum genomes. Fig. S11. Confirmation of large-scale structural variations in the Cladosporium fulvum genomes. Fig. S12. Three large-scale chromosomal structural variations were identified among the five isolates of Cladosporium fulvum. Fig. S13. Comparison of reciprocal translocation events in Cladosporium fulvum and the pine tree pathogen Dothistroma septosporum. Fig. S14. PacBio HiFi reads mapped to the Avr9 locus of Cladosporium fulvum support a non-reciprocal translocation. Fig. S15. The deletion of Avr4E in Cladosporium fulvum likely requires neighboring copies of a Tc1/mariner DNA transposon. Fig. S16. The deletion of Avr5 in Cladosporium fulvum likely requires neighboring copies of a LINE/Tad1 non-LTR retrotransposon. Fig. S17. Most long INDELs in the genome of Cladosporium fulvum are composed of repetitive DNA. Scatter plot showing 1226 IND [file 12915_2024_1818_MOESM1_ESM.zip › Fig_S18B_600 dpi.tif]

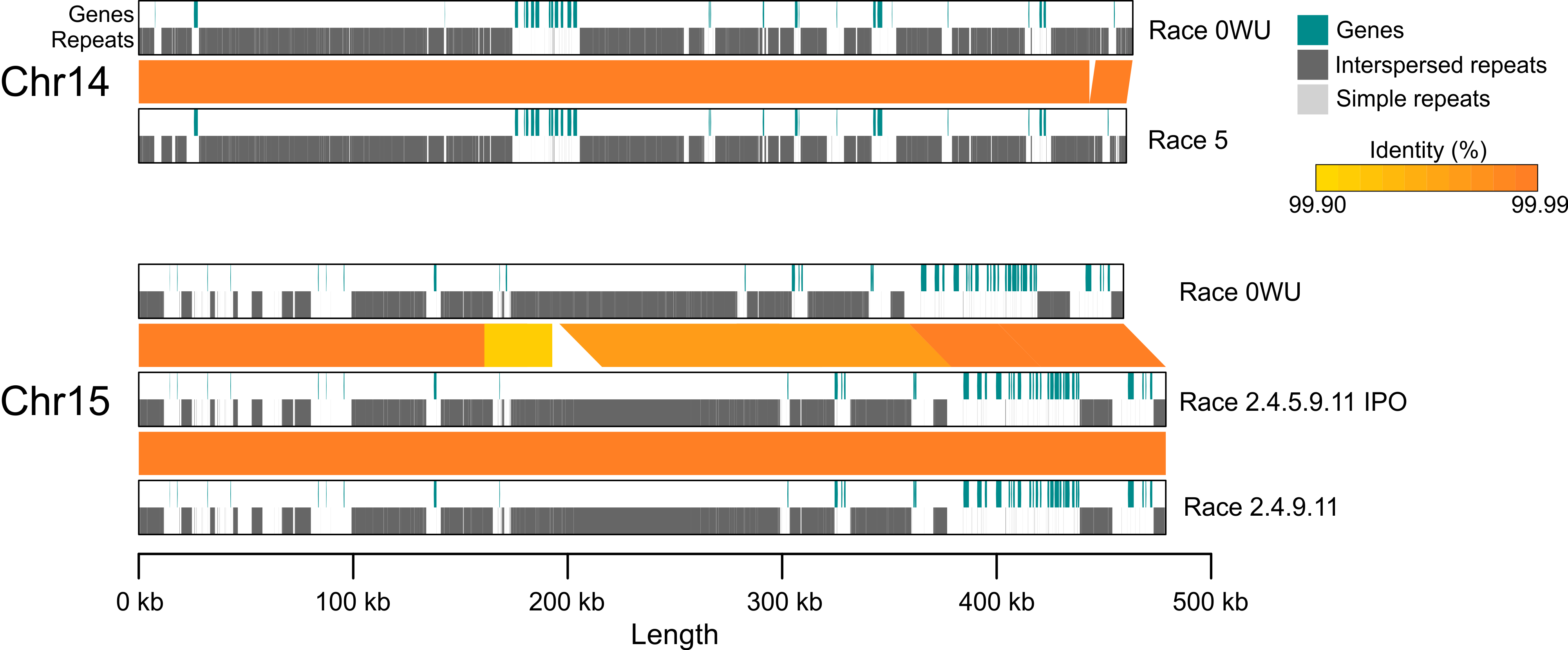

Supplement: Supplementary file 1 — Additional file 1: Fig. S1. Quality of the sequenced PacBio HiFi reads of five Cladosporium fulvum isolates. Fig. S2. The genomes of five Cladosporium fulvum isolates have similar complements of predicted transposable elements (TEs). Fig. S3. The chromosomes of five Cladosporium fulvum isolates are heavily affected by Repeat-Induced Point (RIP) mutations. Fig. S4. Bimodal GC content distribution of five Cladosporium fulvum genomes. Fig. S5. Number of genes encoding carbohydrate-active enzymes (CAZymes) in five Cladosporium fulvum genomes. Fig. S6. Number of genes encoding proteases in five Cladosporium fulvum genomes. Fig. S7. Number of genes encoding cytochrome P450s, transporters, and key enzymes for secondary metabolite biosynthesis (SM) in five Cladosporium fulvum genomes. Fig. S8. Number of genes in five Cladosporium fulvum genomes assigned to different Gene Ontology (GO) terms and EuKaryotic Ortholog Group (KOG) categories. Fig. S9. Overall number of pairwise synteny blocks in pairwise alignments of five Cladosporium fulvum genomes. Fig. S10. Alignment dot plots showing pairwise syntenic regions among Cladosporium fulvum genomes. Fig. S11. Confirmation of large-scale structural variations in the Cladosporium fulvum genomes. Fig. S12. Three large-scale chromosomal structural variations were identified among the five isolates of Cladosporium fulvum. Fig. S13. Comparison of reciprocal translocation events in Cladosporium fulvum and the pine tree pathogen Dothistroma septosporum. Fig. S14. PacBio HiFi reads mapped to the Avr9 locus of Cladosporium fulvum support a non-reciprocal translocation. Fig. S15. The deletion of Avr4E in Cladosporium fulvum likely requires neighboring copies of a Tc1/mariner DNA transposon. Fig. S16. The deletion of Avr5 in Cladosporium fulvum likely requires neighboring copies of a LINE/Tad1 non-LTR retrotransposon. Fig. S17. Most long INDELs in the genome of Cladosporium fulvum are composed of repetitive DNA. Scatter plot showing 1226 IND [file 12915_2024_1818_MOESM1_ESM.zip › Fig_S19B_600 dpi.tif]

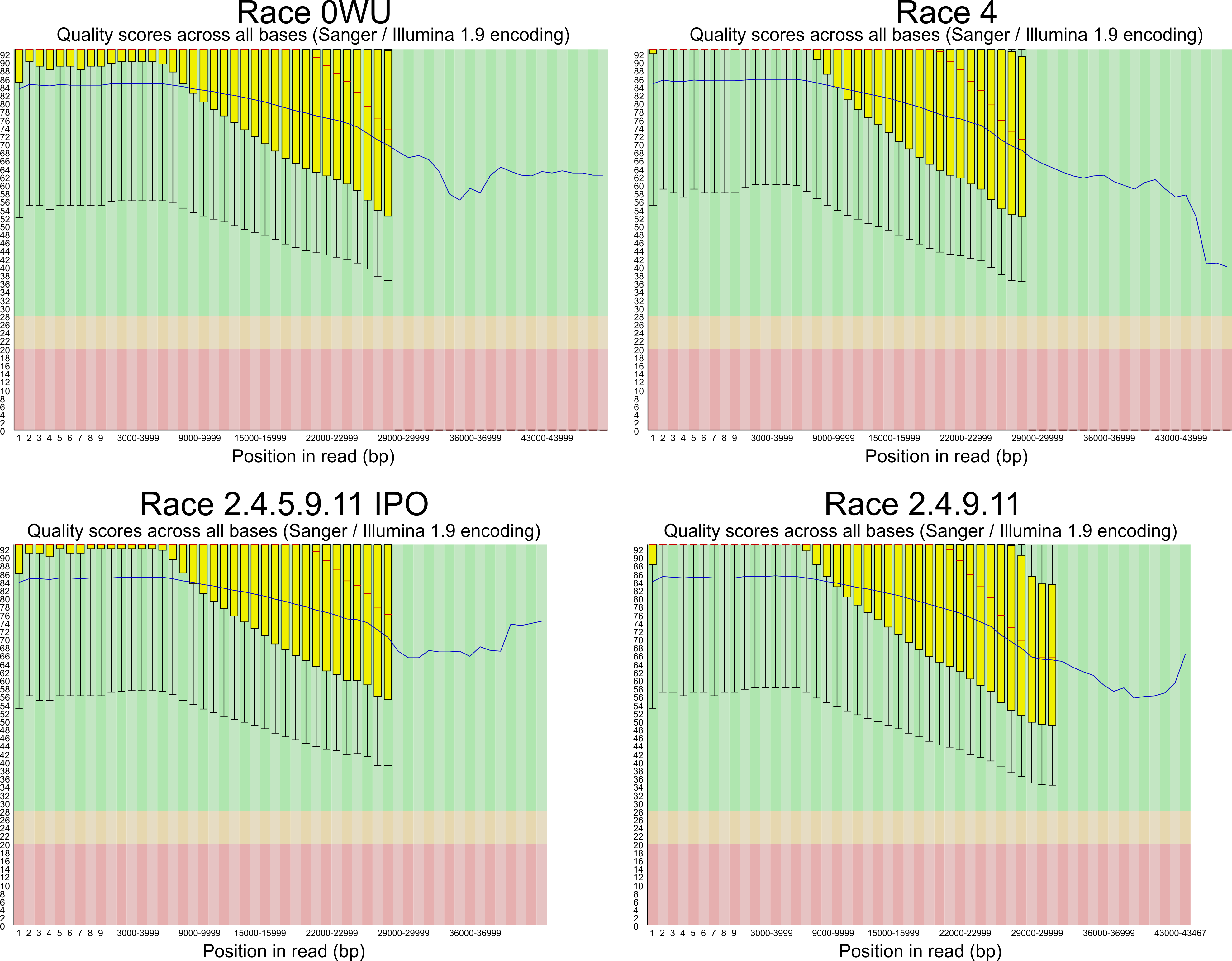

Supplement: Supplementary file 1 — Additional file 1: Fig. S1. Quality of the sequenced PacBio HiFi reads of five Cladosporium fulvum isolates. Fig. S2. The genomes of five Cladosporium fulvum isolates have similar complements of predicted transposable elements (TEs). Fig. S3. The chromosomes of five Cladosporium fulvum isolates are heavily affected by Repeat-Induced Point (RIP) mutations. Fig. S4. Bimodal GC content distribution of five Cladosporium fulvum genomes. Fig. S5. Number of genes encoding carbohydrate-active enzymes (CAZymes) in five Cladosporium fulvum genomes. Fig. S6. Number of genes encoding proteases in five Cladosporium fulvum genomes. Fig. S7. Number of genes encoding cytochrome P450s, transporters, and key enzymes for secondary metabolite biosynthesis (SM) in five Cladosporium fulvum genomes. Fig. S8. Number of genes in five Cladosporium fulvum genomes assigned to different Gene Ontology (GO) terms and EuKaryotic Ortholog Group (KOG) categories. Fig. S9. Overall number of pairwise synteny blocks in pairwise alignments of five Cladosporium fulvum genomes. Fig. S10. Alignment dot plots showing pairwise syntenic regions among Cladosporium fulvum genomes. Fig. S11. Confirmation of large-scale structural variations in the Cladosporium fulvum genomes. Fig. S12. Three large-scale chromosomal structural variations were identified among the five isolates of Cladosporium fulvum. Fig. S13. Comparison of reciprocal translocation events in Cladosporium fulvum and the pine tree pathogen Dothistroma septosporum. Fig. S14. PacBio HiFi reads mapped to the Avr9 locus of Cladosporium fulvum support a non-reciprocal translocation. Fig. S15. The deletion of Avr4E in Cladosporium fulvum likely requires neighboring copies of a Tc1/mariner DNA transposon. Fig. S16. The deletion of Avr5 in Cladosporium fulvum likely requires neighboring copies of a LINE/Tad1 non-LTR retrotransposon. Fig. S17. Most long INDELs in the genome of Cladosporium fulvum are composed of repetitive DNA. Scatter plot showing 1226 IND [file 12915_2024_1818_MOESM1_ESM.zip › Fig_S1B_600 bpi.tif]

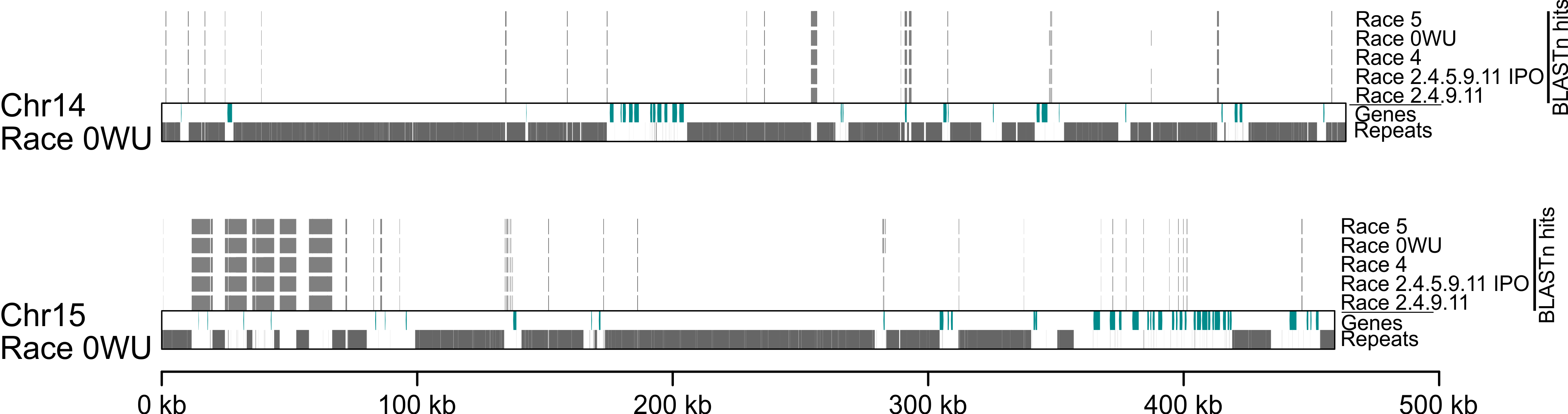

Supplement: Supplementary file 1 — Additional file 1: Fig. S1. Quality of the sequenced PacBio HiFi reads of five Cladosporium fulvum isolates. Fig. S2. The genomes of five Cladosporium fulvum isolates have similar complements of predicted transposable elements (TEs). Fig. S3. The chromosomes of five Cladosporium fulvum isolates are heavily affected by Repeat-Induced Point (RIP) mutations. Fig. S4. Bimodal GC content distribution of five Cladosporium fulvum genomes. Fig. S5. Number of genes encoding carbohydrate-active enzymes (CAZymes) in five Cladosporium fulvum genomes. Fig. S6. Number of genes encoding proteases in five Cladosporium fulvum genomes. Fig. S7. Number of genes encoding cytochrome P450s, transporters, and key enzymes for secondary metabolite biosynthesis (SM) in five Cladosporium fulvum genomes. Fig. S8. Number of genes in five Cladosporium fulvum genomes assigned to different Gene Ontology (GO) terms and EuKaryotic Ortholog Group (KOG) categories. Fig. S9. Overall number of pairwise synteny blocks in pairwise alignments of five Cladosporium fulvum genomes. Fig. S10. Alignment dot plots showing pairwise syntenic regions among Cladosporium fulvum genomes. Fig. S11. Confirmation of large-scale structural variations in the Cladosporium fulvum genomes. Fig. S12. Three large-scale chromosomal structural variations were identified among the five isolates of Cladosporium fulvum. Fig. S13. Comparison of reciprocal translocation events in Cladosporium fulvum and the pine tree pathogen Dothistroma septosporum. Fig. S14. PacBio HiFi reads mapped to the Avr9 locus of Cladosporium fulvum support a non-reciprocal translocation. Fig. S15. The deletion of Avr4E in Cladosporium fulvum likely requires neighboring copies of a Tc1/mariner DNA transposon. Fig. S16. The deletion of Avr5 in Cladosporium fulvum likely requires neighboring copies of a LINE/Tad1 non-LTR retrotransposon. Fig. S17. Most long INDELs in the genome of Cladosporium fulvum are composed of repetitive DNA. Scatter plot showing 1226 IND [file 12915_2024_1818_MOESM1_ESM.zip › Fig_S20B_600 dpi.tif]

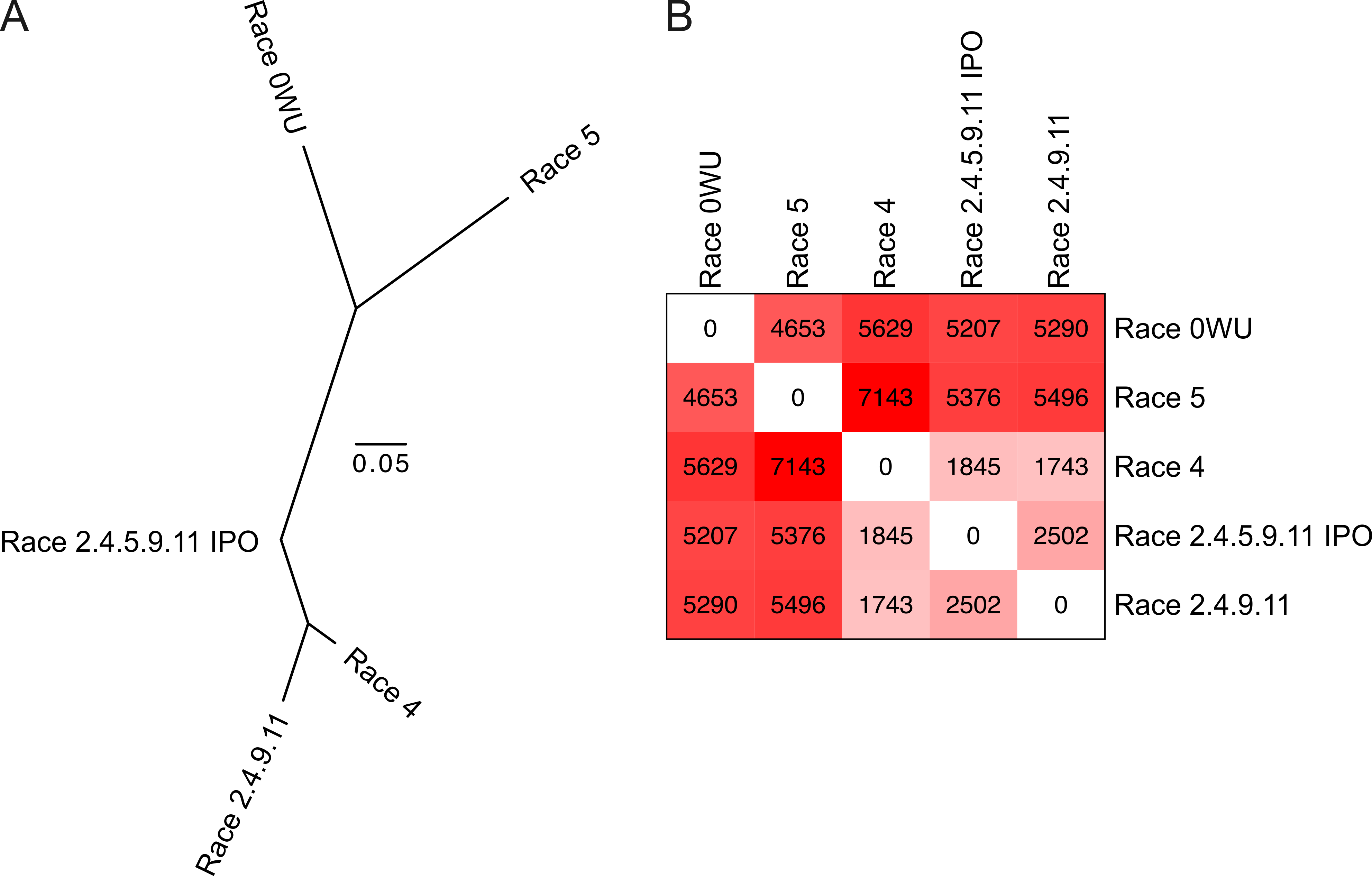

Supplement: Supplementary file 1 — Additional file 1: Fig. S1. Quality of the sequenced PacBio HiFi reads of five Cladosporium fulvum isolates. Fig. S2. The genomes of five Cladosporium fulvum isolates have similar complements of predicted transposable elements (TEs). Fig. S3. The chromosomes of five Cladosporium fulvum isolates are heavily affected by Repeat-Induced Point (RIP) mutations. Fig. S4. Bimodal GC content distribution of five Cladosporium fulvum genomes. Fig. S5. Number of genes encoding carbohydrate-active enzymes (CAZymes) in five Cladosporium fulvum genomes. Fig. S6. Number of genes encoding proteases in five Cladosporium fulvum genomes. Fig. S7. Number of genes encoding cytochrome P450s, transporters, and key enzymes for secondary metabolite biosynthesis (SM) in five Cladosporium fulvum genomes. Fig. S8. Number of genes in five Cladosporium fulvum genomes assigned to different Gene Ontology (GO) terms and EuKaryotic Ortholog Group (KOG) categories. Fig. S9. Overall number of pairwise synteny blocks in pairwise alignments of five Cladosporium fulvum genomes. Fig. S10. Alignment dot plots showing pairwise syntenic regions among Cladosporium fulvum genomes. Fig. S11. Confirmation of large-scale structural variations in the Cladosporium fulvum genomes. Fig. S12. Three large-scale chromosomal structural variations were identified among the five isolates of Cladosporium fulvum. Fig. S13. Comparison of reciprocal translocation events in Cladosporium fulvum and the pine tree pathogen Dothistroma septosporum. Fig. S14. PacBio HiFi reads mapped to the Avr9 locus of Cladosporium fulvum support a non-reciprocal translocation. Fig. S15. The deletion of Avr4E in Cladosporium fulvum likely requires neighboring copies of a Tc1/mariner DNA transposon. Fig. S16. The deletion of Avr5 in Cladosporium fulvum likely requires neighboring copies of a LINE/Tad1 non-LTR retrotransposon. Fig. S17. Most long INDELs in the genome of Cladosporium fulvum are composed of repetitive DNA. Scatter plot showing 1226 IND [file 12915_2024_1818_MOESM1_ESM.zip › Fig_S21_600 bp.tif]

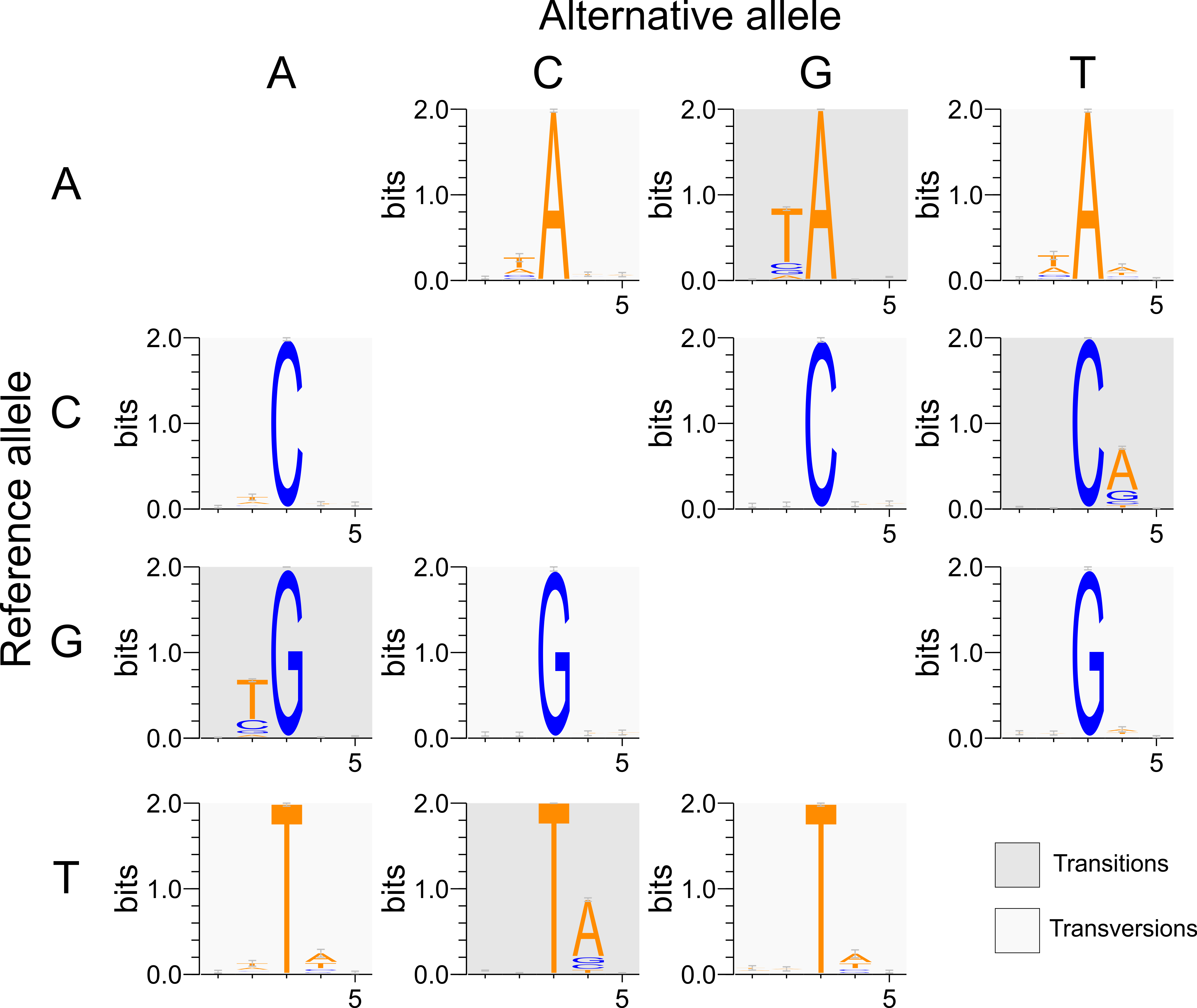

Supplement: Supplementary file 1 — Additional file 1: Fig. S1. Quality of the sequenced PacBio HiFi reads of five Cladosporium fulvum isolates. Fig. S2. The genomes of five Cladosporium fulvum isolates have similar complements of predicted transposable elements (TEs). Fig. S3. The chromosomes of five Cladosporium fulvum isolates are heavily affected by Repeat-Induced Point (RIP) mutations. Fig. S4. Bimodal GC content distribution of five Cladosporium fulvum genomes. Fig. S5. Number of genes encoding carbohydrate-active enzymes (CAZymes) in five Cladosporium fulvum genomes. Fig. S6. Number of genes encoding proteases in five Cladosporium fulvum genomes. Fig. S7. Number of genes encoding cytochrome P450s, transporters, and key enzymes for secondary metabolite biosynthesis (SM) in five Cladosporium fulvum genomes. Fig. S8. Number of genes in five Cladosporium fulvum genomes assigned to different Gene Ontology (GO) terms and EuKaryotic Ortholog Group (KOG) categories. Fig. S9. Overall number of pairwise synteny blocks in pairwise alignments of five Cladosporium fulvum genomes. Fig. S10. Alignment dot plots showing pairwise syntenic regions among Cladosporium fulvum genomes. Fig. S11. Confirmation of large-scale structural variations in the Cladosporium fulvum genomes. Fig. S12. Three large-scale chromosomal structural variations were identified among the five isolates of Cladosporium fulvum. Fig. S13. Comparison of reciprocal translocation events in Cladosporium fulvum and the pine tree pathogen Dothistroma septosporum. Fig. S14. PacBio HiFi reads mapped to the Avr9 locus of Cladosporium fulvum support a non-reciprocal translocation. Fig. S15. The deletion of Avr4E in Cladosporium fulvum likely requires neighboring copies of a Tc1/mariner DNA transposon. Fig. S16. The deletion of Avr5 in Cladosporium fulvum likely requires neighboring copies of a LINE/Tad1 non-LTR retrotransposon. Fig. S17. Most long INDELs in the genome of Cladosporium fulvum are composed of repetitive DNA. Scatter plot showing 1226 IND [file 12915_2024_1818_MOESM1_ESM.zip › Fig_S22B_600 dpi.tif]

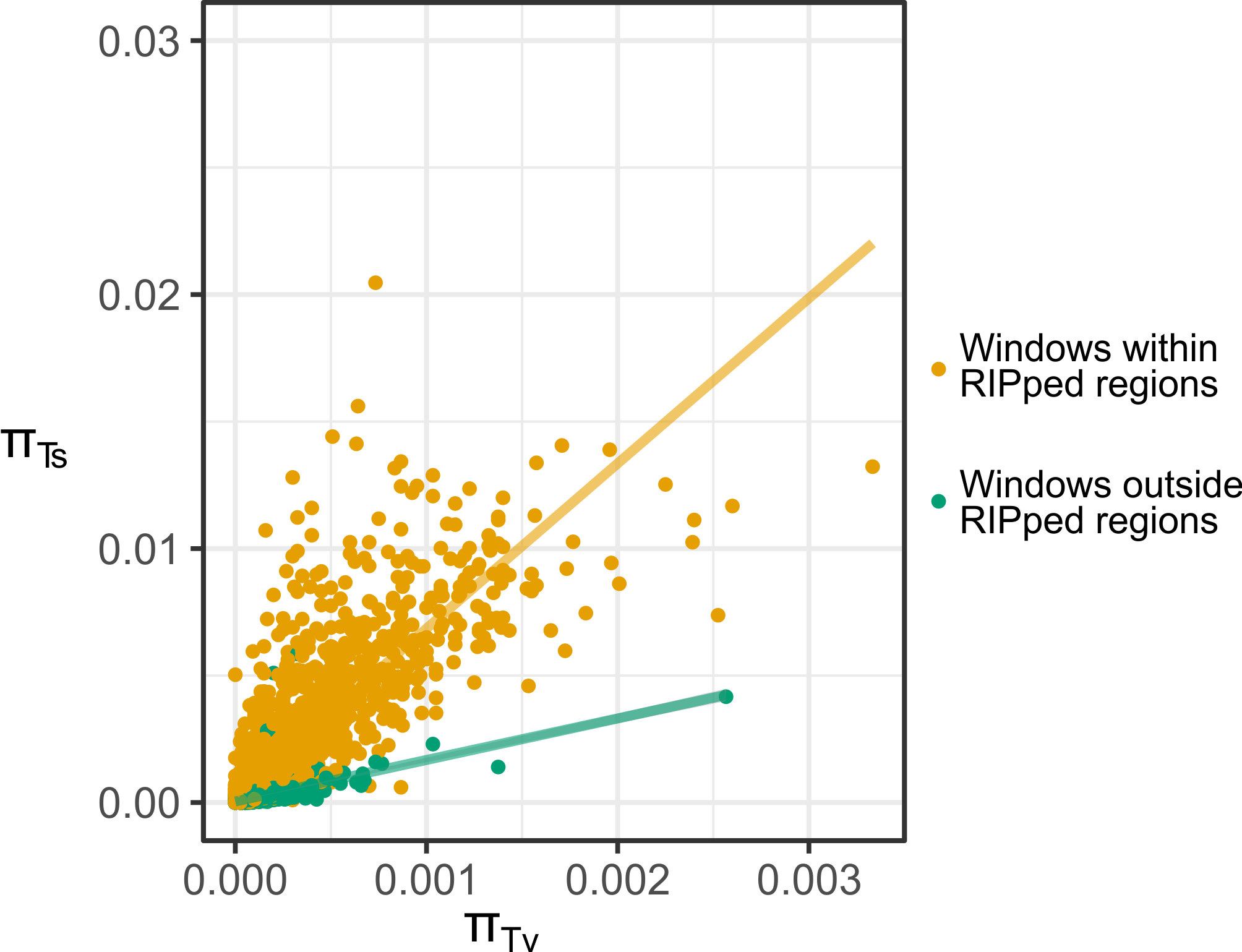

Supplement: Supplementary file 1 — Additional file 1: Fig. S1. Quality of the sequenced PacBio HiFi reads of five Cladosporium fulvum isolates. Fig. S2. The genomes of five Cladosporium fulvum isolates have similar complements of predicted transposable elements (TEs). Fig. S3. The chromosomes of five Cladosporium fulvum isolates are heavily affected by Repeat-Induced Point (RIP) mutations. Fig. S4. Bimodal GC content distribution of five Cladosporium fulvum genomes. Fig. S5. Number of genes encoding carbohydrate-active enzymes (CAZymes) in five Cladosporium fulvum genomes. Fig. S6. Number of genes encoding proteases in five Cladosporium fulvum genomes. Fig. S7. Number of genes encoding cytochrome P450s, transporters, and key enzymes for secondary metabolite biosynthesis (SM) in five Cladosporium fulvum genomes. Fig. S8. Number of genes in five Cladosporium fulvum genomes assigned to different Gene Ontology (GO) terms and EuKaryotic Ortholog Group (KOG) categories. Fig. S9. Overall number of pairwise synteny blocks in pairwise alignments of five Cladosporium fulvum genomes. Fig. S10. Alignment dot plots showing pairwise syntenic regions among Cladosporium fulvum genomes. Fig. S11. Confirmation of large-scale structural variations in the Cladosporium fulvum genomes. Fig. S12. Three large-scale chromosomal structural variations were identified among the five isolates of Cladosporium fulvum. Fig. S13. Comparison of reciprocal translocation events in Cladosporium fulvum and the pine tree pathogen Dothistroma septosporum. Fig. S14. PacBio HiFi reads mapped to the Avr9 locus of Cladosporium fulvum support a non-reciprocal translocation. Fig. S15. The deletion of Avr4E in Cladosporium fulvum likely requires neighboring copies of a Tc1/mariner DNA transposon. Fig. S16. The deletion of Avr5 in Cladosporium fulvum likely requires neighboring copies of a LINE/Tad1 non-LTR retrotransposon. Fig. S17. Most long INDELs in the genome of Cladosporium fulvum are composed of repetitive DNA. Scatter plot showing 1226 IND [file 12915_2024_1818_MOESM1_ESM.zip › Fig_S23B_600 dpi.tif]

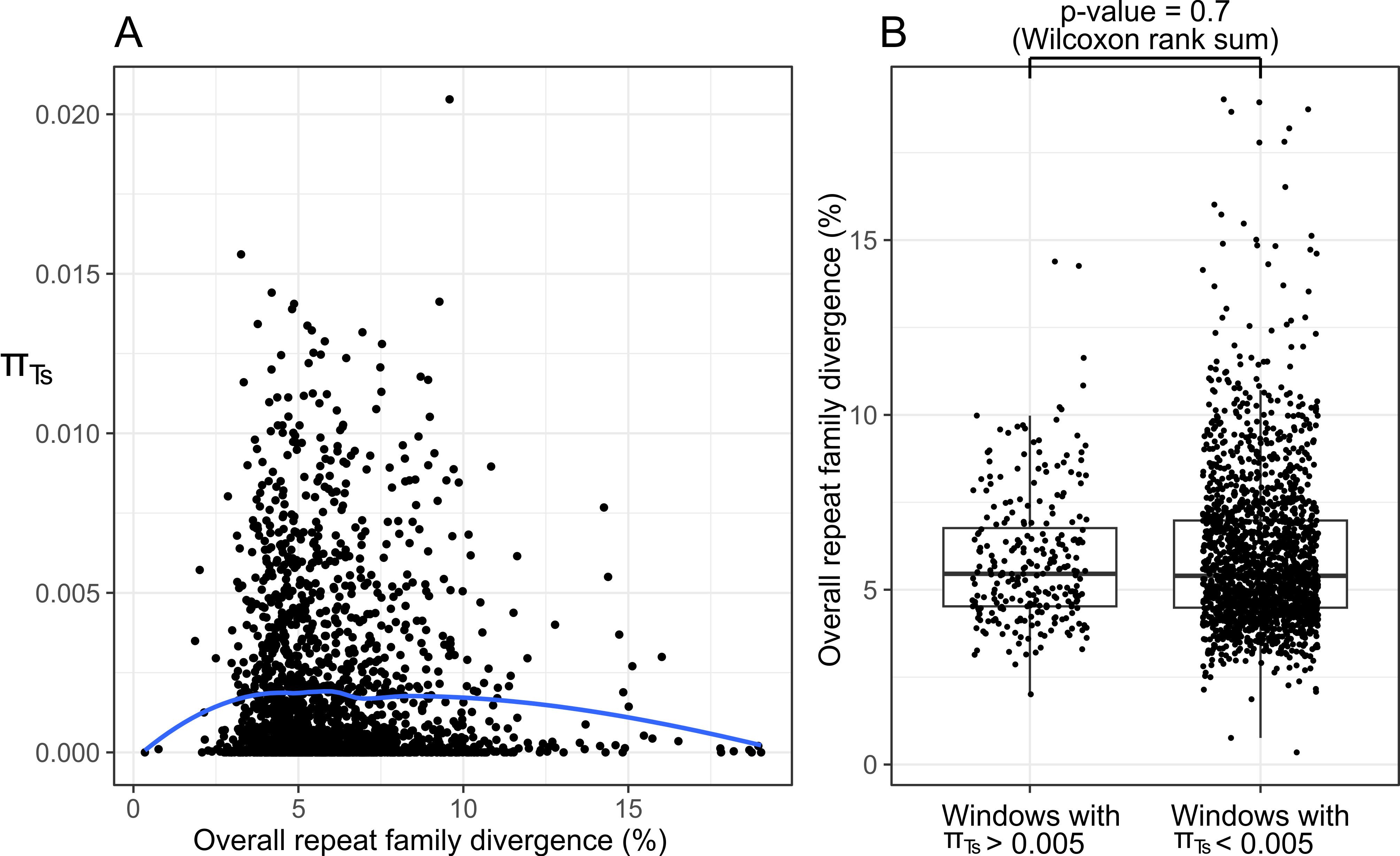

Supplement: Supplementary file 1 — Additional file 1: Fig. S1. Quality of the sequenced PacBio HiFi reads of five Cladosporium fulvum isolates. Fig. S2. The genomes of five Cladosporium fulvum isolates have similar complements of predicted transposable elements (TEs). Fig. S3. The chromosomes of five Cladosporium fulvum isolates are heavily affected by Repeat-Induced Point (RIP) mutations. Fig. S4. Bimodal GC content distribution of five Cladosporium fulvum genomes. Fig. S5. Number of genes encoding carbohydrate-active enzymes (CAZymes) in five Cladosporium fulvum genomes. Fig. S6. Number of genes encoding proteases in five Cladosporium fulvum genomes. Fig. S7. Number of genes encoding cytochrome P450s, transporters, and key enzymes for secondary metabolite biosynthesis (SM) in five Cladosporium fulvum genomes. Fig. S8. Number of genes in five Cladosporium fulvum genomes assigned to different Gene Ontology (GO) terms and EuKaryotic Ortholog Group (KOG) categories. Fig. S9. Overall number of pairwise synteny blocks in pairwise alignments of five Cladosporium fulvum genomes. Fig. S10. Alignment dot plots showing pairwise syntenic regions among Cladosporium fulvum genomes. Fig. S11. Confirmation of large-scale structural variations in the Cladosporium fulvum genomes. Fig. S12. Three large-scale chromosomal structural variations were identified among the five isolates of Cladosporium fulvum. Fig. S13. Comparison of reciprocal translocation events in Cladosporium fulvum and the pine tree pathogen Dothistroma septosporum. Fig. S14. PacBio HiFi reads mapped to the Avr9 locus of Cladosporium fulvum support a non-reciprocal translocation. Fig. S15. The deletion of Avr4E in Cladosporium fulvum likely requires neighboring copies of a Tc1/mariner DNA transposon. Fig. S16. The deletion of Avr5 in Cladosporium fulvum likely requires neighboring copies of a LINE/Tad1 non-LTR retrotransposon. Fig. S17. Most long INDELs in the genome of Cladosporium fulvum are composed of repetitive DNA. Scatter plot showing 1226 IND [file 12915_2024_1818_MOESM1_ESM.zip › Fig_S24B_600 dpi.tif]

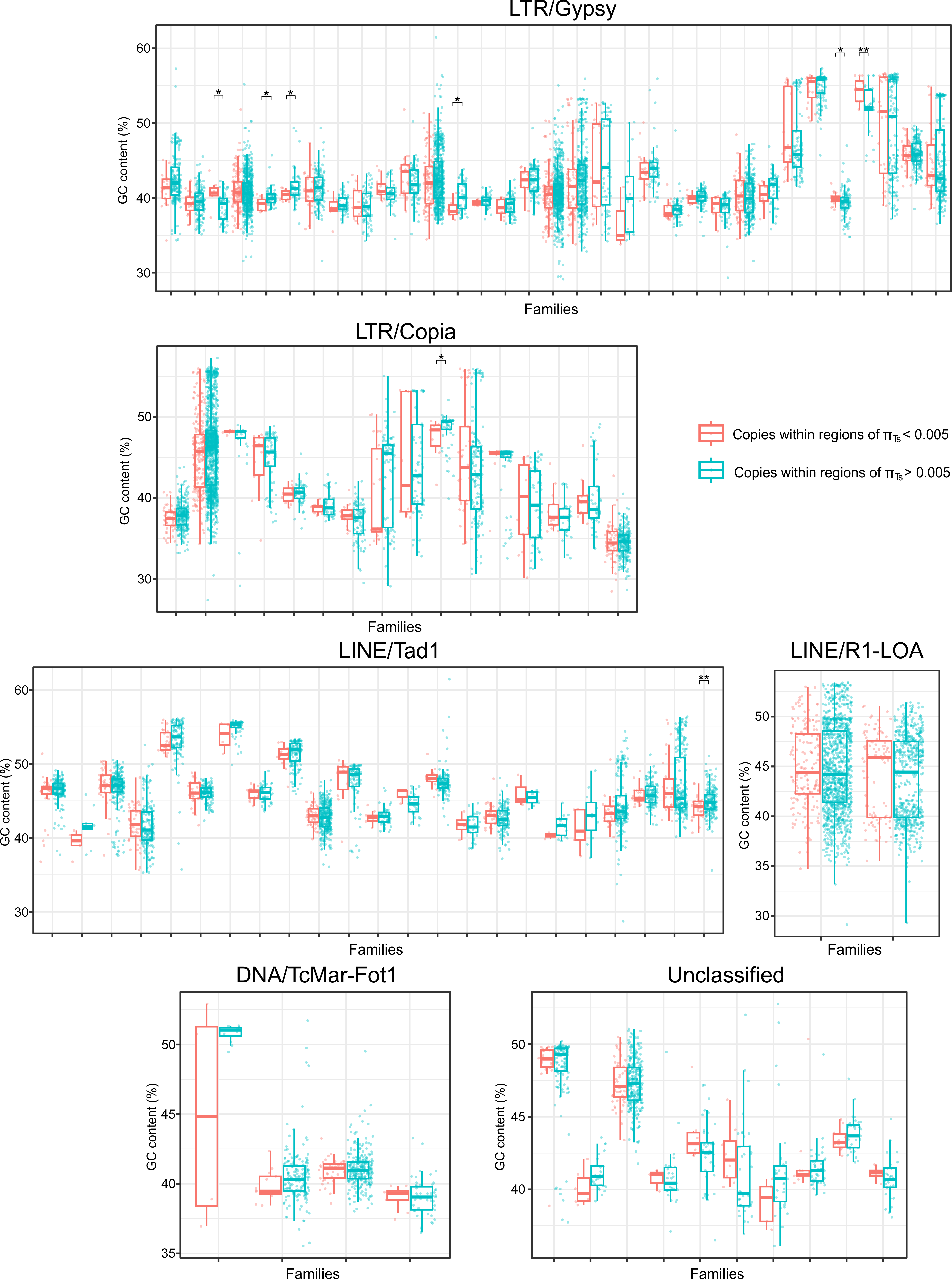

Supplement: Supplementary file 1 — Additional file 1: Fig. S1. Quality of the sequenced PacBio HiFi reads of five Cladosporium fulvum isolates. Fig. S2. The genomes of five Cladosporium fulvum isolates have similar complements of predicted transposable elements (TEs). Fig. S3. The chromosomes of five Cladosporium fulvum isolates are heavily affected by Repeat-Induced Point (RIP) mutations. Fig. S4. Bimodal GC content distribution of five Cladosporium fulvum genomes. Fig. S5. Number of genes encoding carbohydrate-active enzymes (CAZymes) in five Cladosporium fulvum genomes. Fig. S6. Number of genes encoding proteases in five Cladosporium fulvum genomes. Fig. S7. Number of genes encoding cytochrome P450s, transporters, and key enzymes for secondary metabolite biosynthesis (SM) in five Cladosporium fulvum genomes. Fig. S8. Number of genes in five Cladosporium fulvum genomes assigned to different Gene Ontology (GO) terms and EuKaryotic Ortholog Group (KOG) categories. Fig. S9. Overall number of pairwise synteny blocks in pairwise alignments of five Cladosporium fulvum genomes. Fig. S10. Alignment dot plots showing pairwise syntenic regions among Cladosporium fulvum genomes. Fig. S11. Confirmation of large-scale structural variations in the Cladosporium fulvum genomes. Fig. S12. Three large-scale chromosomal structural variations were identified among the five isolates of Cladosporium fulvum. Fig. S13. Comparison of reciprocal translocation events in Cladosporium fulvum and the pine tree pathogen Dothistroma septosporum. Fig. S14. PacBio HiFi reads mapped to the Avr9 locus of Cladosporium fulvum support a non-reciprocal translocation. Fig. S15. The deletion of Avr4E in Cladosporium fulvum likely requires neighboring copies of a Tc1/mariner DNA transposon. Fig. S16. The deletion of Avr5 in Cladosporium fulvum likely requires neighboring copies of a LINE/Tad1 non-LTR retrotransposon. Fig. S17. Most long INDELs in the genome of Cladosporium fulvum are composed of repetitive DNA. Scatter plot showing 1226 IND [file 12915_2024_1818_MOESM1_ESM.zip › Fig_S25_600 dpi.tif]

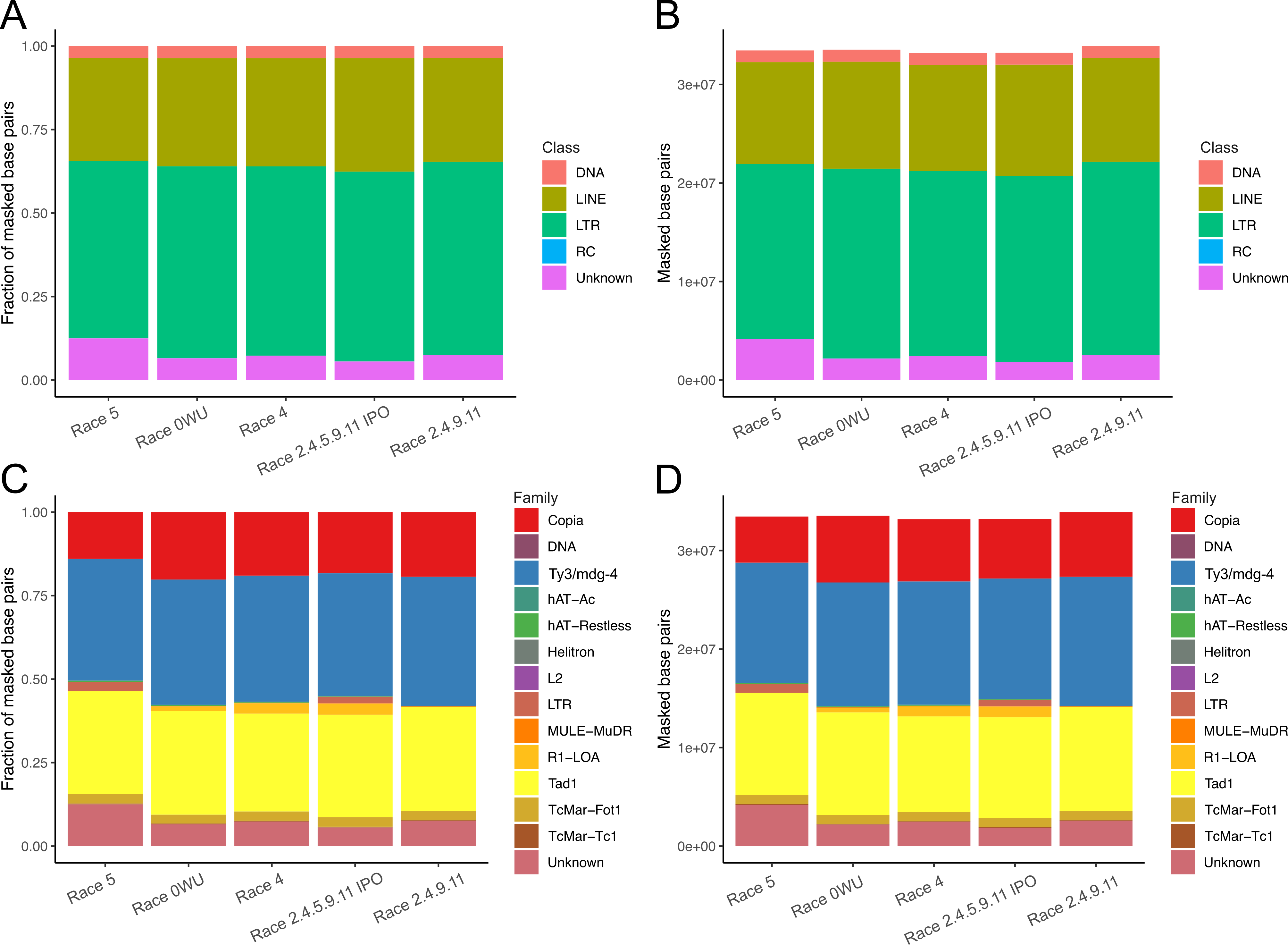

Supplement: Supplementary file 1 — Additional file 1: Fig. S1. Quality of the sequenced PacBio HiFi reads of five Cladosporium fulvum isolates. Fig. S2. The genomes of five Cladosporium fulvum isolates have similar complements of predicted transposable elements (TEs). Fig. S3. The chromosomes of five Cladosporium fulvum isolates are heavily affected by Repeat-Induced Point (RIP) mutations. Fig. S4. Bimodal GC content distribution of five Cladosporium fulvum genomes. Fig. S5. Number of genes encoding carbohydrate-active enzymes (CAZymes) in five Cladosporium fulvum genomes. Fig. S6. Number of genes encoding proteases in five Cladosporium fulvum genomes. Fig. S7. Number of genes encoding cytochrome P450s, transporters, and key enzymes for secondary metabolite biosynthesis (SM) in five Cladosporium fulvum genomes. Fig. S8. Number of genes in five Cladosporium fulvum genomes assigned to different Gene Ontology (GO) terms and EuKaryotic Ortholog Group (KOG) categories. Fig. S9. Overall number of pairwise synteny blocks in pairwise alignments of five Cladosporium fulvum genomes. Fig. S10. Alignment dot plots showing pairwise syntenic regions among Cladosporium fulvum genomes. Fig. S11. Confirmation of large-scale structural variations in the Cladosporium fulvum genomes. Fig. S12. Three large-scale chromosomal structural variations were identified among the five isolates of Cladosporium fulvum. Fig. S13. Comparison of reciprocal translocation events in Cladosporium fulvum and the pine tree pathogen Dothistroma septosporum. Fig. S14. PacBio HiFi reads mapped to the Avr9 locus of Cladosporium fulvum support a non-reciprocal translocation. Fig. S15. The deletion of Avr4E in Cladosporium fulvum likely requires neighboring copies of a Tc1/mariner DNA transposon. Fig. S16. The deletion of Avr5 in Cladosporium fulvum likely requires neighboring copies of a LINE/Tad1 non-LTR retrotransposon. Fig. S17. Most long INDELs in the genome of Cladosporium fulvum are composed of repetitive DNA. Scatter plot showing 1226 IND [file 12915_2024_1818_MOESM1_ESM.zip › Fig_S2B_600 bp.tif]

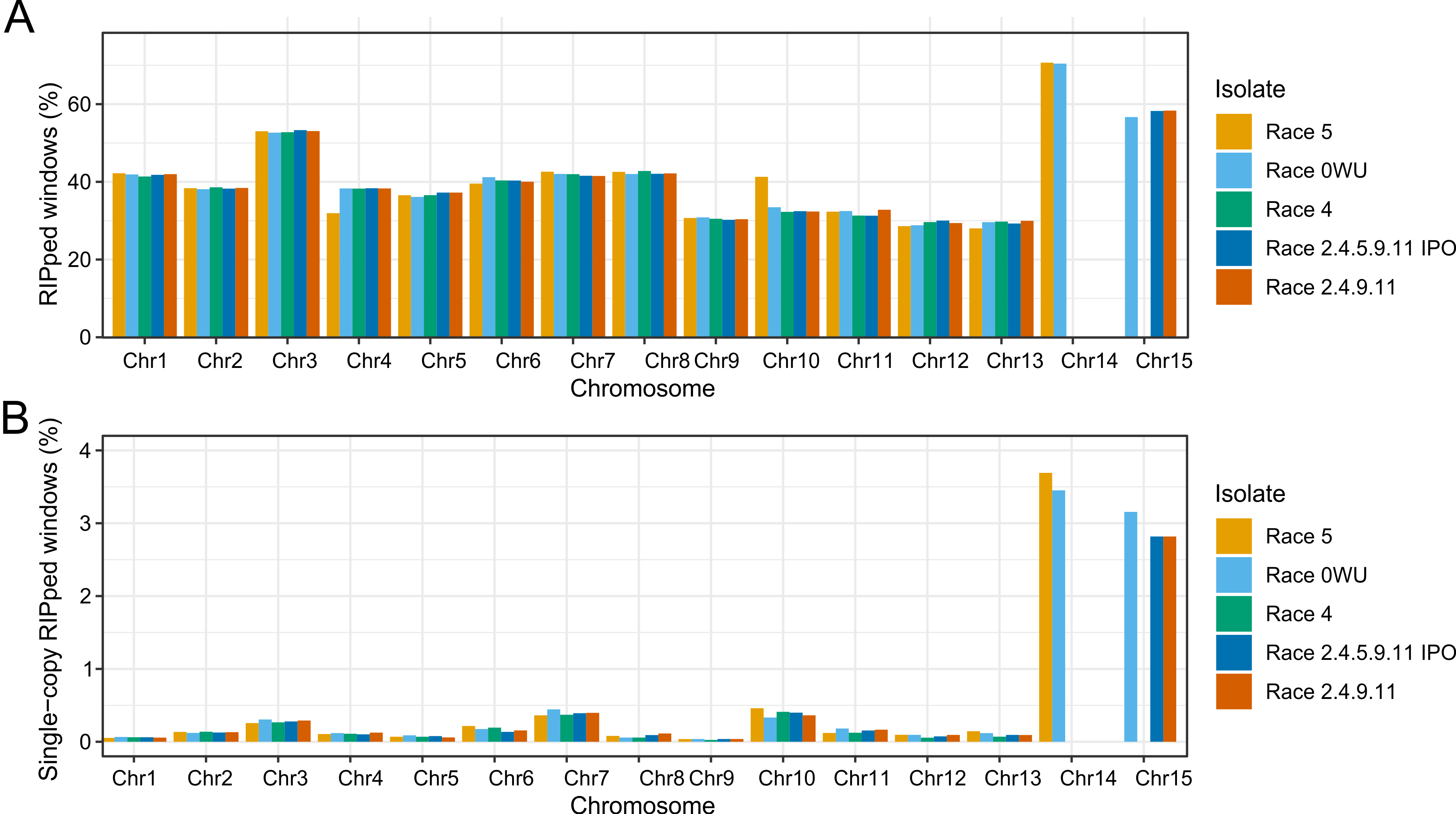

Supplement: Supplementary file 1 — Additional file 1: Fig. S1. Quality of the sequenced PacBio HiFi reads of five Cladosporium fulvum isolates. Fig. S2. The genomes of five Cladosporium fulvum isolates have similar complements of predicted transposable elements (TEs). Fig. S3. The chromosomes of five Cladosporium fulvum isolates are heavily affected by Repeat-Induced Point (RIP) mutations. Fig. S4. Bimodal GC content distribution of five Cladosporium fulvum genomes. Fig. S5. Number of genes encoding carbohydrate-active enzymes (CAZymes) in five Cladosporium fulvum genomes. Fig. S6. Number of genes encoding proteases in five Cladosporium fulvum genomes. Fig. S7. Number of genes encoding cytochrome P450s, transporters, and key enzymes for secondary metabolite biosynthesis (SM) in five Cladosporium fulvum genomes. Fig. S8. Number of genes in five Cladosporium fulvum genomes assigned to different Gene Ontology (GO) terms and EuKaryotic Ortholog Group (KOG) categories. Fig. S9. Overall number of pairwise synteny blocks in pairwise alignments of five Cladosporium fulvum genomes. Fig. S10. Alignment dot plots showing pairwise syntenic regions among Cladosporium fulvum genomes. Fig. S11. Confirmation of large-scale structural variations in the Cladosporium fulvum genomes. Fig. S12. Three large-scale chromosomal structural variations were identified among the five isolates of Cladosporium fulvum. Fig. S13. Comparison of reciprocal translocation events in Cladosporium fulvum and the pine tree pathogen Dothistroma septosporum. Fig. S14. PacBio HiFi reads mapped to the Avr9 locus of Cladosporium fulvum support a non-reciprocal translocation. Fig. S15. The deletion of Avr4E in Cladosporium fulvum likely requires neighboring copies of a Tc1/mariner DNA transposon. Fig. S16. The deletion of Avr5 in Cladosporium fulvum likely requires neighboring copies of a LINE/Tad1 non-LTR retrotransposon. Fig. S17. Most long INDELs in the genome of Cladosporium fulvum are composed of repetitive DNA. Scatter plot showing 1226 IND [file 12915_2024_1818_MOESM1_ESM.zip › Fig_S3B_600 bp.tif]

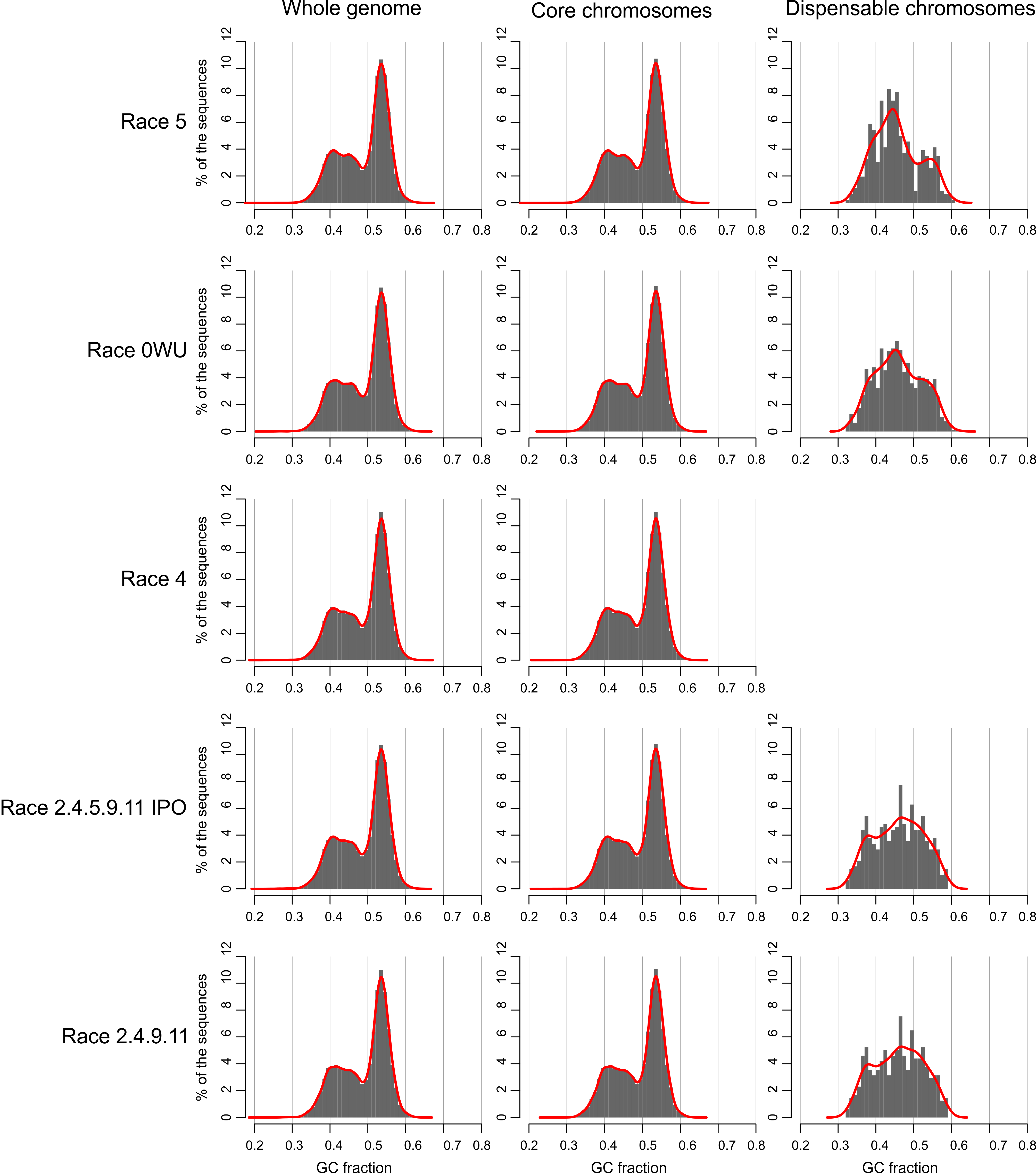

Supplement: Supplementary file 1 — Additional file 1: Fig. S1. Quality of the sequenced PacBio HiFi reads of five Cladosporium fulvum isolates. Fig. S2. The genomes of five Cladosporium fulvum isolates have similar complements of predicted transposable elements (TEs). Fig. S3. The chromosomes of five Cladosporium fulvum isolates are heavily affected by Repeat-Induced Point (RIP) mutations. Fig. S4. Bimodal GC content distribution of five Cladosporium fulvum genomes. Fig. S5. Number of genes encoding carbohydrate-active enzymes (CAZymes) in five Cladosporium fulvum genomes. Fig. S6. Number of genes encoding proteases in five Cladosporium fulvum genomes. Fig. S7. Number of genes encoding cytochrome P450s, transporters, and key enzymes for secondary metabolite biosynthesis (SM) in five Cladosporium fulvum genomes. Fig. S8. Number of genes in five Cladosporium fulvum genomes assigned to different Gene Ontology (GO) terms and EuKaryotic Ortholog Group (KOG) categories. Fig. S9. Overall number of pairwise synteny blocks in pairwise alignments of five Cladosporium fulvum genomes. Fig. S10. Alignment dot plots showing pairwise syntenic regions among Cladosporium fulvum genomes. Fig. S11. Confirmation of large-scale structural variations in the Cladosporium fulvum genomes. Fig. S12. Three large-scale chromosomal structural variations were identified among the five isolates of Cladosporium fulvum. Fig. S13. Comparison of reciprocal translocation events in Cladosporium fulvum and the pine tree pathogen Dothistroma septosporum. Fig. S14. PacBio HiFi reads mapped to the Avr9 locus of Cladosporium fulvum support a non-reciprocal translocation. Fig. S15. The deletion of Avr4E in Cladosporium fulvum likely requires neighboring copies of a Tc1/mariner DNA transposon. Fig. S16. The deletion of Avr5 in Cladosporium fulvum likely requires neighboring copies of a LINE/Tad1 non-LTR retrotransposon. Fig. S17. Most long INDELs in the genome of Cladosporium fulvum are composed of repetitive DNA. Scatter plot showing 1226 IND [file 12915_2024_1818_MOESM1_ESM.zip › Fig_S4B_600 bp.tif]

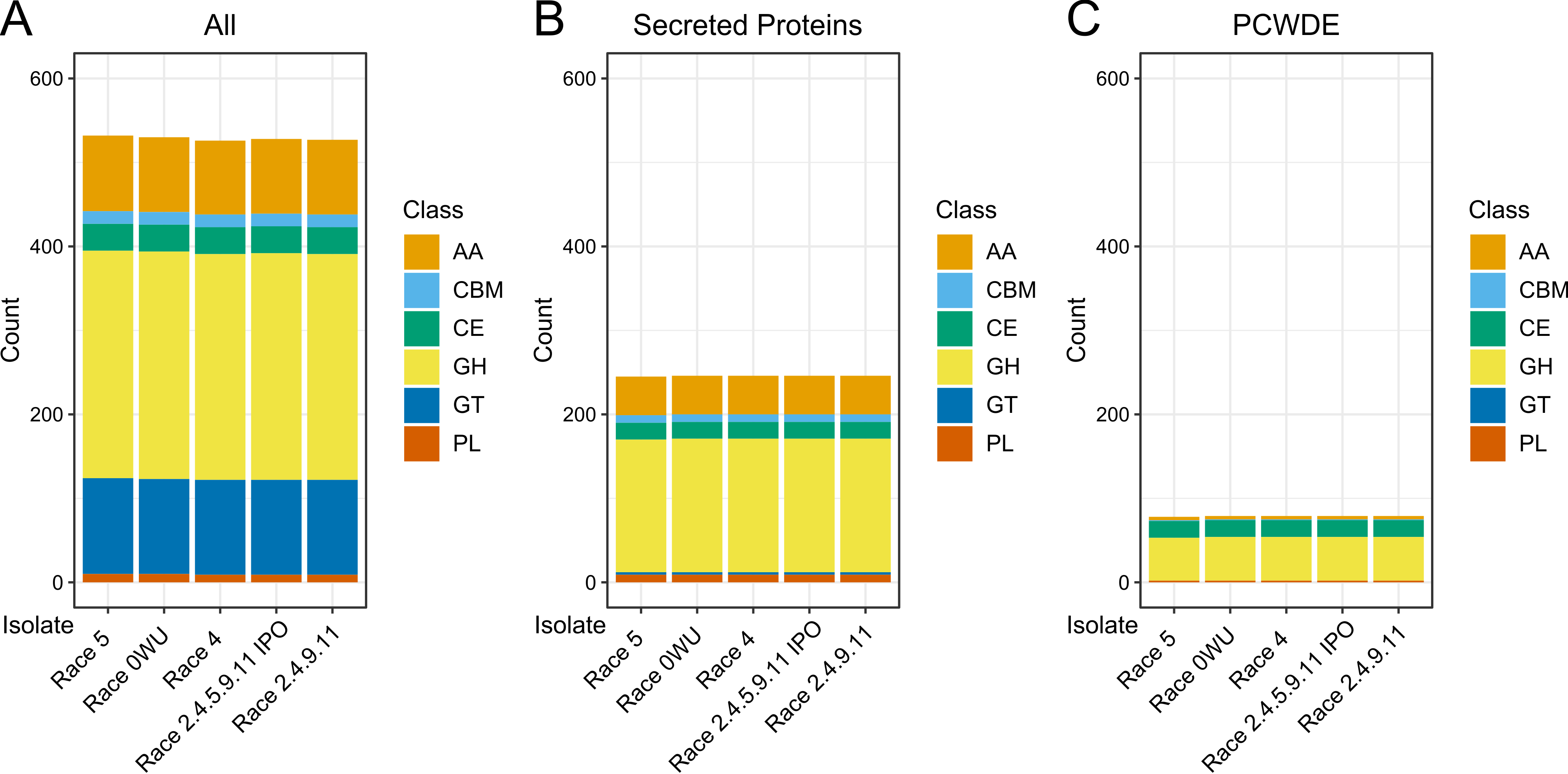

Supplement: Supplementary file 1 — Additional file 1: Fig. S1. Quality of the sequenced PacBio HiFi reads of five Cladosporium fulvum isolates. Fig. S2. The genomes of five Cladosporium fulvum isolates have similar complements of predicted transposable elements (TEs). Fig. S3. The chromosomes of five Cladosporium fulvum isolates are heavily affected by Repeat-Induced Point (RIP) mutations. Fig. S4. Bimodal GC content distribution of five Cladosporium fulvum genomes. Fig. S5. Number of genes encoding carbohydrate-active enzymes (CAZymes) in five Cladosporium fulvum genomes. Fig. S6. Number of genes encoding proteases in five Cladosporium fulvum genomes. Fig. S7. Number of genes encoding cytochrome P450s, transporters, and key enzymes for secondary metabolite biosynthesis (SM) in five Cladosporium fulvum genomes. Fig. S8. Number of genes in five Cladosporium fulvum genomes assigned to different Gene Ontology (GO) terms and EuKaryotic Ortholog Group (KOG) categories. Fig. S9. Overall number of pairwise synteny blocks in pairwise alignments of five Cladosporium fulvum genomes. Fig. S10. Alignment dot plots showing pairwise syntenic regions among Cladosporium fulvum genomes. Fig. S11. Confirmation of large-scale structural variations in the Cladosporium fulvum genomes. Fig. S12. Three large-scale chromosomal structural variations were identified among the five isolates of Cladosporium fulvum. Fig. S13. Comparison of reciprocal translocation events in Cladosporium fulvum and the pine tree pathogen Dothistroma septosporum. Fig. S14. PacBio HiFi reads mapped to the Avr9 locus of Cladosporium fulvum support a non-reciprocal translocation. Fig. S15. The deletion of Avr4E in Cladosporium fulvum likely requires neighboring copies of a Tc1/mariner DNA transposon. Fig. S16. The deletion of Avr5 in Cladosporium fulvum likely requires neighboring copies of a LINE/Tad1 non-LTR retrotransposon. Fig. S17. Most long INDELs in the genome of Cladosporium fulvum are composed of repetitive DNA. Scatter plot showing 1226 IND [file 12915_2024_1818_MOESM1_ESM.zip › Fig_S5B_600 bp.tif]

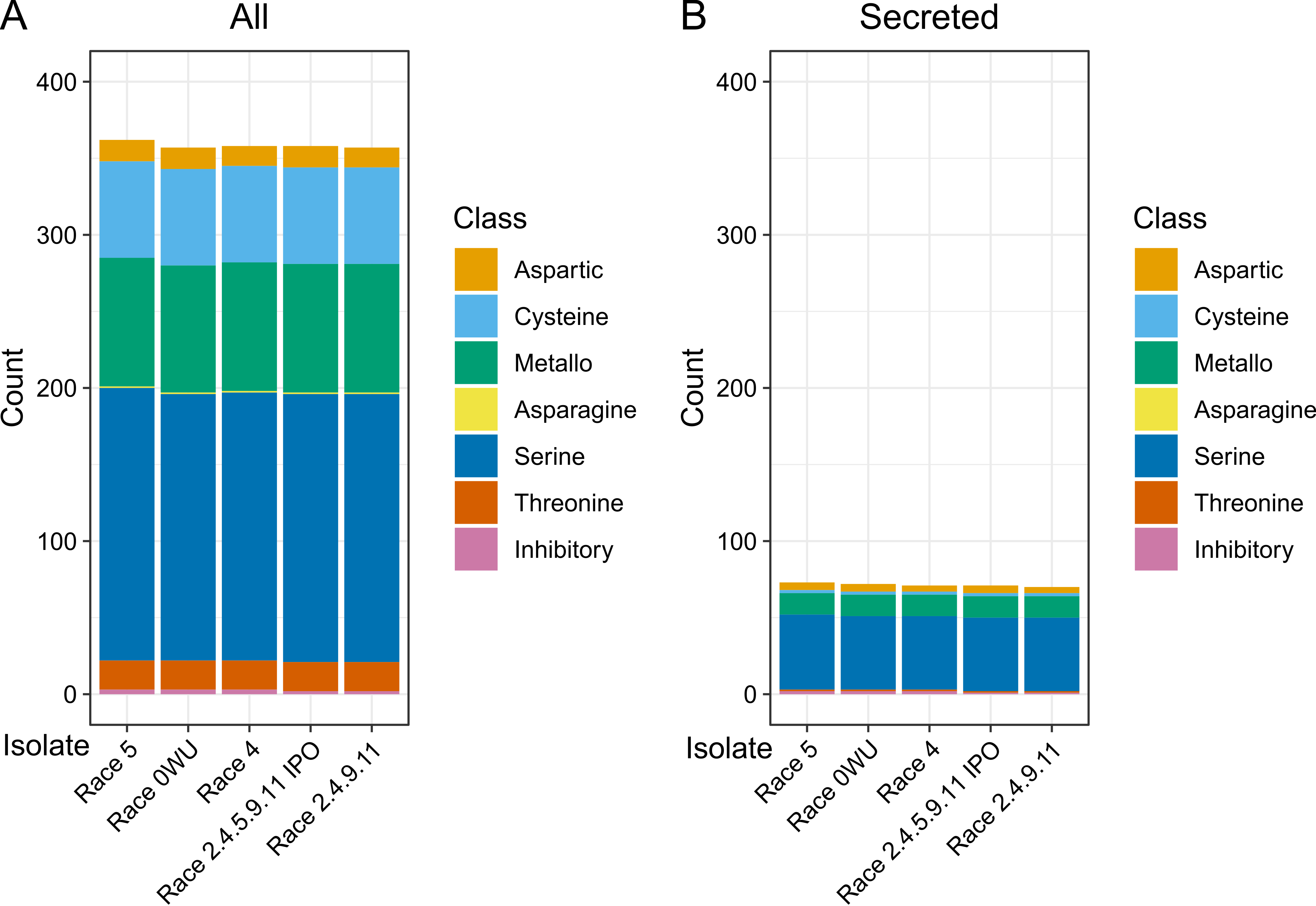

Supplement: Supplementary file 1 — Additional file 1: Fig. S1. Quality of the sequenced PacBio HiFi reads of five Cladosporium fulvum isolates. Fig. S2. The genomes of five Cladosporium fulvum isolates have similar complements of predicted transposable elements (TEs). Fig. S3. The chromosomes of five Cladosporium fulvum isolates are heavily affected by Repeat-Induced Point (RIP) mutations. Fig. S4. Bimodal GC content distribution of five Cladosporium fulvum genomes. Fig. S5. Number of genes encoding carbohydrate-active enzymes (CAZymes) in five Cladosporium fulvum genomes. Fig. S6. Number of genes encoding proteases in five Cladosporium fulvum genomes. Fig. S7. Number of genes encoding cytochrome P450s, transporters, and key enzymes for secondary metabolite biosynthesis (SM) in five Cladosporium fulvum genomes. Fig. S8. Number of genes in five Cladosporium fulvum genomes assigned to different Gene Ontology (GO) terms and EuKaryotic Ortholog Group (KOG) categories. Fig. S9. Overall number of pairwise synteny blocks in pairwise alignments of five Cladosporium fulvum genomes. Fig. S10. Alignment dot plots showing pairwise syntenic regions among Cladosporium fulvum genomes. Fig. S11. Confirmation of large-scale structural variations in the Cladosporium fulvum genomes. Fig. S12. Three large-scale chromosomal structural variations were identified among the five isolates of Cladosporium fulvum. Fig. S13. Comparison of reciprocal translocation events in Cladosporium fulvum and the pine tree pathogen Dothistroma septosporum. Fig. S14. PacBio HiFi reads mapped to the Avr9 locus of Cladosporium fulvum support a non-reciprocal translocation. Fig. S15. The deletion of Avr4E in Cladosporium fulvum likely requires neighboring copies of a Tc1/mariner DNA transposon. Fig. S16. The deletion of Avr5 in Cladosporium fulvum likely requires neighboring copies of a LINE/Tad1 non-LTR retrotransposon. Fig. S17. Most long INDELs in the genome of Cladosporium fulvum are composed of repetitive DNA. Scatter plot showing 1226 IND [file 12915_2024_1818_MOESM1_ESM.zip › Fig_S6B_600 bp.tif]

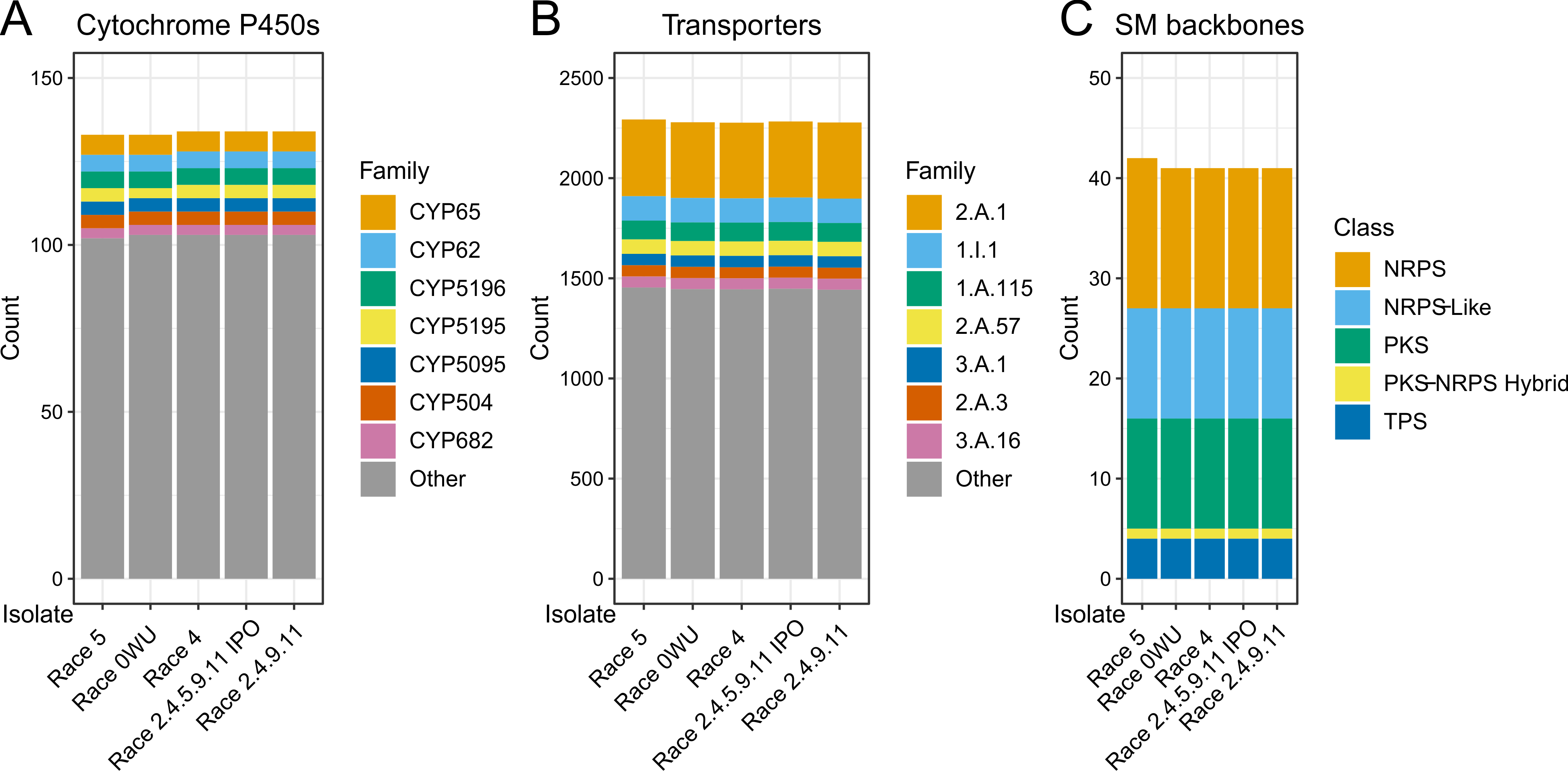

Supplement: Supplementary file 1 — Additional file 1: Fig. S1. Quality of the sequenced PacBio HiFi reads of five Cladosporium fulvum isolates. Fig. S2. The genomes of five Cladosporium fulvum isolates have similar complements of predicted transposable elements (TEs). Fig. S3. The chromosomes of five Cladosporium fulvum isolates are heavily affected by Repeat-Induced Point (RIP) mutations. Fig. S4. Bimodal GC content distribution of five Cladosporium fulvum genomes. Fig. S5. Number of genes encoding carbohydrate-active enzymes (CAZymes) in five Cladosporium fulvum genomes. Fig. S6. Number of genes encoding proteases in five Cladosporium fulvum genomes. Fig. S7. Number of genes encoding cytochrome P450s, transporters, and key enzymes for secondary metabolite biosynthesis (SM) in five Cladosporium fulvum genomes. Fig. S8. Number of genes in five Cladosporium fulvum genomes assigned to different Gene Ontology (GO) terms and EuKaryotic Ortholog Group (KOG) categories. Fig. S9. Overall number of pairwise synteny blocks in pairwise alignments of five Cladosporium fulvum genomes. Fig. S10. Alignment dot plots showing pairwise syntenic regions among Cladosporium fulvum genomes. Fig. S11. Confirmation of large-scale structural variations in the Cladosporium fulvum genomes. Fig. S12. Three large-scale chromosomal structural variations were identified among the five isolates of Cladosporium fulvum. Fig. S13. Comparison of reciprocal translocation events in Cladosporium fulvum and the pine tree pathogen Dothistroma septosporum. Fig. S14. PacBio HiFi reads mapped to the Avr9 locus of Cladosporium fulvum support a non-reciprocal translocation. Fig. S15. The deletion of Avr4E in Cladosporium fulvum likely requires neighboring copies of a Tc1/mariner DNA transposon. Fig. S16. The deletion of Avr5 in Cladosporium fulvum likely requires neighboring copies of a LINE/Tad1 non-LTR retrotransposon. Fig. S17. Most long INDELs in the genome of Cladosporium fulvum are composed of repetitive DNA. Scatter plot showing 1226 IND [file 12915_2024_1818_MOESM1_ESM.zip › Fig_S7B_600 bp.tif]

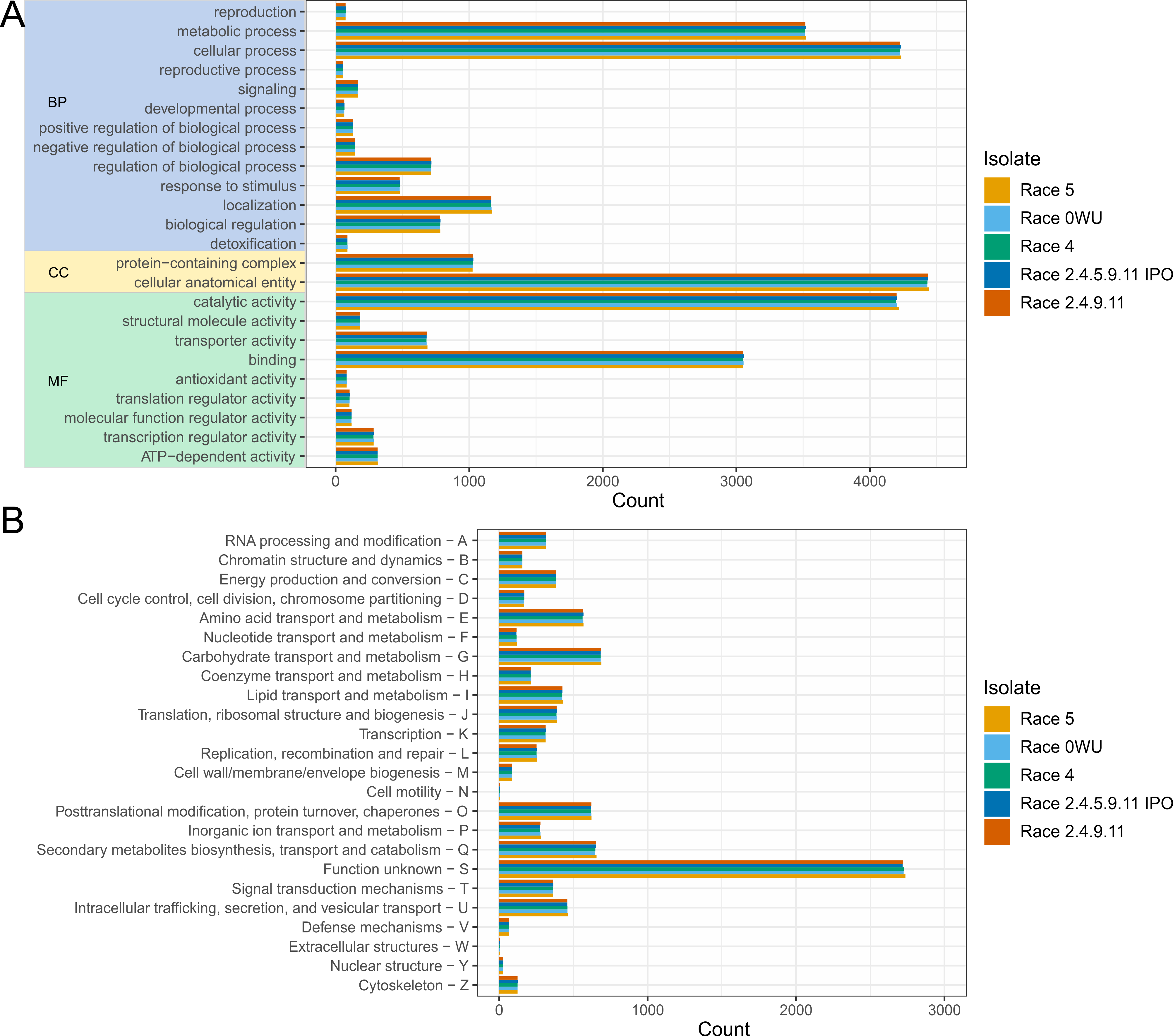

Supplement: Supplementary file 1 — Additional file 1: Fig. S1. Quality of the sequenced PacBio HiFi reads of five Cladosporium fulvum isolates. Fig. S2. The genomes of five Cladosporium fulvum isolates have similar complements of predicted transposable elements (TEs). Fig. S3. The chromosomes of five Cladosporium fulvum isolates are heavily affected by Repeat-Induced Point (RIP) mutations. Fig. S4. Bimodal GC content distribution of five Cladosporium fulvum genomes. Fig. S5. Number of genes encoding carbohydrate-active enzymes (CAZymes) in five Cladosporium fulvum genomes. Fig. S6. Number of genes encoding proteases in five Cladosporium fulvum genomes. Fig. S7. Number of genes encoding cytochrome P450s, transporters, and key enzymes for secondary metabolite biosynthesis (SM) in five Cladosporium fulvum genomes. Fig. S8. Number of genes in five Cladosporium fulvum genomes assigned to different Gene Ontology (GO) terms and EuKaryotic Ortholog Group (KOG) categories. Fig. S9. Overall number of pairwise synteny blocks in pairwise alignments of five Cladosporium fulvum genomes. Fig. S10. Alignment dot plots showing pairwise syntenic regions among Cladosporium fulvum genomes. Fig. S11. Confirmation of large-scale structural variations in the Cladosporium fulvum genomes. Fig. S12. Three large-scale chromosomal structural variations were identified among the five isolates of Cladosporium fulvum. Fig. S13. Comparison of reciprocal translocation events in Cladosporium fulvum and the pine tree pathogen Dothistroma septosporum. Fig. S14. PacBio HiFi reads mapped to the Avr9 locus of Cladosporium fulvum support a non-reciprocal translocation. Fig. S15. The deletion of Avr4E in Cladosporium fulvum likely requires neighboring copies of a Tc1/mariner DNA transposon. Fig. S16. The deletion of Avr5 in Cladosporium fulvum likely requires neighboring copies of a LINE/Tad1 non-LTR retrotransposon. Fig. S17. Most long INDELs in the genome of Cladosporium fulvum are composed of repetitive DNA. Scatter plot showing 1226 IND [file 12915_2024_1818_MOESM1_ESM.zip › Fig_S8B_600 dpi.tif]

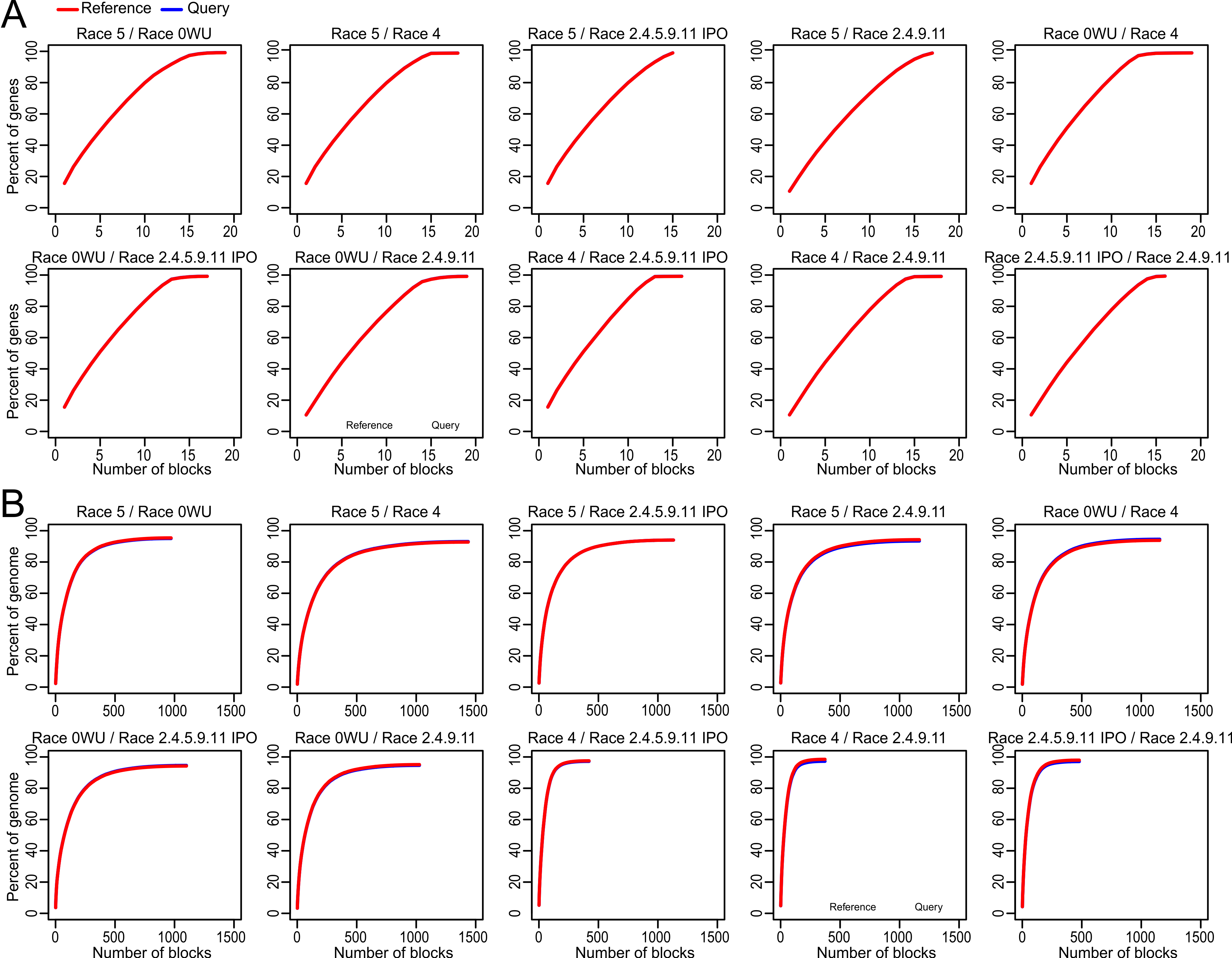

Supplement: Supplementary file 1 — Additional file 1: Fig. S1. Quality of the sequenced PacBio HiFi reads of five Cladosporium fulvum isolates. Fig. S2. The genomes of five Cladosporium fulvum isolates have similar complements of predicted transposable elements (TEs). Fig. S3. The chromosomes of five Cladosporium fulvum isolates are heavily affected by Repeat-Induced Point (RIP) mutations. Fig. S4. Bimodal GC content distribution of five Cladosporium fulvum genomes. Fig. S5. Number of genes encoding carbohydrate-active enzymes (CAZymes) in five Cladosporium fulvum genomes. Fig. S6. Number of genes encoding proteases in five Cladosporium fulvum genomes. Fig. S7. Number of genes encoding cytochrome P450s, transporters, and key enzymes for secondary metabolite biosynthesis (SM) in five Cladosporium fulvum genomes. Fig. S8. Number of genes in five Cladosporium fulvum genomes assigned to different Gene Ontology (GO) terms and EuKaryotic Ortholog Group (KOG) categories. Fig. S9. Overall number of pairwise synteny blocks in pairwise alignments of five Cladosporium fulvum genomes. Fig. S10. Alignment dot plots showing pairwise syntenic regions among Cladosporium fulvum genomes. Fig. S11. Confirmation of large-scale structural variations in the Cladosporium fulvum genomes. Fig. S12. Three large-scale chromosomal structural variations were identified among the five isolates of Cladosporium fulvum. Fig. S13. Comparison of reciprocal translocation events in Cladosporium fulvum and the pine tree pathogen Dothistroma septosporum. Fig. S14. PacBio HiFi reads mapped to the Avr9 locus of Cladosporium fulvum support a non-reciprocal translocation. Fig. S15. The deletion of Avr4E in Cladosporium fulvum likely requires neighboring copies of a Tc1/mariner DNA transposon. Fig. S16. The deletion of Avr5 in Cladosporium fulvum likely requires neighboring copies of a LINE/Tad1 non-LTR retrotransposon. Fig. S17. Most long INDELs in the genome of Cladosporium fulvum are composed of repetitive DNA. Scatter plot showing 1226 IND [file 12915_2024_1818_MOESM1_ESM.zip › Fig_S9B_600 dpi.tif]
